# High-performance multijunction perovskite LEDs with reduced interconnection loss

**DOI:** 10.1038/s41467-026-75756-5

**Published:** 2026-07-17

**Authors:** Feiyun Zhao, Haifeng Zhao, Zhennan Tian, Aobo Ren, Lechuan Chen, Hengyu Liu, Jun Wu, Kai Shen, Wuyang Ren, Chuang Li, Chunyang Yin, Junsheng Yu, Feng Gao, Sai Bai, Jiang Wu, Yanrong Li

**Affiliations:** 1 Institute of Fundamental and Frontier Sciences, University of Electronic Science and Technology of China, Chengdu, China; 2 Southwest Institute of Technical Physics, Chengdu, China; 3 Yibin Institute of UESTC, University of Electronic Science and Technology of China (UESTC), Yibin, China; 4 School of Optoelectronic Science and Engineering, University of Electronic Science and Technology of China, Chengdu, China; 5 State Key Laboratory of Electronic Thin Films and Integrated Devices, University of Electronic Science and Technology of China, Chengdu, China; 6 Department of Physics, Chemistry and Biology, Linköping University, Linköping, Sweden; 7 Northwestern Polytechnical University, Xi’an, China

**Keywords:** Lasers, LEDs and light sources, Materials for devices

## Abstract

Tandem light-emitting diodes (LEDs) offer advantages in efficiency, brightness and lower the current density for a target brightness, but their implementation in solution-processed perovskite LEDs is limited by interconnection strategies that introduce high voltage penalties and poor robustness. Here we demonstrate a hierarchically structured interconnection layer (ICL) integrating a near-degenerate charge-generation junction with a crystalline-amorphous oxide composite, enabling low-barrier transport and solvent-tolerant stacking with reduced loss. Using this ICL, we realize double-junction perovskite LEDs in which two subunits operate efficiently in series, achieving a peak external quantum efficiency (EQE) of 42.6%, a maximum radiance of 690 W sr^−1^ m^−2^, and low driving voltage. The ICL is compatible with multiple perovskite emitters and supports scalable multijunction integration, yielding multijunction devices with sub-bandgap turn-on voltages and high EQEs exceeding 60%. Our results establish a low-loss interconnection strategy for series-stacked perovskite emitters, providing a practical route toward high-brightness and multi-wavelength perovskite light sources.

## Abstract (web summary)

Tandem perovskite light-emitting diodes offer advantages for improving device performance but are limited by lossy, unstable interconnections. Zhao et al. design a robust layered interconnection enabling efficient stacked perovskite devices with high efficiency, brightness and low voltage.

## Introduction

Metal halide perovskites combine facile solution processability, high photoluminescence quantum yields (PLQYs), superior color purity, and high charge carrier mobility, making them promising semiconductors for cost-effective, color-saturated light-emitting diodes (LEDs) capable of operating at high brightness^1–7^. Extensive efforts on materials and device engineering over the past decade have enabled impressive progress in the device performance of single-junction perovskite LEDs (PeLEDs)^1–6^. However, in state-of-the-art devices, both PLQYs of perovskite emitters and device outcoupling efficiencies are now approaching their theoretical limits^5,8^, which increasingly shifts further performance gains towards device-level strategies that can sustain efficiency and lifetime under practical electrical driving conditions.

A natural route to surpass these limits is to integrate two or more perovskite electroluminescence (EL) units in a vertically stacked tandem architecture, a strategy with proven success in established LED technologies^9,10^. Tandem PeLEDs, in which individual subunits are electrically connected in series, enable the integration of multiple perovskite emitters to enhance device performance and offer spectral tunability beyond what single-junction devices can provide^10–13^. More importantly, for a given target radiance, the required current density in tandem PeLEDs consisting of *N* subunits is typically reduced to around 1/*N* of that in a single-junction device. This reduced current-density requirement eases the current load on the driving circuitry, and can substantially prolong operational lifetime^10^.

The performance of tandem devices critically depends on the design of interconnection layers (ICLs), which electrically link the subunits while maintaining favorable optical transparency and minimal electrical overhead^9^. In a series-connected stack, any voltage loss introduced by the ICL adds directly to the device operating voltage and reduces the voltage headroom available for practical driving electronics, making low-loss carrier generation and transport at operational current densities a primary design requirement. Despite their importance, ICLs suitable for tandem PeLEDs remain challenging to realize. Tandem PeLEDs that employed interconnection strategies originally developed for organic and quantum-dot (QD) LEDs exhibit large voltage penalties and/or low brightness under electrical driving^10–13^, highlighting the need for customized ICL designs that simultaneously address interfacial energetics, charge transport, and processing robustness.

Herein, we demonstrate a holistic ICL design for solution-processed tandem PeLEDs that enables low-loss electrical interconnection between vertically stacked perovskite subunits. Using this ICL, we fabricated double-junction PeLEDs featuring a peak EQE of over 40%, a maximum radiance of 690 W sr^−1^ m^−2^, and a turn-on voltage as low as 2.35 V. To further validate the generality and scalability of this strategy, we extended the device architecture to five-junction PeLEDs, which attain peak EQEs of over 60% while retaining consistently low turn-on voltages.

## Results and discussion

**Fig. 1:**
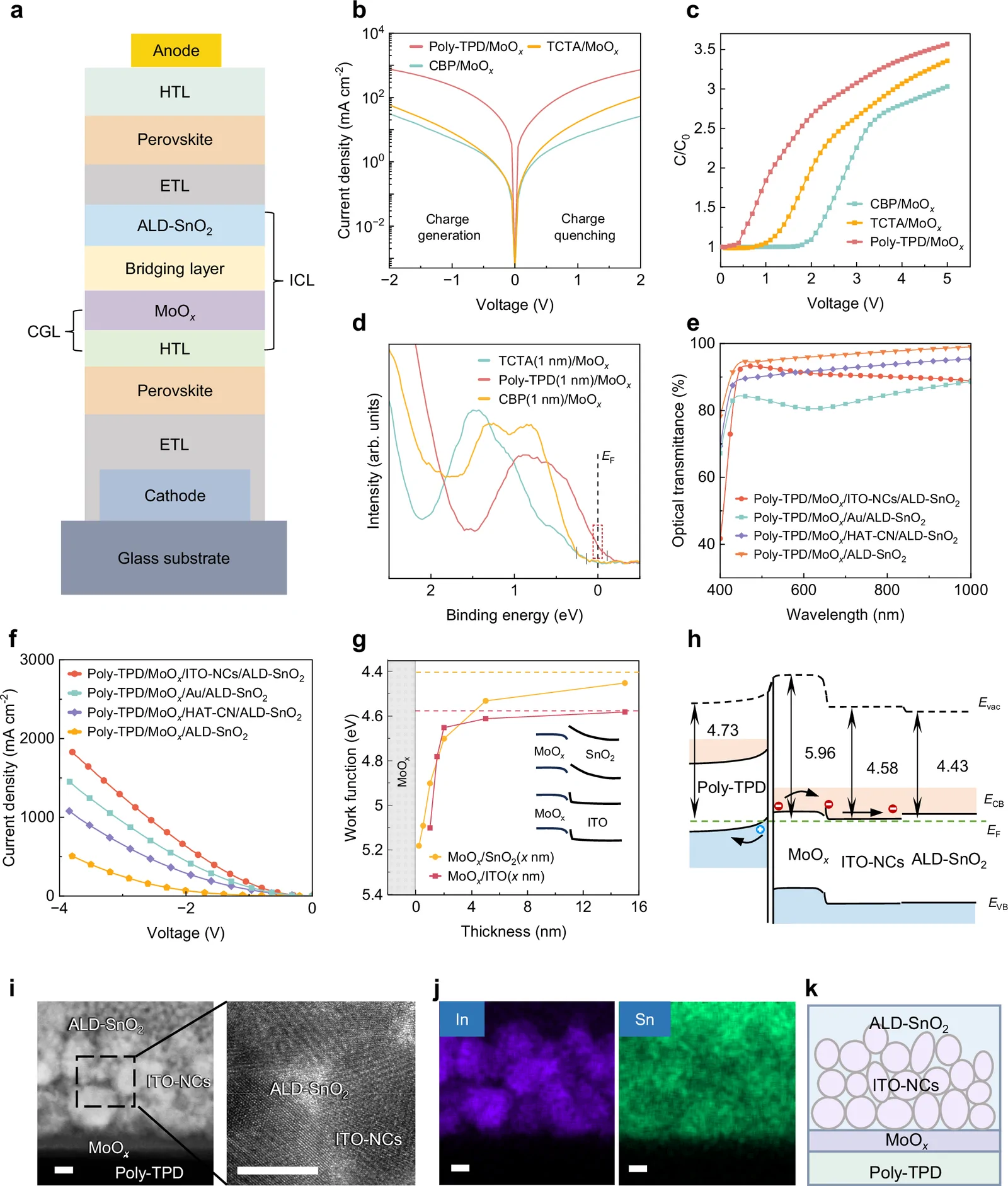
Design and characterization of the composite ICLs for tandem PeLEDs. **a** Schematic diagram of the device architecture of the tandem PeLEDs in this work. **b** *J*–*V* characteristics of the CGL devices with structures of ITO/PEDOT:PSS (10 nm)/HTL (30 nm)/MoO_*x*_ (8 nm)/Au. **c** *C*/*C*_0_-*V* characteristics of the CGL stacks with a structure of ITO/LiF (50 nm)/HTL (30 nm)/MoO_*x*_ (8 nm)/LiF (50 nm)/Al. *C*_0_ is the zero-bias capacitance. **d** UPS spectra of TCTA (1 nm), CBP (1 nm), and Poly-TPD (1 nm) on top of MoO_*x*_. The short markers indicate the HOMO edges of TCTA, CBP, and Poly-TPD, and the dashed line denotes the Fermi level (*E*_F_) edge. **e**, **f** Optical transmittance (**e**) and *J*–*V* characteristics (**f**) of the ICLs of Poly-TPD (30 nm)/MoO_*x*_ (8 nm)/ALD-SnO_2_ (20 nm), and those with the insertion of ITO-NCs (60 nm), Au (1 nm), and HAT-CN (8 nm). The *J*–*V* characteristics (**f**) of the ICLs were measured with a structure of ITO/PEDOT:PSS (10 nm)/ICL/Al. **g** Thickness-dependent work function of ALD-SnO_2_ and ITO-NCs on MoO_*x*_. The dashed lines indicate the work function of bare ALD-SnO_2_ and ITO-NCs, respectively. The inset shows the schematic illustrations of the band bending behavior of MoO_*x*_/ALD-SnO_2_ and MoO_*x*_/ITO-NCs, respectively. **h** Energy level diagram of interfaces of Poly-TPD/MoO_*x*_/ITO-NCs/ALD-SnO_2_, as derived from UPS measurements. **i** Cross-sectional HAADF-STEM (left) and magnified image (right) of the ITO-NCs/ALD-SnO_2_ composite. Scale bar, 10 nm. **j** Elemental mapping images of the In and Sn distributions. Scale bar, 10 nm. **k** Schematic illustration of the hierarchically structured ICL.

As shown in Fig. 1a, the designed ICL for tandem PeLEDs consists of three key components: (i) a charge-generation layer (CGL) ensuring efficient generation of electron-hole pairs at low bias; (ii) a conformally grown compact atomic-layer-deposition processed tin oxide (ALD-SnO_2_) that protects the functional layers underneath and facilitates electron injection toward the upper subunits; and (iii) a conductive bridging layer enabling barrier-free charge transport between CGL and ALD-SnO_2_. By integrating these functions into an optically transparent and mechanically robust composite stack, the ICL mitigates process-induced degradation and enables high-performance tandem operation, including double-junction devices with EQEs above 40% and scalable multijunction integration with EQEs exceeding 60%.

As a key component in tandem LEDs, desired CGLs should generate charge carriers at low bias to minimize the voltage loss in ensuing devices^14^. We designed an effective organic/metal-oxide CGL by stacking molybdenum oxide (MoO_*x*_) with the hole-transport layer (HTL) of poly[N,N′-bis(4-butylphenyl)-N,N′-bis(phenyl)benzidine] (Poly-TPD). Compared with CGLs using HTLs commonly adopted in previous tandem LEDs^10,15^, i.e., 4,4′-bis(carbazol-9-yl)biphenyl (CBP) and tris(4-carbazoyl-9-ylphenyl)amine (TCTA), the superiority of Poly-TPD/MoO_*x*_ is evidenced by nearly ten‑fold higher current densities under the same bias condition (Fig. 1b). Consistently, the capacitance–voltage (*C–V*) measurements reveal a sharp increase in the capacitance at a bias voltage of around 0.4 V for the Poly-TPD/MoO_*x*_ CGL, which is lower than half of that for TCTA/MoO_*x*_ and CBP/MoO_*x*_ (Fig. 1c), indicating a lower threshold voltage for charge generation of the Poly-TPD/MoO_*x*_ CGL.

The energy-band alignment at the CGL interface plays a critical role in determining the charge generation properties^16–20^. While all three types of HTLs show a similar upward band bending at the heterointerfaces (Supplementary Fig. 1), thickness-dependent UPS shows that the Fermi levels (*E*_F_) pinning position at the Poly-TPD/MoO_*x*_ interface lies within ~0.1 eV of Poly-TPD HOMO tail states, whereas it is further away from the HTL HOMO edge for TCTA/MoO_*x*_ and CBP/MoO_*x*_ (around 0.14 and 0.25 eV above the HOMO edge of the HTL, respectively) (Fig. 1d and Supplementary Fig. 2). This indicates a near-degenerate interfacial electronic structure for Poly-TPD/MoO_*x*_, consistent with a high interfacial hole population. This conclusion is also supported by an estimated free carrier concentration of around 10^19 ^cm^−3^ at the interface from *C–V* measurements, which is over an order of magnitude higher than those in TCTA/MoO_*x*_ and CBP/MoO_*x*_ (Supplementary Fig. 3). Temperature-dependent *J–V* results for the Poly-TPD/MoO_*x*_ stack reveal a thermal-emission dominated charge generation process with an interfacial barrier of around 0.2 eV (Supplementary Fig. 4 and Note 1), which is much lower than those previously determined for TCTA/MoO_*x*_ and CBP/MoO_*x*_^21^, further validating the more efficient charge generation at the interface of Poly-TPD/MoO_*x*_ CGL.

After establishing the Poly-TPD/MoO_*x*_ CGL with efficient charge generation, the remaining voltage loss is mainly limited by electron injection and transport from MoO_*x*_ into ALD-SnO_2_. Owing to the large difference in the work function between MoO_*x*_ and ALD-SnO_2_ as well as a low carrier density of ALD-SnO_2_ (~10^17 ^cm^−3^)^22^, a wide space charge region exists at the interface upon the direct contact of MoO_*x*_ with ALD-SnO_2_. To mitigate the charge transport barrier, we designed indium tin oxide nanocrystals (ITO-NCs) as an effective bridging layer to ensure high conductivity of the ICL without sacrificing optical transparency. The resulting ICL exhibits a transmittance exceeding 90% across the wavelength range of 450–1000 nm (Fig. 1e). Compared with other commonly used bridging layers, including thin metals (e.g., Au) and organic molecules (e.g., 1,4,5,8,9,11-hexaazatriphenylenehexacarbonitrile, HAT-CN)^10,12,15^, the ITO-NCs bridging layer delivers markedly higher current density at a given bias while maintaining high optical transparency (Fig. 1e, f) and device performance (as shown further below). The thickness-dependent UPS characterizations reveal a notably narrowed space charge region from over 15 nm at the interfaces of MoO_x_/ALD-SnO_2_ to a value of below 2 nm for MoO_*x*_/ITO-NCs (Fig. 1g and Supplementary Fig. 5). The results are consistent with our simulations and the *C–V*-derived depletion widths of 19.4 nm for MoO_*x*_/ALD-SnO_2_ and 0.7 nm for MoO_*x*_/ITO-NCs/ALD-SnO_2_ (Supplementary Fig. 6).

We emphasize that the ITO-NCs/ALD-SnO_2_ stack also provides enhanced robustness for the ICL. High-resolution cross-sectional scanning transmission electron microscopy-high-angle annular dark field (STEM-HAADF) and element mapping images indicate the conformal growth of a compact ALD-SnO_2_ on the ITO-NCs, forming a hierarchically structured crystalline-amorphous oxide composite within the ICL (Fig. 1i–k). Compared with the organic bridging layers, e.g., HAT-CN, which suffer from irreversible delamination or phase segregation even after mild thermal treatments^23,24^, the ITO-NCs-based ICL could withstand harsh exposure to commonly used solvents (Supplementary Figs. 7 and 8), ensuring damage-free manufacturing of the tandem architectures.

Figure 1h shows the energy-band alignment diagram of the optimal ICL of Poly-TPD/MoO_*x*_/ITO-NCs/ALD-SnO_2_. We conclude that the near-degenerate Poly-TPD/MoO_*x*_ CGL enables efficient charge generation at low bias. With the negligible energy barrier across contact interfaces within the ICL, the holes and electrons can easily transport and inject into the bottom and top subunits for recombination, respectively. For the integral ICL, the voltage for reaching a high current density of 100 mA cm^−2^ is as low as 0.7 V, which is approximately an order of magnitude lower compared with the values in previously demonstrated perovskite-based tandem devices^10,15,25^.

**Fig. 2:**
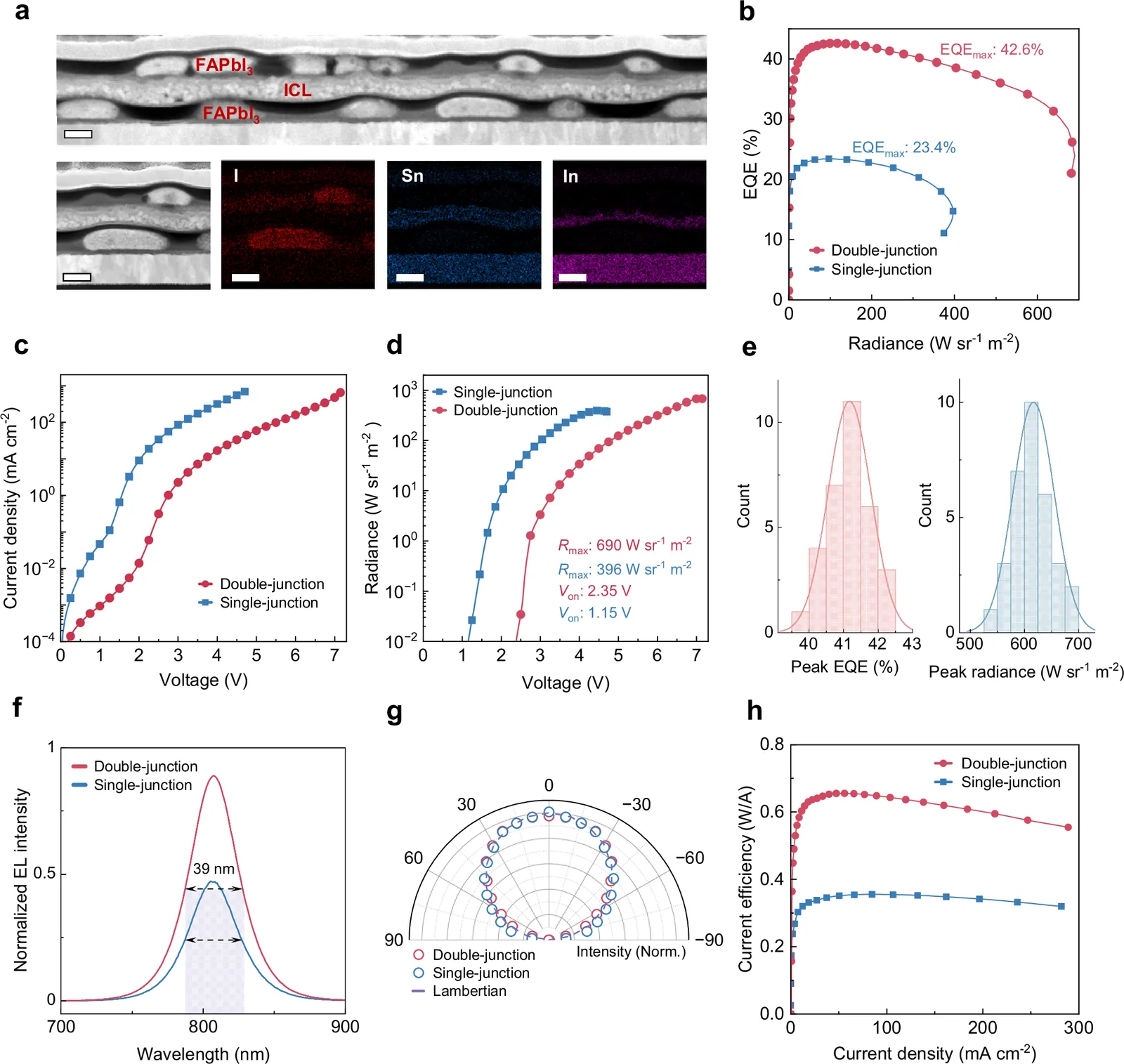
Device architecture and performance of double-junction FAPbI_3_/FAPbI_3_ PeLEDs. **a** Cross-sectional STEM-HAADF and energy-dispersive X-ray spectroscopy (EDS) images of the double-junction FAPbI_3_/FAPbI_3_ PeLEDs. Scale bar, 100 nm. **b**–**d** EQE-*R* (**b**), *J*–*V* (**c**), and *R*–*V* (**d**) characteristics of single-junction FAPbI_3_ and double-junction FAPbI_3_/FAPbI_3_ PeLEDs. The turn-on voltage (*V*_on_) is defined as the voltage required to reach a radiance of 0.01 W sr^−1^ m^−2^. **e** Histograms of the peak EQE and maximum radiance based on 32 double-junction FAPbI_3_/FAPbI_3_ PeLEDs. **f** EL spectra of single- and double-junction devices measured under an identical current density of 100 mA cm^−2^. The shaded area indicates the full width at half maximum (FWHM) of the EL spectra. **g** Normalized angular radiation intensity profiles of the single- and double-junction devices. **h** Current efficiency characteristics of single- and double-junction PeLEDs.

We proceeded to demonstrate the effectiveness of the ICL design in tandem PeLEDs, beginning with devices comprising two identical solution-processed FAPbI_3_ perovskite units. Figure 2a shows the STEM-HAADF images of the FAPbI_3_/FAPbI_3_ tandem device using the optimal ICL of Poly-TPD/MoO_*x*_/ITO-NCs/ALD-SnO_2_, which demonstrates island-structured perovskite domains in two subunits, showing well-defined interfaces between the ICL and perovskite emitters. After optimizing the ICL thickness and subunit fabrication parameters (Supplementary Figs. 9–12), we obtained high-performance FAPbI_3_/FAPbI_3_ tandem devices simultaneously exhibiting a low turn-on voltage (*V*_on_) of 2.35 V, an impressive peak EQE of 42.6%, and a maximum radiance of 690 W sr^−1^ m^−2^ (Fig. 2b–d). We note that both the EQE and radiance values closely match the superposition of the integrated single-junction FAPbI_3_ devices, outperforming literature-reported tandem PeLEDs^10–13^ (Supplementary Table 1) and indicating negligible interconnection loss in our devices. The voltage for reaching a current density of 100 mA cm^−2^ for our double-junction device is around 5.5 V, which is significantly lower than previously reported perovskite-based tandem LEDs^10–13,15,25–27^ (Supplementary Table 1), further validating the superior electrical properties of our ICL. More importantly, our device yields more than two orders of magnitude higher radiance than concurrently reported work at equivalent driving voltages. Performance merit histograms based on 32 devices reveal an average peak EQE and maximum radiance of 41.2% and 616.8 W sr^−1^ m^−2^, respectively (Fig. 2e), demonstrating excellent reproducibility of our tandem PeLEDs utilizing the composite ICL.

Note that although a thick Poly-TPD layer enables a slightly higher EQE of over 45%, the device exhibits severe EQE roll-off under elevated current densities (Supplementary Fig. 9), resulting in a significantly dropped radiance of around 300 W sr^−1^ m^−2^. Concurrently, we also evaluated the device performance of tandem PeLEDs utilizing ICLs consisting of the same Poly-TPD/MoO_*x*_ CGL and ALD-SnO_2_ but adopting the aforementioned bridging designs. Compared to the device employing the optimal ICL consisting of ITO-NCs, all other tandem devices show obviously increased *V*_on_ and severely deteriorated performance merits (Supplementary Fig. 13), underscoring the importance of the synergetic consideration of the optical and electrical properties of the ICL for achieving high-performance tandem PeLEDs.

The double-junction FAPbI_3_/FAPbI_3_ PeLEDs exhibits an identical EL spectrum to its single-junction counterpart but demonstrates nearly doubled emission intensity under the same current density (Fig. 2f). Angular-dependent EL measurement of the tandem PeLED reveals a Lambertian factor of 0.93π (Fig. 2g), a small deviation from the ideal Lambertian distribution (π), which can be attributed to a mildly redirected light extraction arising from the suppressed outcoupling losses from the substrate and waveguide modes, as confirmed by our two-dimensional finite-difference time-domain (FDTD) optical simulations (Supplementary Fig. 14). Benefiting from the effectively combined EL emission of two subunits, which enables halved current density for generating a given radiance and doubled current efficiency (Fig. 2h), the double-junction PeLEDs demonstrate significantly improved operational stability over single-junction devices (Supplementary Fig. 15a, b), highlighting the inherent advantage of the tandem architecture.

**Fig. 3:**
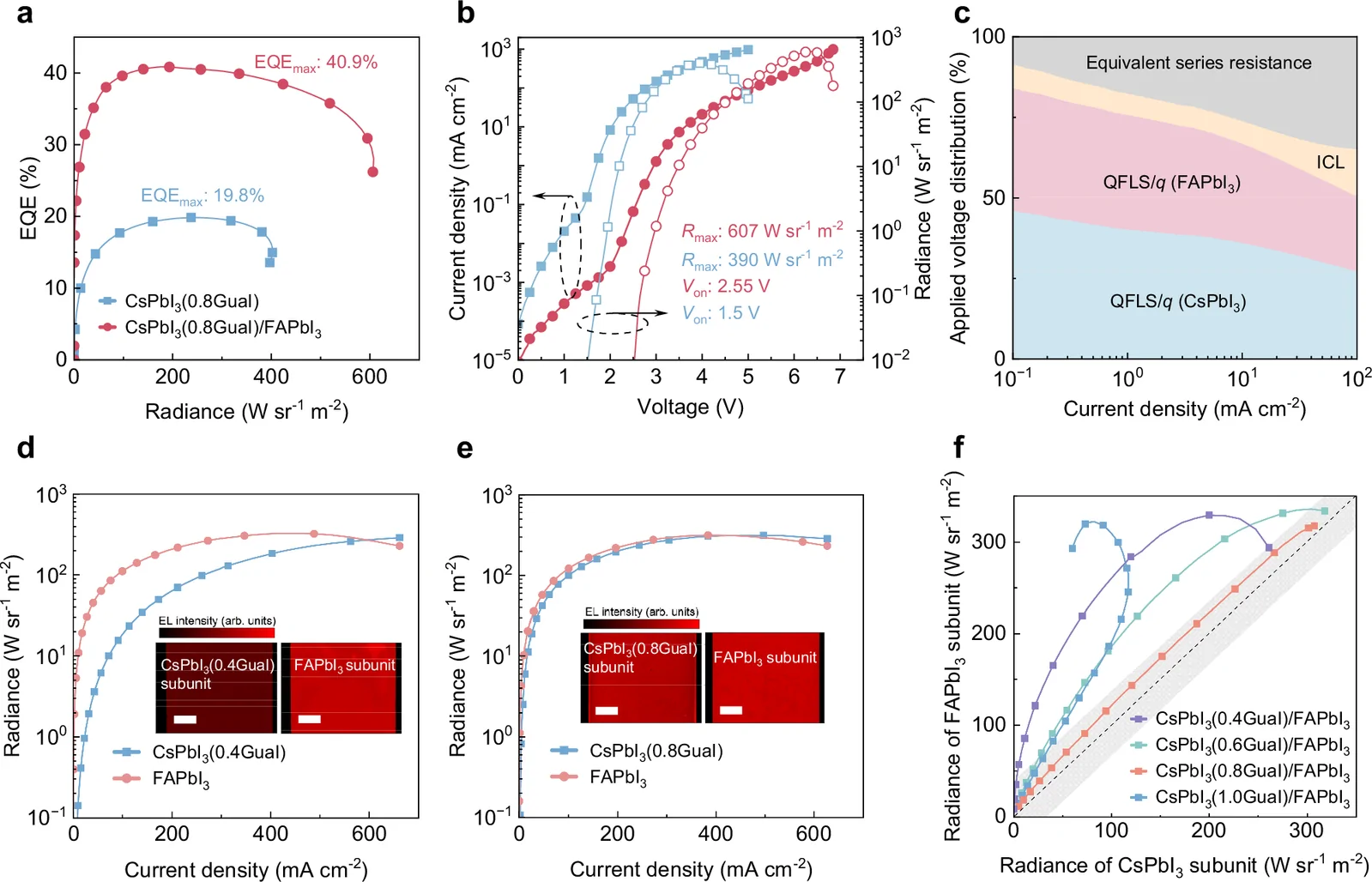
Device performance of double-junction CsPbI_3_(*y*GuaI)/FAPbI_3_ PeLEDs. **a**, **b** EQE-*R* (**a**) and *J*–*V–R* (**b**) characteristics of double-junction CsPbI_3_(0.8GuaI)/FAPbI_3_ PeLED. Characteristics of the single-junction CsPbI_3_(0.8GuaI) device are included for comparison. **c** Reconstructed voltage drop distribution across the perovskite emissive layers and ICL as a function of injection current density. The QFLS divided by the elementary charge (QFLS/q) was used to reflect the voltage drop across the CsPbI_3_ and FAPbI_3_ emissive units for radiative recombination. The voltage drop across the ICL was extracted from Fig. 1f, and the remaining portion of equivalent series resistance was considered as the voltage drop across the other functional layers and interfaces. **d**, **e** *R*–*J* characteristics of the individual subunit extracted from the double-junction CsPbI_3_(0.4GuaI)/FAPbI_3_ (**d**) and CsPbI_3_(0.8GuaI)/FAPbI_3_ (**e**) devices. Insets show EL mappings of the subunits recorded at the current density of 100 mA cm^−2^. Scale bar, 200 μm. **f** Radiance of the bottom CsPbI_3_(*y*GuaI) and the top FAPbI_3_ subunits in operating dual-emission tandem devices. The dashed line indicates an equal radiance from the two subunits during the operation. The shaded region denotes the range in which the radiances between two subunits show a ± 15% difference.

To investigate the applicability of the ICL in connecting perovskite units with different emission wavelengths, we constructed a series of tandem PeLEDs by stacking the FAPbI_3_ on CsPbI_3_ unit processed from precursor solutions containing varied amounts (*y*) of guanidinium iodide (GuaI) additive^28^. The CsPbI_3_(*y*GuaI)/FAPbI_3_ devices exhibit effectively merged EL spectra of the bottom (702 nm) and top (803 nm) units during the whole operation range (Supplementary Fig. 16d–g). The champion device turns on at a low voltage of around 2.55 V, demonstrating a peak EQE of 40.9% and a maximum radiance of 607 W sr^−1^ m^−2^ (Fig. 3a, b), which are close to the sum of the single-junction CsPbI_3_ and FAPbI_3_ devices, along with significantly enhanced operational stability (Supplementary Fig. 15c, d). The quasi-Fermi level splitting (QFLS) analysis and reconstructed voltage profiles (Fig. 3c and Supplementary Fig. 17) reveal well-balanced voltage drop (QFLS/*q*) of the two subunits in the optimal CsPbI_3_(0.8GuaI)/FAPbI_3_ tandem device with the voltage drop over the ICL only accounting for ~10% of the total device configuration. These results not only validate the efficacy of the ICL in connecting perovskite units with different compositions but also provide quantitative information on the interconnection loss.

We notice that there is a significant variation in key performance metrics of the dual-emission tandem PeLEDs depending on the bottom CsPbI_3_ unit (Supplementary Fig. 16a). To uncover the origin of the performance difference, we extracted radiance-current density (*R–J*) curves of the CsPbI_3_ and FAPbI_3_ subunits in tandem devices for detailed analysis. We observe that the radiance from the FAPbI_3_ subunits in all four devices consistently increases and reaches its maximum values at a similar current density of around 400 mA cm^−2^ (Supplementary Fig. 16b–g). In the best-performing tandem device, the CsPbI_3_(0.8GuaI) and FAPbI_3_ units exhibit nearly identical *R–J* characteristics, while obviously distinct radiance characteristics between the two subunits were observed in all other devices with inferior EQEs (Fig. 3d, e and Supplementary Fig. 16b, c). We present the radiance of the top FAPbI_3_ and bottom CsPbI_3_(*y*GuaI) subunits extracted from operating dual-emission tandem PeLEDs in Fig. 3f, which clearly illustrates the EL behaviors of the subunits during the device operation. A larger radiance imbalance inevitably induces additional challenge in stably mixing the emissions from the two perovskite units, notably impairing the overall device performance.

Our ICL can also effectively prevent the problematic halide interdiffusion between the two emissive layers composed of different halides, as evaluated by using tandem PeLEDs with CsPbI_3_ and CsPbBr_3_ as the subunits (Supplementary Fig. 18). During the device operation, both the peak position of the EL from the CsPbI_3_ and CsPbBr_3_ subunits show undetectable changes, suggesting the absence of halide interdiffusion between the two emissive layers under electrical stress (Supplementary Fig. 19c). More detailed EDS cross-section profiles of the degraded device reveal well-confined Br and I accumulation within the top and bottom subunits, respectively (Supplementary Fig. 19a, b). These results confirm the exceptional ion-blocking property of the ICL, enabling further explorations of tandem integrations of perovskite emitters with distinct halide compositions.

**Fig. 4:**
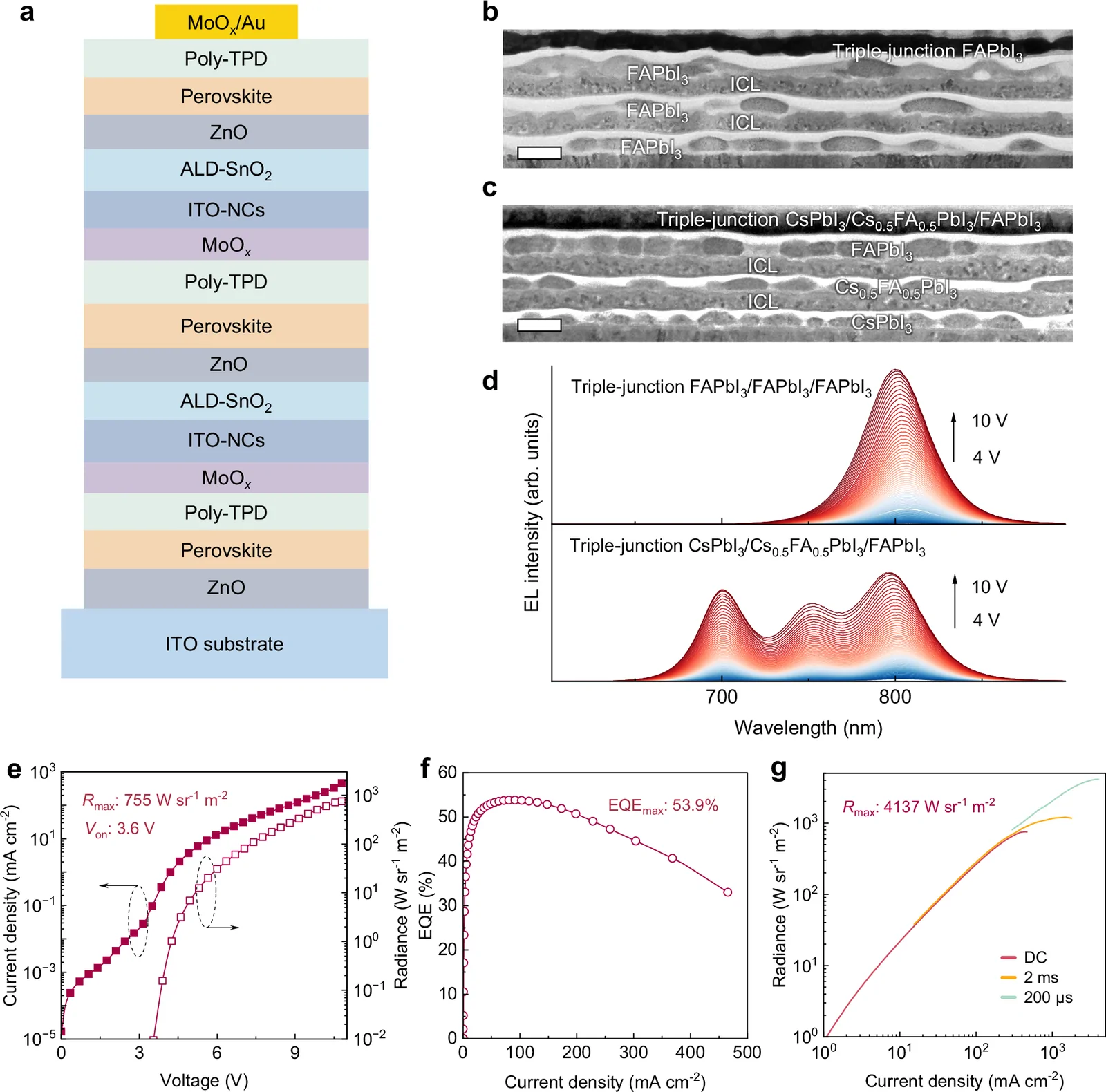
Multijunction PeLEDs. **a** Schematic diagram of the triple-junction tandem PeLED architecture. **b**, **c** Cross-sectional HAADF-STEM images of triple-junction PeLEDs comprising three FAPbI_3_ (**b**) and CsPbI_3_/Cs_0.5_FA_0.5_PbI_3_/FAPbI_3_ units (**c**). Scale bar, 200 nm. **d** EL spectra of the two types of triple-junction PeLEDs. **e**, **f** *J*–*V*–*R* (**e**) and EQE-*J* (**f**) curves of the champion triple-junction FAPbI_3_ device. **g** *R*–*J* curves of a triple-junction FAPbI_3_ device under direct-current (DC) and pulsed operation modes.

We also constructed multijunction PeLEDs as a proof-of-concept for scalable designs comprising perovskite emitters with flexible emission combinations (Fig. 4a–d). The obtained triple-junction FAPbI_3_ and CsPbI_3_/FA_0.5_Cs_0.5_PbI_3_/FAPbI_3_ devices exhibit further enhanced EQEs of 53.9% and 45.7%, respectively (Fig. 4e, f and Supplementary Fig. 20). Notably, the EL emission from the individual subunit with complementary emission in the CsPbI_3_/FA_0.5_Cs_0.5_PbI_3_/FAPbI_3_ device also exhibits a nearly linear increase correlation, corresponding to a stable emission combination during the device operation (Supplementary Fig. 20e). Despite the higher efficiencies, we noticed that the maximum radiances of multijunction PeLEDs are constrained by the enlarged EQE roll-off, mainly due to the more severe Joule heating within operating multijunction tandem PeLEDs compared with that in single-junction device (Supplementary Fig. 21). The thermal characterization shows that the operating temperatures of the four- and five-junction devices are 30–50 °C higher than that of the single-junction device at 400 mA cm^−2^, indicating more pronounced heat accumulation as the junction number increases. Such heat accumulation promotes non-radiative recombination and accelerates EQE roll-off, constrains the maximum radiance of multijunction PeLEDs. By employing commonly used strategies to alleviate the influence of Joule heating, *e.g*., pulsed excitation^29–31^, the triple-junction PeLEDs could operate well under elevated current densities and demonstrate a maximum radiance exceeding 4000 W sr^−1^ m^−2^ (Fig. 4g), outperforming state-of-the-art single-junction devices under the similar current density^32^.

We further demonstrated multijunction PeLEDs comprising four and five FAPbI_3_ units, achieving peak EQEs of 58.2% and 62.5% with turn-on voltages of 5.0 V and 6.6 V, respectively (Supplementary Figs. 22, 23 and Table 1). Notably, despite incorporating more than three interconnection layers in these stacks, the turn-on voltage increases by only ~0.2–0.6 V relative to the expected summed contribution of the added junctions. Across all tandem configurations, the measured turn-on voltages remain below the summed bandgap limit (*∑E*_*g*_*/q*) of the integrated perovskite subunits^33^ (Supplementary Fig. 24 and Table 1). These results directly indicate that the proposed ICL effectively suppresses the interconnection-related voltage penalty, supporting scalable multijunction architectures.

In summary, we have developed a hierarchically structured composite ICL that ensures efficient charge generation at low bias, eliminates interfacial transport barriers, and provides exceptional solvent and thermal robustness while enabling damage-free sequential solution processing of multiple perovskite emitter layers. Using this ICL, we demonstrate double-junction PeLEDs that combine a peak EQE of over 40% (maximum 45.6%), a maximum radiance of 690 W sr^−1^ m^−2^, and a low turn-on voltage of 2.35 V. We further validate the generality and scalability of our approach by extending it to triple-, four-, and five-junction PeLEDs, achieving peak EQEs of 53.9%, 58.2%, and 62.5% with consistently low turn-on voltages. Our results suggest that for multijunction PeLEDs, advanced strategies such as interface engineering or thermal management optimization would be imperative to mitigate performance degradation caused by repetitive thermal annealing and severe Joule heating. We anticipate that the rational low-loss ICL design and strategies on the subunit modulations would boost further developments of emerging tandem PeLEDs for various advanced applications.

## Methods

### Materials

Cesium iodide (CsI, 99.999%), lead bromide (PbBr_2_, 99.999%), cesium bromide (CsBr, 99.999%), ethoxylated polyethyleneimine (PEIE, 80% ethoxylated 37 wt. % in H2O), dimethyl sulfoxide (DMSO, ≥99.9%), N,N-dimethylformamide (DMF, ≥99.8%), chlorobenzene (99.8%), MoO_x_ (99.97%), 4,4’-bis(carbazol-9-yl)biphenyl (CBP, ≥99%), Isopropyl alcohol (IPA, ≥98%), and 4,7,10-trioxa-1,13-tridecanediamine (TTDDA, 97%) were purchased from Sigma-Aldrich. Lead iodide (PbI_2_, 99.999%), tris(4-carbazoyl-9-ylphenyl)amine (TCTA, 99%), and poly[N,N′-bis(4-butylphenyl)-N,N′-bis(phenyl)benzidine] (Poly-TPD, Mw: 15000-25000) were purchased from Xi’an Yuri Solar Co., Ltd. Guanidinium iodide (GuaI, >97%) and 2,2’-[oxybis(ethylenoxy)]diethylamine (ODEA, >97%) were purchased from TCI. Tin (IV) oxide (SnO_2_) colloidal dispersion (15 wt.% in H_2_O) were purchased from Alfa Aesar. Indium tin oxide Nanocrystals (ITO-NCs) dispersion (20–30 nm, 30 wt.% in IPA), 1,4,5,8,9,11-hexaazatriphenylene-hexacarbonitrile (HAT-CN, 99%) and n-butanol ( ≥ 99.4%) were purchased from Macklin. Ethanol (99.5%) and ammonium hydroxide (25–28%) were purchased from Adamas. Formamidinium iodide (FAI, 99.99%) was purchased from Greatcell Solar.

### Preparation of the ammine-hydroxo zinc complex solution

Ammine-hydroxo zinc complex solutions were prepared following previously reported methods^34^. Typically, 7.5 mmol Zn(NO_3_)_2_·6H_2_O was dissolved in distilled water, and the solution was stirred at 30 °C. Subsequently, 25 mmol NaOH in 10 mL water was added dropwise over 10 min. The mixture was centrifuged at 5000 rpm for 5 min to collect the white precipitate, which was washed with 20 mL of water under stirring for 2 min and centrifuged again. This wash cycle was repeated six times. Finally, the precipitate was dispersed in 50 mL ammonia (25–28%) to give the ammine-hydroxo zinc complex solution.

### Preparation of the colloidal solution of ZnO (c-ZnO)

c**-**ZnO nanocrystals were prepared following our previously reported procedure^35^. Briefly, 1.5 mmol Zn(Ac)_2_·2H_2_O in 15 mL DMSO was stirred in a flask until fully dissolved, and then 2.7 mmol TMAH·5H_2_O in 5 mL ethanol was added dropwise over 5 min. The mixture was stirred at 40 °C for 2 h, washed with ethyl acetate (1:1.5 v/v), and centrifuged (3000 rpm, 3 min) to collect the precipitate. The solid was redispersed in 4 mL ethanol and reprecipitated with ethyl acetate (1: 4 v/v), followed by another centrifugation. The final precipitate was dispersed in 8 mL ethanol with 80 µL ethanolamine, filtered through a 0.45 µm PTFE filter, and stored at 8 °C in the dark.

### Preparation of the magnesium-doped ZnO (c-ZnMgO)

c**-**ZnMgO nanocrystals were synthesized following previously reported methods^36^. In brief, 1.35 mmol Zn(Ac)_2_·2H_2_O and 0.15 mmol Mg(Ac)_2_·2H_2_O in 15 mL DMSO were stirred in a flask until fully dissolved, and then 2.25 mmol TMAH·5H_2_O in 5 mL ethanol was added dropwise over 5 min. The mixture was stirred at 25 °C for 1 h, washed with ethyl acetate (1:1.5 v/v), and centrifuged (3000 rpm, 3 min) to collect the precipitate. The solid was redispersed in 2 mL ethanol and reprecipitated with ethyl acetate (1:4 v/v), followed by another centrifugation. The final precipitate was dispersed in 4 mL ethanol, filtered through a 0.45 µm PTFE filter, and stored at 8 °C in the dark.

### Preparation of perovskite precursor solutions

The CsPbI_3_ precursor solution was prepared by dissolving CsI, PbI_2_, and GuaI at a molar ratio of CsI: PbI_2_: GuaI = 1.2: 1: *y* (*y* = 0.4–1) in DMSO. The concentration of PbI_2_ was 0.2 M. The FAPbI_3_ precursor solution was prepared by mixing FAI, PbI_2,_ and ODEA at a molar ratio of FAI: PbI_2_: ODEA = 2:1:0.25 in DMF. The concentration of PbI_2_ was 0.13 M. The FA_0.5_Cs_0.5_PbI_3_ precursor solution was prepared by mixing FAI, CsI, PbI_2,_ and ODEA at a molar ratio of FAI: CsI: PbI_2_: ODEA = 1.9:0.5:1:0.2 in DMF. The concentration of PbI_2_ was 0.14 M. The CsPbBr_3_ perovskite precursor solution was prepared by dissolving CsBr, PbBr_2_, and TTDDA at a molar ratio of CsBr: PbBr_2_: TTDDA = 1.2:1:0.1 in DMSO. The concentration of PbBr_2_ was 0.19 M. All precursor solutions were stirred at 50 °C for 30 min in a glovebox and filtered using a 0.22-μm filter before use.

### Fabrication of single-junction devices

#### Single-junction CsPbI_3_ PeLEDs

Prepatterned ITO glass substrates were cleaned sequentially by ultrasonication in a detergent solution, IPA, and ethanol for 5 min of each step, followed by UV-ozone treatment for 15 min. The SnO_2_ solution (3 wt.% in H_2_O dispersion) was spin-coated onto the substrates at 5000 rpm for 30 s, followed by annealing at 150 °C for 10 min. The alkaline Zn(OH)_2_ was deposited by spin coating the ammine-hydroxo zinc complex solution at 4000 rpm for 30 s, followed by annealing at 60 °C for 10 min in a humidity-controlled glovebox with relative humidity below 30%. Subsequently, the substrates were transferred into a N_2_-filled glovebox. The perovskite layer was deposited from precursor solution at 4000 rpm for 100 s and annealed at 150 °C for 6 min. Poly-TPD was spin-coated at 4000 rpm for 30 s. Finally, MoO_*x*_ (10 nm) and Au (60 nm) were sequentially evaporated as top electrodes by a thermal evaporation system through a shadow mask with an active pixel area of 4.5 mm^2^.

#### Single-junction FAPbI_3_ and FA_0.5_Cs_0.5_PbI_3_ PeLEDs

All processes were carried out in a N_2_-filled glovebox. The c**-**ZnO nanoparticles were spin-coated onto the substrates at 4000 rpm for 30 s, followed by annealing at 150 °C for 10 min. A layer of PEIE (1.1 mg ml^−1^ in IPA) was then spin-coated onto c-ZnO at 5000 rpm for 30 s, followed by 100 °C for 10 min. The perovskite layer was deposited from FAPbI_3_ and FA_0.5_Cs_0.5_PbI_3_ precursor solutions at 3000 rpm for 30 s and then annealed at 100 °C for 10 min. Poly-TPD (12 mg ml^−1^) was spin-coated at 4000 rpm for 30 s. Finally, MoO_*x*_ (10 nm) and Au (60 nm) were sequentially evaporated as top electrodes through a shadow mask with an active pixel area of 4.5 mm^2^.

#### Double-junction PeLEDs

For the CsPbI_3_/FAPbI_3_ and FAPbI_3_/FAPbI_3_ devices, the bottom CsPbI_3_ and FAPbI_3_ units, including the electron-transporting layer (ETL) and perovskite emissive layers, were prepared using the same processing steps as the corresponding single-junction device, as mentioned above. After the fabrication of the perovskite film, Poly-TPD was deposited from the chlorobenzene solution (14 mg ml^−1^) at 3000 rpm for 30 s. Subsequently, MoO_*x*_ was thermally evaporated using a thermal evaporation system at a pressure of 2 × 10^−4 ^Pa. After that, ITO-NCs dispersion (5 wt.% in n-butanol) was deposited onto the MoO_*x*_ film at 5000 rpm for 30 s, followed by annealing at 80 °C for 2 min. Next, the samples were transferred to the ALD system to deposit SnO_2_ at 80 °C for 30 min (chamber pressure: 0.1 Torr; precursors: tetrakis(dimethylamido)tin(IV) (TDMASn) and deionized water, with one deposition cycle consisting of TDMASn pulse (0.18 s)–N_2_ purge (5 s)–H_2_O pulse (0.03 s)–N₂ purge (5 s). A total of 170 cycles is used to achieve a thickness of 20 nm). The c-ZnO solution was spin-coated onto the ALD-SnO_2_ at 4000 rpm for 30 s and annealed at 100 °C for 2 min. PEIE was then spin-coated onto ZnO at 5000 rpm for 30 s, followed by annealing at 100 °C for 2 min. The FAPbI_3_ film of the top perovskite unit was deposited at 3000 rpm for 30 s and annealed at 100 °C for 7 min. Poly-TPD (15 mg ml^−1^) was spin-coated at 3000 rpm for 30 s. Finally, MoO_*x*_ (10 nm) and Au (60 nm) were sequentially evaporated as top electrodes through a shadow mask with an active pixel area of 4.5 mm^2^. The single-junction CsPbI_3_ unit and ICL of double-junction CsPbI_3_/CsPbBr_3_ PeLEDs were prepared using the same processing steps as described above. After that, the ZnMgO solution was spin-coated at 4000 rpm for 30 s and annealed at 100 °C for 2 min. The CsPbBr_3_ film was prepared by spin-coating the precursor solution at 4000 rpm for 30 s and annealed at 100 °C for 2 min. The following deposition was kept the same as other double-junction devices.

#### Triple-junction CsPbI_3_/FA_0.5_Cs_0.5_PbI_3_/FAPbI_3_, triple-, four-, and five-junction FAPbI_3_ PeLEDs

All the multijunction (triple-, four-, and five-junction) devices were constructed by employing the same ICL design with all their subunits fabricated following the fabrication process as described above.

### Device characterizations

The *J*–*V* characteristic curves, EL spectra, radiance and EQE of LEDs were measured by using a computer-controlled system at room temperature within an N_2_-filled glovebox. This system comprises a Keithley 2400 source meter, a fiber-optic integrating sphere (FOIS-1), and a QE Pro spectrometer (Ocean Optics). This entire setup was calibrated by a standard vis-NIR light source (HL-3P-INT-CAL plus, Ocean Optics) and cross-checked between research groups at the University of Electronic Science and Technology of China and Linköping University. The device operational lifetime was measured using a 32-channel LED lifetime test system (D3000-32, CRYSCO) in an N_2_-filled glovebox. Capacitance–voltage measurements were performed using a Keithley 4200 parameter analyzer with an oscillation level of 50 mV and a frequency of 100 kHz. The angular dependence of emission intensity was measured using a Phelos angle-dependent spectrometer. For PeLEDs measurement in pulsed voltage mode, square voltage pulses with 200 μs or 2 ms width at a repetition rate of 50 Hz were generated by the Keithley 2602B and applied to the device. The emitted optical power from the devices was calibrated with an integrating sphere (INS50, Labsphere) coupled with a high-speed Si PIN photodiode (S5971, HAMAMATSU).

### Materials and film characterizations

Scanning electron microscopy (SEM) imaging was carried out by the GenimiSEM300 system using a 5 kV acceleration voltage. The cross-sectional samples were prepared by using a dual-beam focused-ion-beam (FIB) system (ZEISS Crossbeam 540). A TEM system (FEI Talos F200X G2) was used to obtain the scanning transmission electron microscopy-high-angle annular dark field (STEM-HAADF), TEM, and energy-dispersive X-ray spectroscopy (EDS) images of the samples. In situ PL spectra were measured using a 405 nm laser and PG2000 Pro spectrometer (Shanghai Ideaoptics Co., Ltd.) at room temperature in a N_2_-filled glovebox. The X-ray photoelectron spectroscopy (XPS) and ultraviolet photoelectron spectroscopy (UPS) measurements were conducted by the Axis Supra system. This system is equipped with a monochromatic Al Kα X-ray source for XPS. The UPS resolution was determined to be 120 meV at a pass energy of 10 eV using the Fermi edge of the valence band for gold. The thickness of the spin-coated Poly-TPD layer in UPS and XPS measurements was determined by spectroscopic ellipsometry (J.A. Woollam RC2) by fitting the data using the Cauchy model.

### Simulation of energy-level band alignment

The energy-level band alignment and carrier distribution were simulated based on the two-dimensional model using commercial simulation software (APSYS, Crosslight Software Inc.). The energy band parameters of each layer of ICL were determined from UPS measurement (Supplementary Fig. 25). Structural dimensions were configured to match experimental architecture: Poly-TPD (40 nm), MoO_*x*_ (8 nm), ITO-NCs (30 nm), and SnO_2_ (20 nm). Notably, the Poly-TPD layer was modeled as a conventional inorganic semiconductor, omitting interfacial interactions with MoO_*x*_.

### Temperature-dependent electrical measurements of the CGL stack

Temperature-dependent transport property measurements were conducted using a Keithley 4200A-SCS Parameter Analyzer. The sample was cooled using a vacuum closed-cycle cryostat system comprising a 4-K refrigerator (Sumitomo Heavy Industries Ltd.) and an exchange helium cooling system (Janis) controlled by a temperature controller (Model 22 C, Cryogenic Control System Inc.). The CGL stack was mounted on a metallic cold plate inside a vacuum chamber thermally connected to the helium vapor chamber.

### Optical and thermal simulations

The optical behavior of the tandem PeLEDs was analyzed using classical electromagnetic simulations. Light emission was modeled by placing unit electric dipoles at the center of the perovskite emissive layers, representing radiative decay from molecular emitters. An isotropic dipole orientation was assumed, with a 2:1 ratio of horizontal to vertical dipoles, consistent with typical orientation distributions in perovskite thin films. To simulate light propagation within the multilayer architecture, finite-difference time-domain (FDTD) calculations were performed. Perfectly matched layers (PMLs) were applied at all boundaries except the top Au electrode to suppress artificial reflections. Scattering boundary conditions were implemented beyond the PML domain. All constituent layers were treated as isotropic, and their wavelength-dependent refractive indices were adopted from experimental data (see Supplementary Fig. 26). Photon reabsorption and recycling effects were incorporated into the model to more accurately reflect internal optical dynamics. Spatial profiles of the electromagnetic fields and power dissipation were extracted to assess optical confinement, loss channels, and light outcoupling efficiency. For the thermal simulation of the devices, a two-dimensional electro-thermal model was established in COMSOL Multiphysics 5.6 using the Electromagnetic Heating Multiphysics Module to analyze the heat generation in single-junction and tandem PeLEDs. The model self-consistently solved the coupled electromagnetic and heat transfer equations. Joule heating from current flow and heat from non-radiative recombination were included as volumetric sources.

### EL mapping measurements

EL images were captured using a CMOS camera (Daheng Imaging ME2P-1230-9GM-P, 4096 × 3000 pixels) mounted on a microscope within a light-tight enclosure. The images were recorded at room temperature (25 °C) with an exposure time of 50 μs, using a Keithley 2400 source meter to supply current. A 750-nm short-pass filter and a 750-nm long-pass filter were applied to isolate specific emissions from the CsPbI_3_ and FAPbI_3_ unit, respectively. Background noise was subtracted from all images to ensure accurate data analysis.

### Quasi-Fermi level splitting of emissive layers

The absolute EL spectra of the device were obtained by measuring the emitted photon flux from the two emissive layers in all directions based on the EL setup mentioned above. The evaluation of photon emission from the semiconductor materials was based on the generalized Planck’s law^37^,

1 $${\phi }_{em}(\hslash \omega )=a(\hslash \omega )\frac{\varOmega }{4{\pi }^{3}{\hslash }^{3}{c}^{2}}\frac{{(\hslash \omega )}^{2}}{\exp (\frac{\hslash \omega -\varDelta \mu }{{k}_{{{{\rm{B}}}}}T})-1}$$

where ${\phi }_{em}$ represents the spectral photon flux, Ω denotes the solid angle, ℏ*ω* is the photon energy, *a*(ℏ*ω*) accounts for the absorptance of the emissive layer, ℏ is the reduced Planck’s constant, *c* is the speed of light in a vacuum, *k*_B_ is the Boltzmann constant and Δ*μ* is the QFLS. The Boltzmann approximation was deemed applicable due to the satisfaction of the condition ℏ*ω*−Δ*μ*≫*k*_*B*_*T* across all injection densities and temperatures. The absorptance of corresponding perovskite emitters is shown in Supplementary Fig. 27. The QFLS can be extracted from the absolute EL spectra based on a fit of the high-energy slope of the emission peaks to the above equation.

## Supplementary information

Supplementary Information

Transparent Peer Review file

## Source data

Source data

## Data Availability

The authors declare that the main data supporting the findings of this study are available within the article and its supplementary information files, and all data are available from the corresponding authors upon request. Source data are provided with this paper.

## References

[1] Song, Y.-H. et al. Intragrain 3D perovskite heterostructure for high-performance pure-red perovskite LEDs. *Nature* **641**, 352–357 (2025). 40335712 10.1038/s41586-025-08867-6

[2] Lian, Y. et al. Downscaling micro- and nano-perovskite LEDs. *Nature* **640**, 62–68 (2025). 40108467 10.1038/s41586-025-08685-w

[3] Wei, K. et al. Perovskite heteroepitaxy for high-efficiency and stable pure-red LEDs. *Nature* **638**, 949–956 (2025). 39972133 10.1038/s41586-024-08503-9

[4] Xiong, W. et al. Controllable p- and n-type behaviours in emissive perovskite semiconductors. *Nature* **633**, 344–350 (2024). 39261614 10.1038/s41586-024-07792-4

[5] Li, M. et al. Acceleration of radiative recombination for efficient perovskite LEDs. *Nature* **630**, 631–635 (2024). 38811739 10.1038/s41586-024-07460-7 PMC11186751

[6] Kim, J. S. et al. Ultra-bright, efficient and stable perovskite light-emitting diodes. *Nature* **611**, 688–694 (2022). 36352223 10.1038/s41586-022-05304-w

[7] Liu, X. K. et al. Metal halide perovskites for light-emitting diodes. *Nat. Mater.* **20**, 10–21 (2021). 32929252 10.1038/s41563-020-0784-7

[8] Baek, S. et al. Grain engineering for efficient near-infrared perovskite light-emitting diodes. *Nat. Commun.* **15**, 10760 (2024). 39737972 10.1038/s41467-024-55075-3 PMC11685452

[9] Fung, M. K., Li, Y. Q. & Liao, L. S. Tandem organic light-emitting diodes. *Adv. Mater.* **28**, 10381–10408 (2016). 27735102 10.1002/adma.201601737

[10] Ke, Y. et al. High-performance tandem perovskite LEDs through interlayer photon recycling. *Nature* **649**, 53–58 (2026). 41219499 10.1038/s41586-025-09865-4

[11] Mao, J. et al. All-perovskite emission architecture for white light-emitting diodes. *ACS Nano* **12**, 10486–10492 (2018). 30222315 10.1021/acsnano.8b06196

[12] Yan, Y. et al. High-efficiency tandem white perovskite light-emitting diodes by using an organic/inorganic intermediate connector. *Crystals* **12**, 1286 (2022).

[13] Wang, R. et al. Minimizing energy barrier in intermediate connection layer for monolithic tandem WPeLEDs with wide color gamut. *Adv. Funct. Mater.* **33**, 2215189 (2023).

[14] Xie, Y., Liao, L. & Fung, M. Hybrid design of light-emitting diodes in tandem structures. *Adv. Funct. Mater.* **2401789**, 1–16 (2024).

[15] Kong, L. et al. Efficient and stable hybrid perovskite-organic light-emitting diodes with external quantum efficiency exceeding 40 per cent. *Light: Sci. Appl.* **13**, 138 (2024). 38866757 10.1038/s41377-024-01500-7 PMC11169476

[16] Sun, H. et al. High efficiency tandem organic light emitting diode using an organic heterojunction as the charge generation layer: an investigation into the charge generation model and device performance. *ACS Photonics* **2**, 271–279 (2015).

[17] Meyer, J. et al. Charge generation layers comprising transition metal-oxide/organic interfaces: electronic structure and charge generation mechanism. *Appl. Phys. Lett.* **96**, 1–4 (2010).

[18] Lei, Y. et al. Solution-processed conducting polymer/metal oxide charge generation layer: preparation, electrical properties, and charge generation mechanism. *J. Phys. Chem. C.* **121**, 793–800 (2017).

[19] Peng, J. et al. Charge-generation structures and their applications in light-emitting devices. *J. Phys. D Appl. Phys.* **57**, 333001 (2024).

[20] Guo, Q. et al. Charge generation mechanism of tandem organic light emitting diodes with pentacene/C70 organic heterojunction as the connecting layer. *J. Mater. Chem. C* **4**, 376–382 (2016).

[21] Kotadiya, N. B. et al. Universal strategy for Ohmic hole injection into organic semiconductors with high ionization energies. *Nat. Mater.* **17**, 329–334 (2018). 29459747 10.1038/s41563-018-0022-8

[22] Yu, Z. et al. Simplified interconnection structure based on C60/SnO2-x for all-perovskite tandem solar cells. *Nat. Energy* **5**, 657–665 (2020).

[23] Fenter, P., Schreiber, F., Bulović, V. & Forrest, S. R. Thermally induced failure mechanisms of organic light emitting device structures probed by X-ray specular reflectivity. *Chem. Phys. Lett.* **277**, 521–526 (1997).

[24] Burke, S. A., Topple, J. M. & Grütter, P. Molecular dewetting on insulators. *J. Phys. Condens. Matter* **21**, 423101 (2009). 21715835 10.1088/0953-8984/21/42/423101

[25] Lee, H.-D. et al. Valley-centre tandem perovskite light-emitting diodes. *Nat. Nanotechnol.* **19**, 624–631 (2024). 38228805 10.1038/s41565-023-01581-2

[26] Sun, S. et al. Highly efficient hybrid perovskite/organic tandem white light emitting-diodes with external quantum efficiency exceeding 20%. *Adv. Funct. Mater.* **33**, 1–8 (2023).

[27] Zhu, M. et al. Perovskite/organic hybrid tandem light-emitting diodes with an external quantum efficiency of over 40%. *J. Mater. Chem. C.* **12**, 2623–2628 (2024).

[28] Zeng, J. et al. Switchable interfacial reaction enables bright and stable deep-red perovskite light-emitting diodes. *Nat. Photonics* **18**, 325–333 (2024).

[29] Zhao, L. et al. Thermal properties of polymer hole-transport layers influence the efficiency roll-off and stability of perovskite light-emitting diodes. *Nano Lett.* **23**, 4785–4792 (2023). 37220025 10.1021/acs.nanolett.3c00148

[30] Zou, C. et al. Electrically driven lasing from a dual-cavity perovskite device. *Nature* **645**, 369–374 (2025). 40866697 10.1038/s41586-025-09457-2 PMC12422965

[31] Sun, Y. et al. Bright and stable perovskite light-emitting diodes in the near-infrared range. *Nature* **615**, 830–835 (2023). 36922588 10.1038/s41586-023-05792-4

[32] Li, Z. et al. Highly bright perovskite light-emitting diodes enabled by retarded Auger recombination. *Nat. Commun.* **16**, 927 (2025). 39843419 10.1038/s41467-025-56001-x PMC11754759

[33] Lian, Y. et al. Ultralow-voltage operation of light-emitting diodes. *Nat. Commun.* **13**, 3845 (2022). 35788132 10.1038/s41467-022-31478-y PMC9253117

[34] Meyers, S. T. et al. Aqueous inorganic inks for low-temperature fabrication of ZnO TFTs. *J. Am. Chem. Soc.* **130**, 17603–17609 (2008). 19053193 10.1021/ja808243k

[35] Kuang, C. et al. Critical role of additive-induced molecular interaction on the operational stability of perovskite light-emitting diodes. *Joule* **5**, 618–630 (2021).

[36] Deng, Y. et al. Solution-processed green and blue quantum-dot light-emitting diodes with eliminated charge leakage. *Nat. Photonics* **16**, 505–511 (2022).

[37] Wurfel, P. The chemical potential of radiation. *J. Phys. C Solid State Phys.* **15**, 3967–3985 (1982).

