# Supplementary material for: High-performance multijunction perovskite LEDs with reduced interconnection loss
Source: Nat Commun. 2026 Jul 17;17:8781. doi: 10.1038/s41467-026-75756-5 (PMC13493991; doi:10.1038/s41467-026-75756-5)
Supplement: Supplementary file 1 [file 41467_2026_75756_MOESM1_ESM.pdf]

Supplementary Information for

High-performance multijunction perovskite LEDs with reduced interconnection loss

Feiyun Zhao[1,2,†], Haifeng Zhao[1,3,†], Zhennan Tian[4], Aobo Ren[1*], Lechuan Chen[1], Hengyu Liu[1], Jun Wu[1], Kai Shen[1], Wuyang Ren[1], Chuang Li[1], Chunyang Yin[1], Junsheng Yu[4,5], Feng Gao[6*], Sai Bai[1,5*], Jiang Wu[1,5*], Yanrong Li[5,7]

[1]Institute of Fundamental and Frontier Sciences, University of Electronic Science and Technology of China, Chengdu 611731, China

[2]Southwest Institute of Technical Physics, Chengdu 610041, China

[3]Yibin Institute of UESTC, University of Electronic Science and Technology of China (UESTC), Yibin 644005, China

[4]School of Optoelectronic Science and Engineering, University of Electronic Science and Technology of China, Chengdu 611731, China

[5]State Key Laboratory of Electronic Thin Films and Integrated Devices, University of Electronic Science and Technology of China, Chengdu 611731, China

[6]Department of Physics, Chemistry and Biology, Linköping University, Linköping 58183, Sweden

[7]Northwestern Polytechnical University, Xi'an 710072, China

[†]These authors contributed equally: Feiyun Zhao, Haifeng Zhao

[*]Corresponding authors: Aobo Ren (aobo.ren@uestc.edu.cn), Feng Gao (feng.gao@liu.se), Sai Bai (sai.bai@uestc.edu.cn), Jiang Wu (jiangwu@uestc.edu.cn)

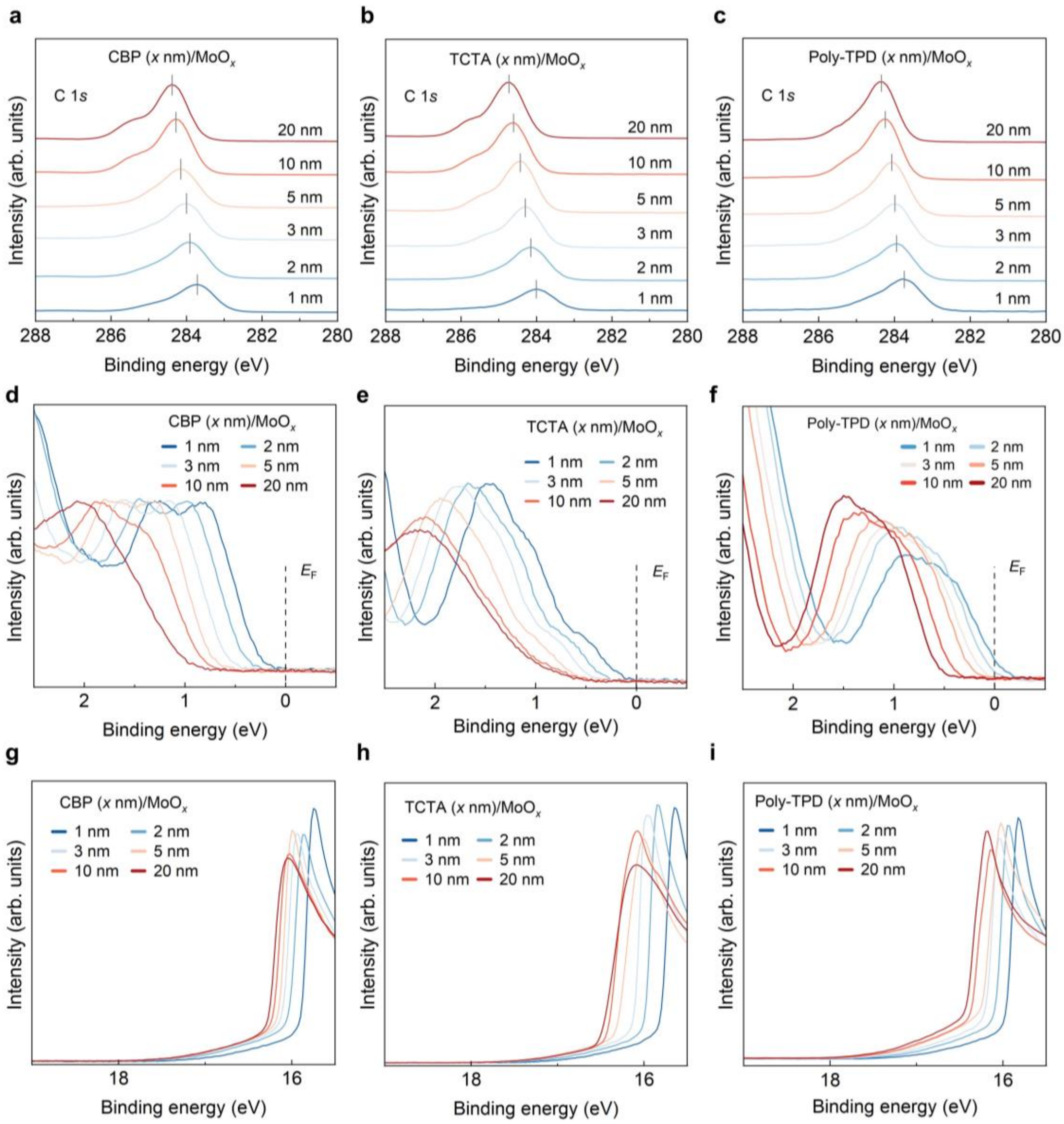

Supplementary Fig. 1 | Thickness-dependent XPS and UPS spectra of different CGL stacks. a-c, C 1*s* XPS spectra. **d-i**, UPS spectra. XPS spectra show that the C 1*s* peak shifts toward higher binding energy with increasing HTL thickness, indicating upward band bending of the HTL at the heterointerface.

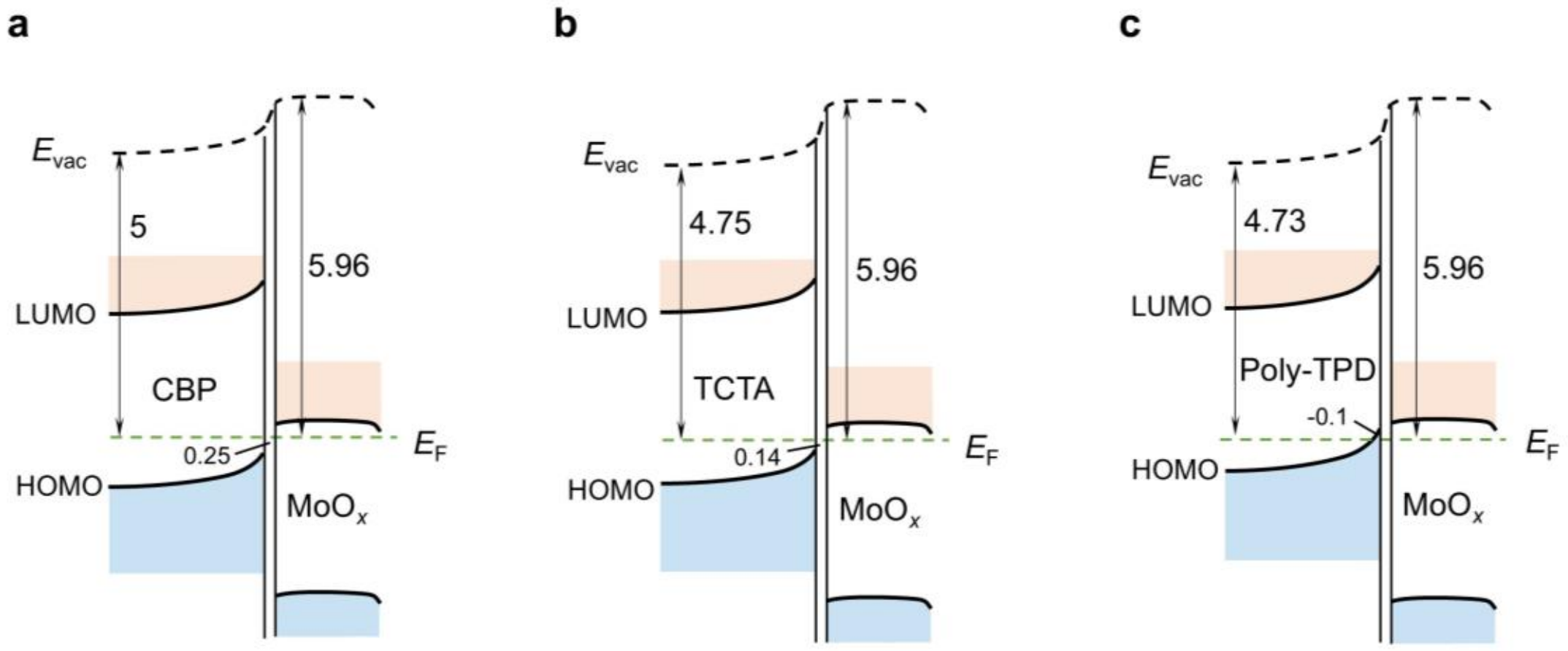

Supplementary Fig. 2 | Schematic energy-level alignment diagram of different CGL stacks. a, CBP/MoO$_x$. **b,** TCTA/MoO$_x$. **c,** Poly-TPD/MoO$_x$. The Fermi level (E_F) pinning positions were extracted from the UPS measurement results.

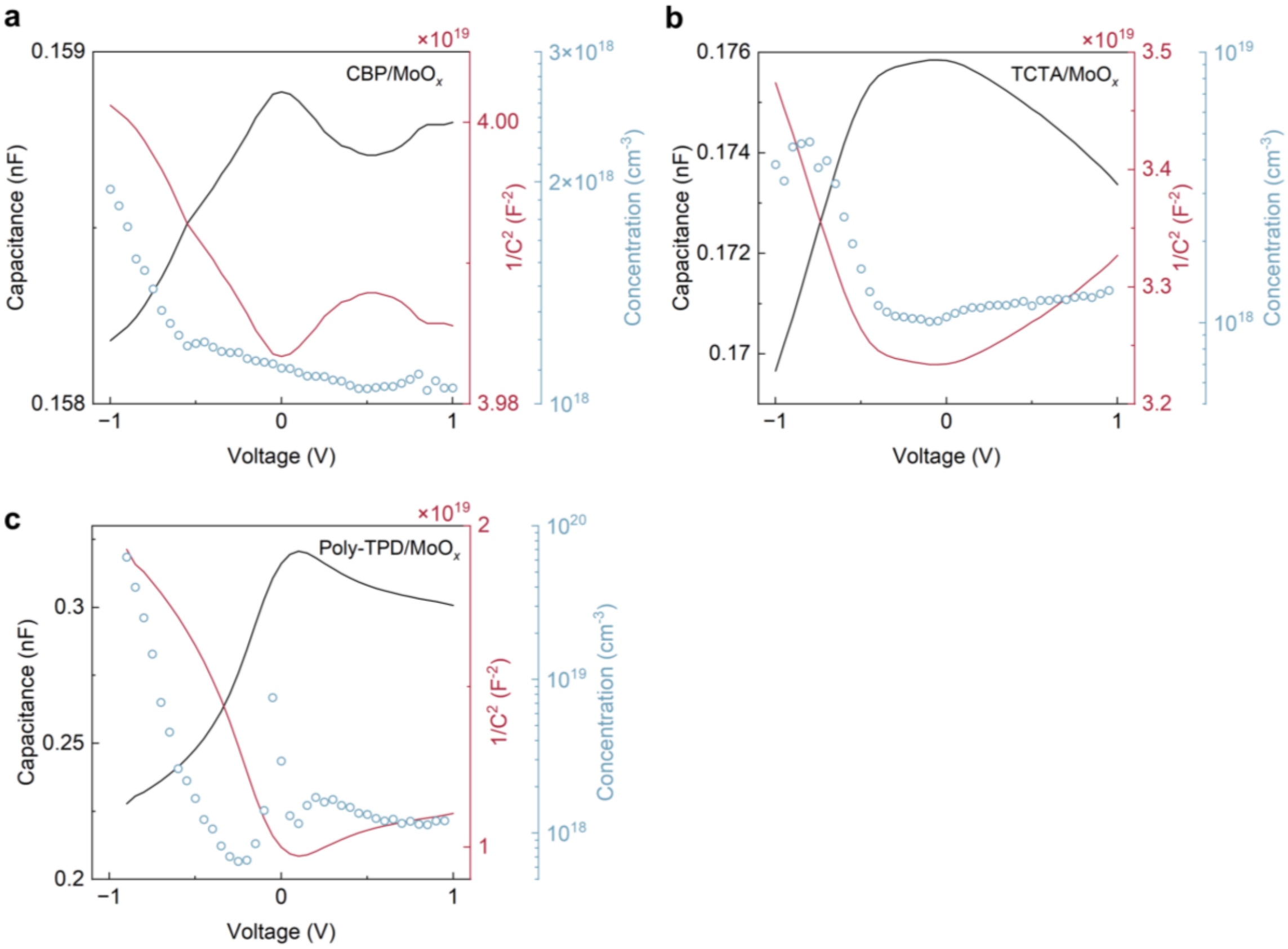

Supplementary Fig. 3 | Characteristics of *C*, C^{-2}, and free carrier concentration versus voltage for different CGLs. a, CBP/MoO_x. **b,** TCTA/MoO_x. **c,** Poly-TPD/MoO_x. The free carrier concentration was calculated based on the Mott-Shockley equation[1,2]. The free carrier concentration can be obtained by fitting the slope of the $1/C^2$-V curve (Supplementary Note 2). The capacity-voltage characterizations of CGL were measured with a structure of ITO/HTL (30 nm)/MoO_x (8 nm)/Au.

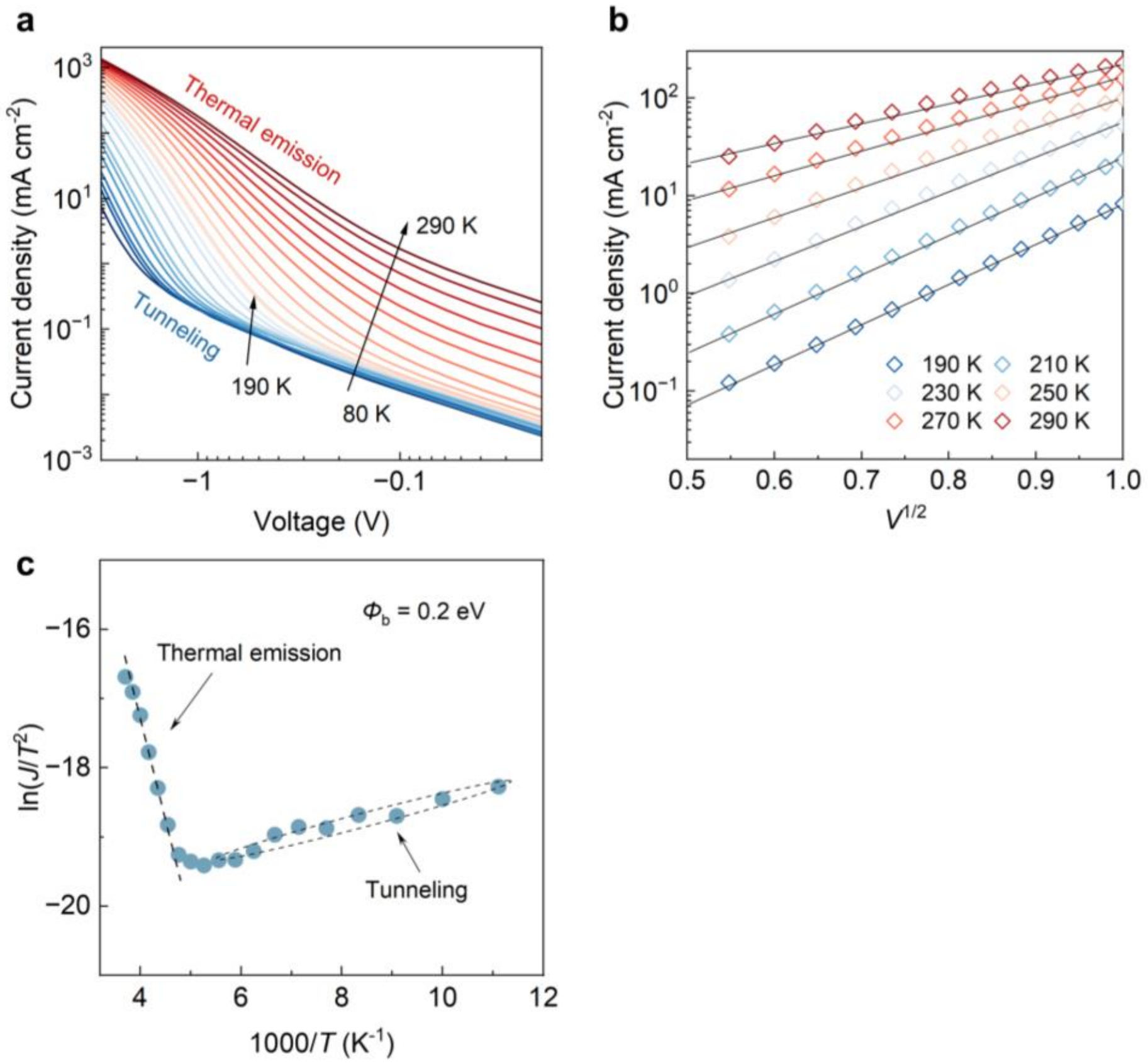

Supplementary Fig. 4 | Temperature-dependent electrical characteristics of the Poly-TPD/MoO$_x$ CGL. a, Temperature-dependent J-V characteristics. The current density exhibits pronounced thermal dependence above 190 K, indicating a transition from tunneling to thermally activated charge transport. **b**, Temperature-dependent ln(J)-$V^{1/2}$ characteristics of the CGL. The fitted ln(J)-$V^{1/2}$ curves exhibit a linear trend with a positive slope, which correlates well with the Richardson-Schottky (R-S) thermal emission model[3]. **c**, R-S fitting of the CGL device at $V = 0$. The corresponding barrier heights were extracted from the slope of the dotted line with the R-S model.

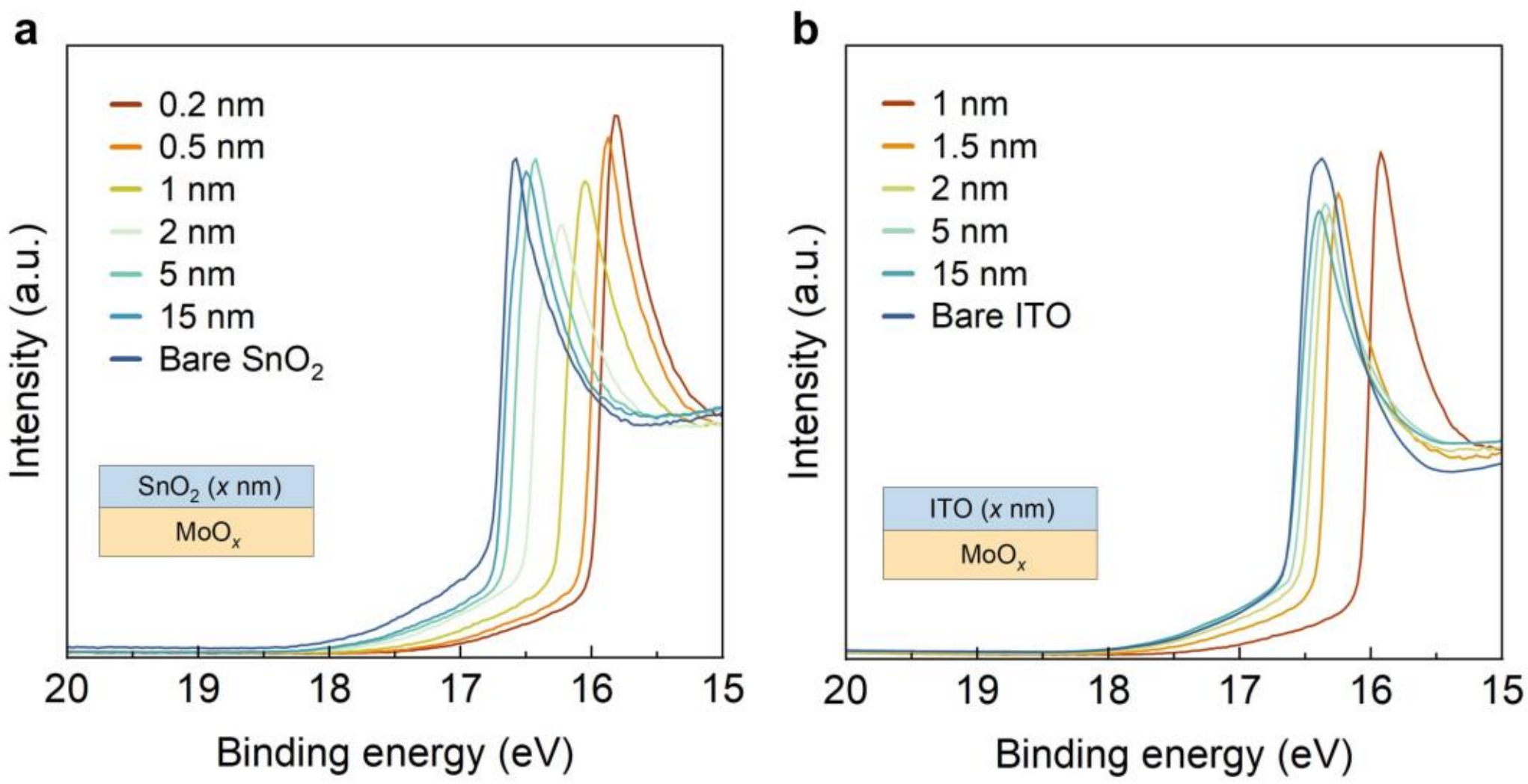

Supplementary Fig. 5 | Thickness-dependent UPS spectra. a, ALD-SnO_2 (*x* nm)/MoO_x. **b**, ITO-NCs (*x* nm)/MoO_x.

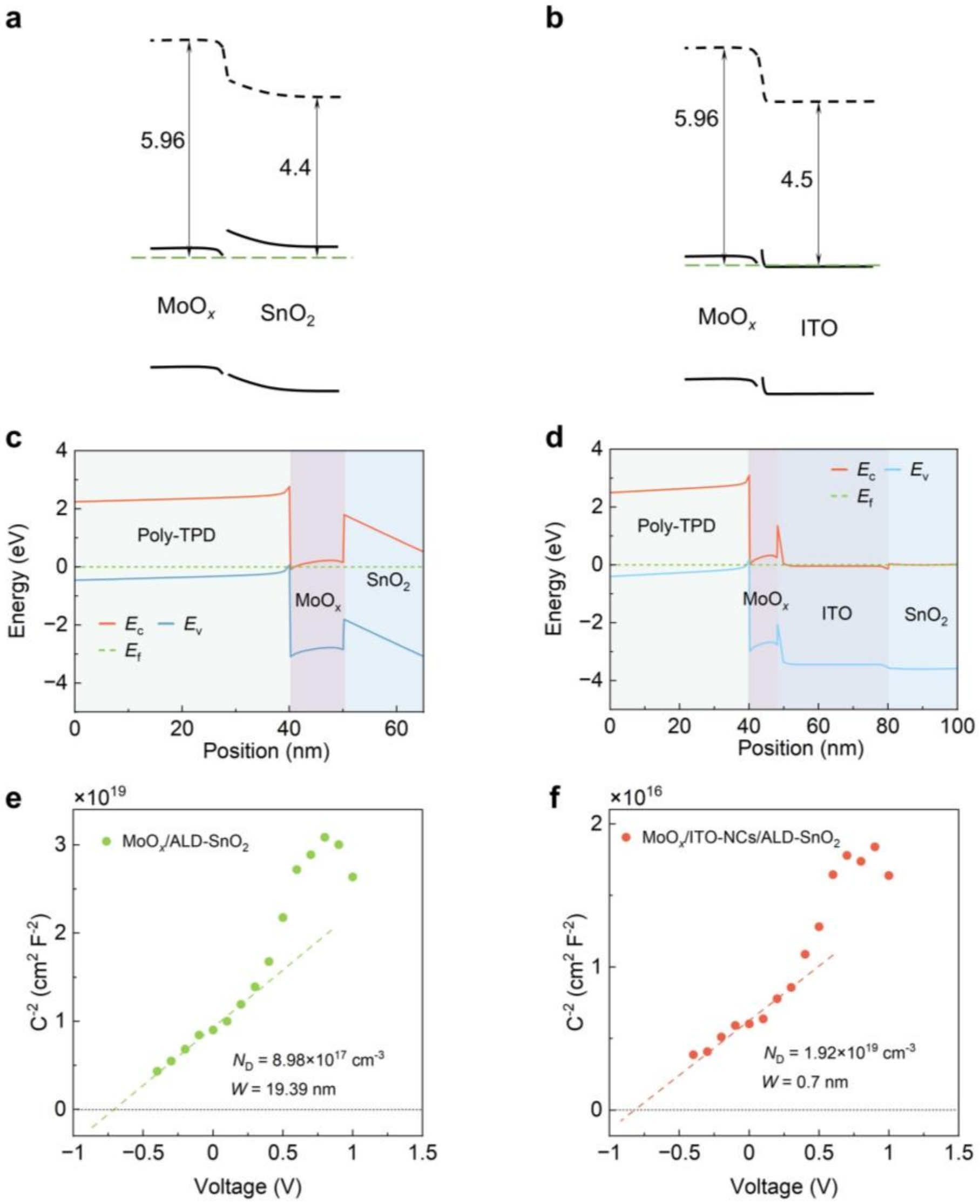

Supplementary Fig. 6 | Simulation and characterization of MoO_x/ALD-SnO_2 and MoO_x/ITO-NCs/ALD-SnO_2 stacks. a,b, Schematic diagrams of band alignment derived from UPS measurements for MoO_x/ALD-SnO_2 (**a**) and MoO_x/ITO-NCs (**b**). **c**,**d**, Simulated energy band alignment of Poly-TPD/MoO_x/ALD-SnO_2 (**c**) and Poly-TPD/MoO_x/ITO-NCs/ALD-SnO_2 (**d**). **e,f,** The C^{-2}-V characteristics of MoO_x/ALD-SnO_2 (**e**) and MoO_x/ITO-NCs/ALD-SnO_2 (**f**). The electron concentration (N_D) and built-in voltage are obtained *via* fitting the slope and intercept of the C^{-2}-V curve according to the Mott-Shockley equation[1,2]. By substituting N_D and built-in voltage into the depletion region width formula[4], the depletion region width (W) of the heterojunctions can be obtained.

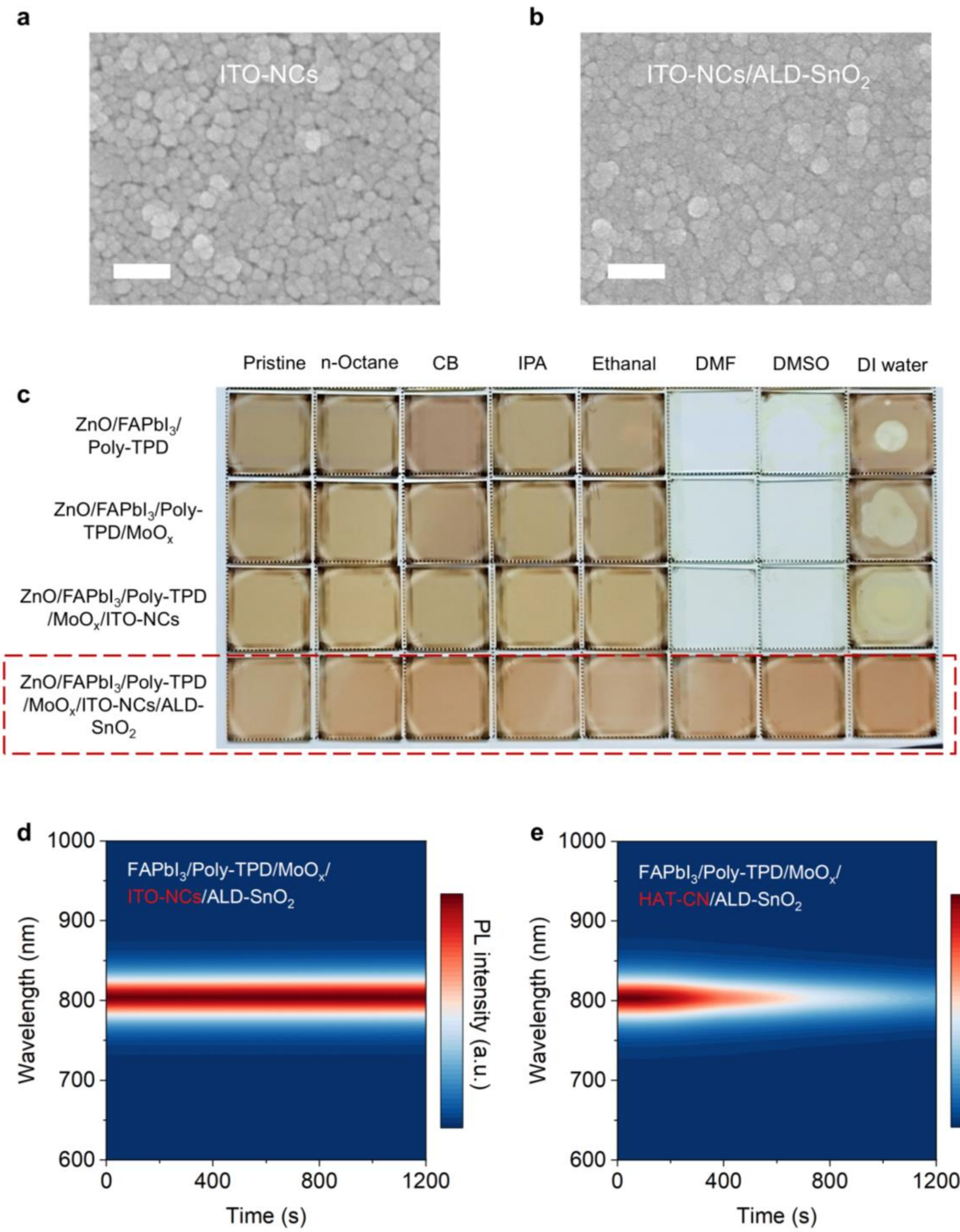

Supplementary Fig. 7 | Film morphology and solvent resistance of ITO-NCs/ALD-SnO_2 composite. a,**b**, Scanning electron microscopy (SEM) images of ITO-NCs before (**a**) and after (**b**) depositing 20 nm ALD-SnO_2. Scale bar, 200 nm. **c,** Solvent resistance of different stacks. For samples with the ALD-SnO_2 coating, the perovskite film remained intact upon spin-coating with strongly polar solvents (water, DMSO, DMF, ethanol) or weakly polar/non-polar solvents (IPA, CB, n-octane). **d,e,** Time-dependent photoluminescence (PL) stability of perovskite films coated with Poly-TPD/MoO_x/ITO-NCs/ALD-SnO_2 (**d**) and Poly-TPD/MoO_x/HAT-CN/ALD-

SnO_2 (**e**) ICLs during the exposure to DMF. After 1200 s of DMF exposure, the perovskite film with the coating of Poly-TPD/MoO_x/ITO-NCs/ALD-SnO_2 ICLs shows negligible variation in the PL intensity. By contrast, the film with the Poly-TPD/MoO_x/HAT-CN/ALD-SnO_2 ICL experiences a notable PL intensity reduction after merely 200 s of DMF exposure. Such distinct differences in solvent resistance provide clear evidence for the superiority of hierarchically structured ITO-NCs/ALD-SnO_2 in preventing the DMF permeation compared to that of HAT-CN/ALD-SnO_2.

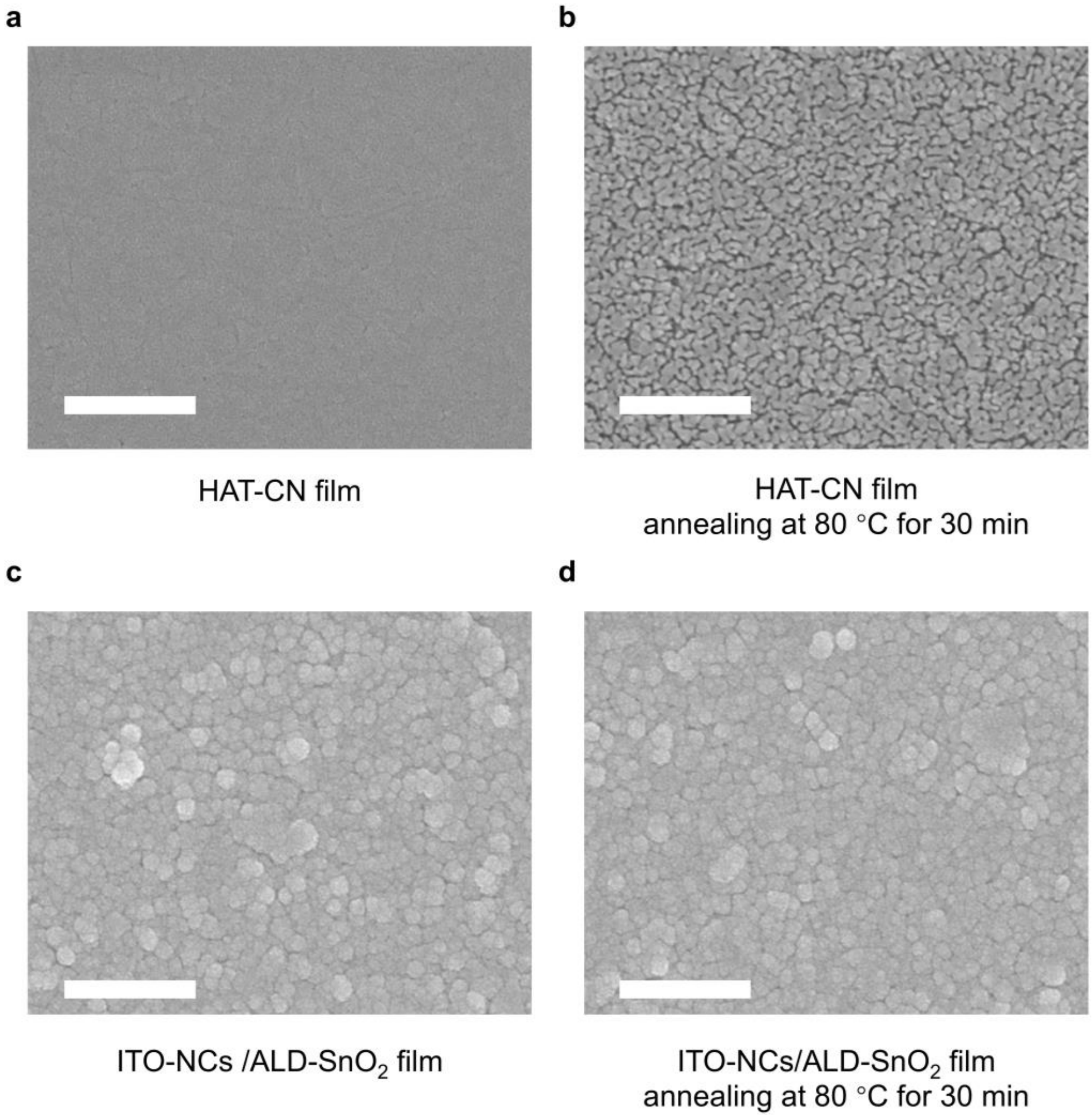

Supplementary Fig. 8 | Thermal-induced morphology change of HAT-CN and ITO-NCs/ALD-SnO_2. a,**b**, Scanning electron microscopy images of HAT-CN film before (**a**) and after (**b**) annealing at 80 °C for 30 min. Scale bar, 500 nm. **c**,**d**, Scanning electron microscopy images of ITO-NCs/ALD-SnO_2 film before (**c**) and after (**d**) annealing at 80 °C for 30 min. Scale bar, 300 nm.

Dewetting of an organic film is a spontaneous process where a thin organic film on a substrate tends to rupture or break up into isolated, disjointed droplets or patterns, which is widely observed in most OLED and OPV devices[4–6]. Owing to the relatively low glass transition temperature of many organics, even mild thermal treatments (*e.g.*, 80-120 °C, typical annealing temperature for perovskite films) can induce or accelerate dewetting during the processing of tandem PeLEDs. The dewetting would easily compromise the structural integrity of as-formed functional layers (*e.g.*, an ICL comprising small organic molecules), which inevitably deteriorate the electrical properties and solvent resistance of the integral ICL. Compared with

HAT-CN that undergoes distinct structural deterioration under thermal stimulation, ITO-NCs/ALD-SnO_2 materials exhibit excellent thermal morphological stability. This stable structural feature further verifies the great superiority of ITO-NCs as high-performance bridging layers for multijunction PeLEDs.

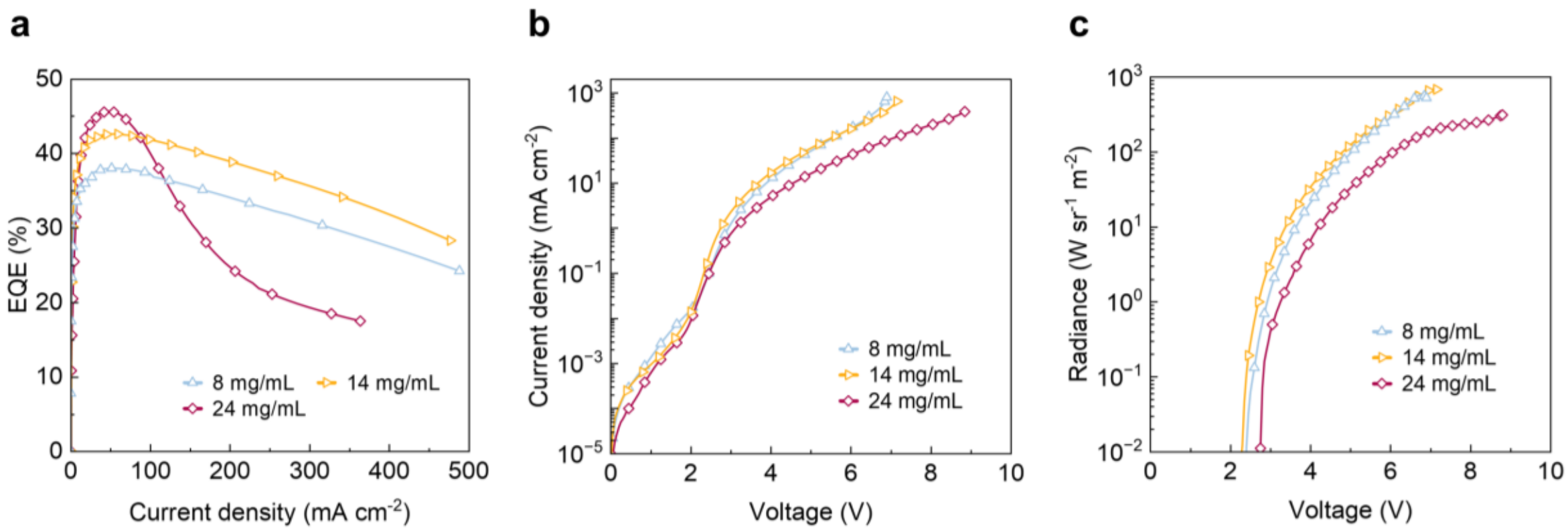

Supplementary Fig. 9 | $FAPbI_3$/$FAPbI_3$ PeLEDs with varied thicknesses of Poly-TPD. a-c, EQE-*J* (**a**), *R*-*V* (**b**), and *J*-*V* (**c**) curves. The thickness of Poly-TPD was simply controlled by tuning the solution concentration.

The improved peak EQE can be attributed to the enhanced photon-recycling effect induced by thicker hole-transport layers[7]. However, these thick films unavoidably exacerbate Joule heating at high current densities, which accelerates efficiency roll-off. Concurrently, excessive heat buildup further suppresses the maximum achievable radiance.

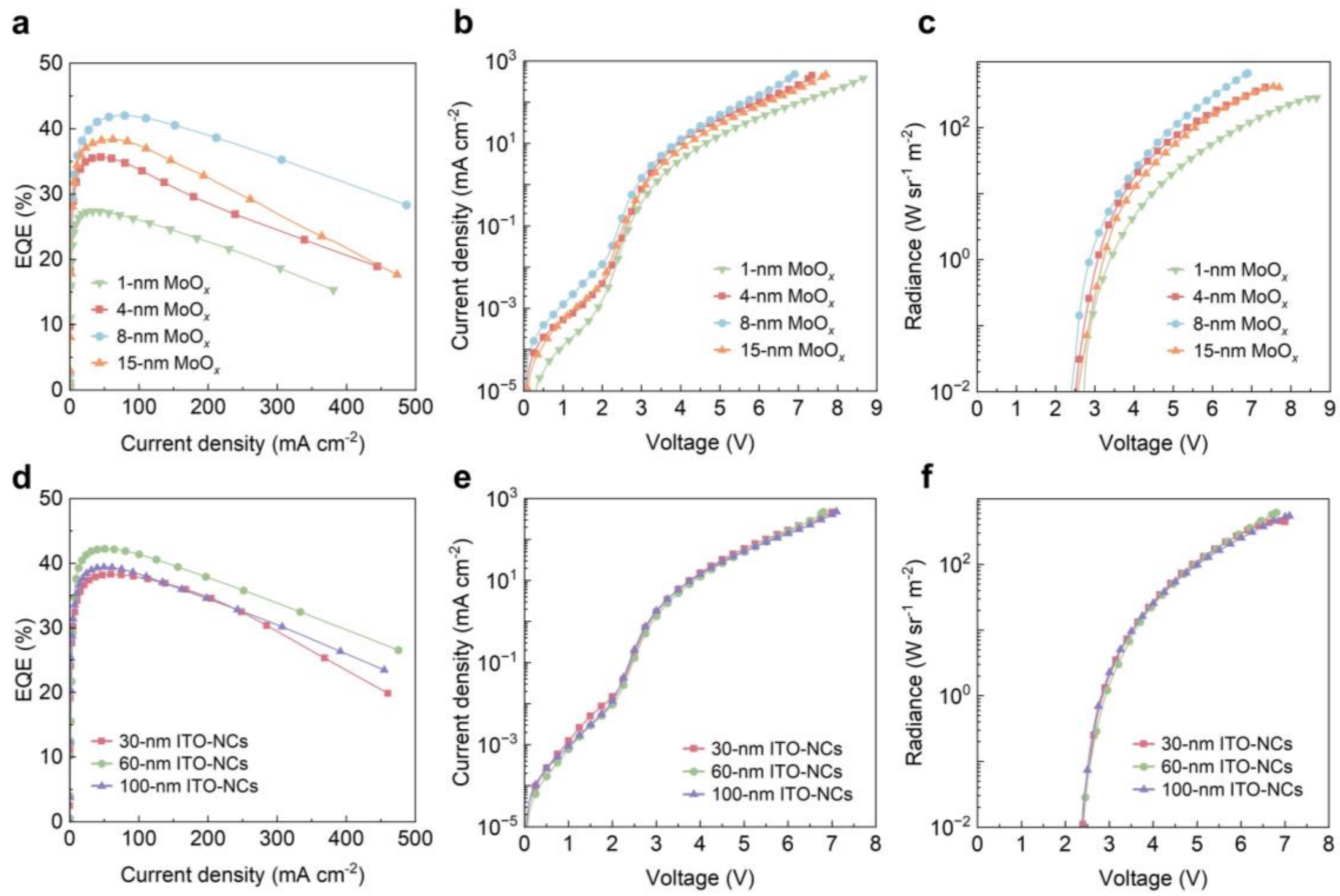

Supplementary Fig. 10 | Performance of double-junction $FAPbI_3$/$FAPbI_3$ LEDs with varied ICLs. a-c, The influence of MoO_x thickness on the device performance. EQE-*J* (**a**), *J*-*V* (**b**), and *R*-*V* (**c**) characteristics. **d**-**f**, The influence of ITO-NCs thickness on the device performance. EQE-*J* (**d**), *J*-*V* (**e**), and *R*-*V* (**f**) characteristics. The thickness of ITO-NC layers is tuned by spin-coating ITO-NC dispersions with different concentrations. Specifically, 30-nm-, 60-nm-, and 100-nm-thick ITO-NC films were fabricated by spin-coating 3 wt%, 5 wt%, and 8 wt% precursor solutions at 5000 rpm for 30 s, respectively.

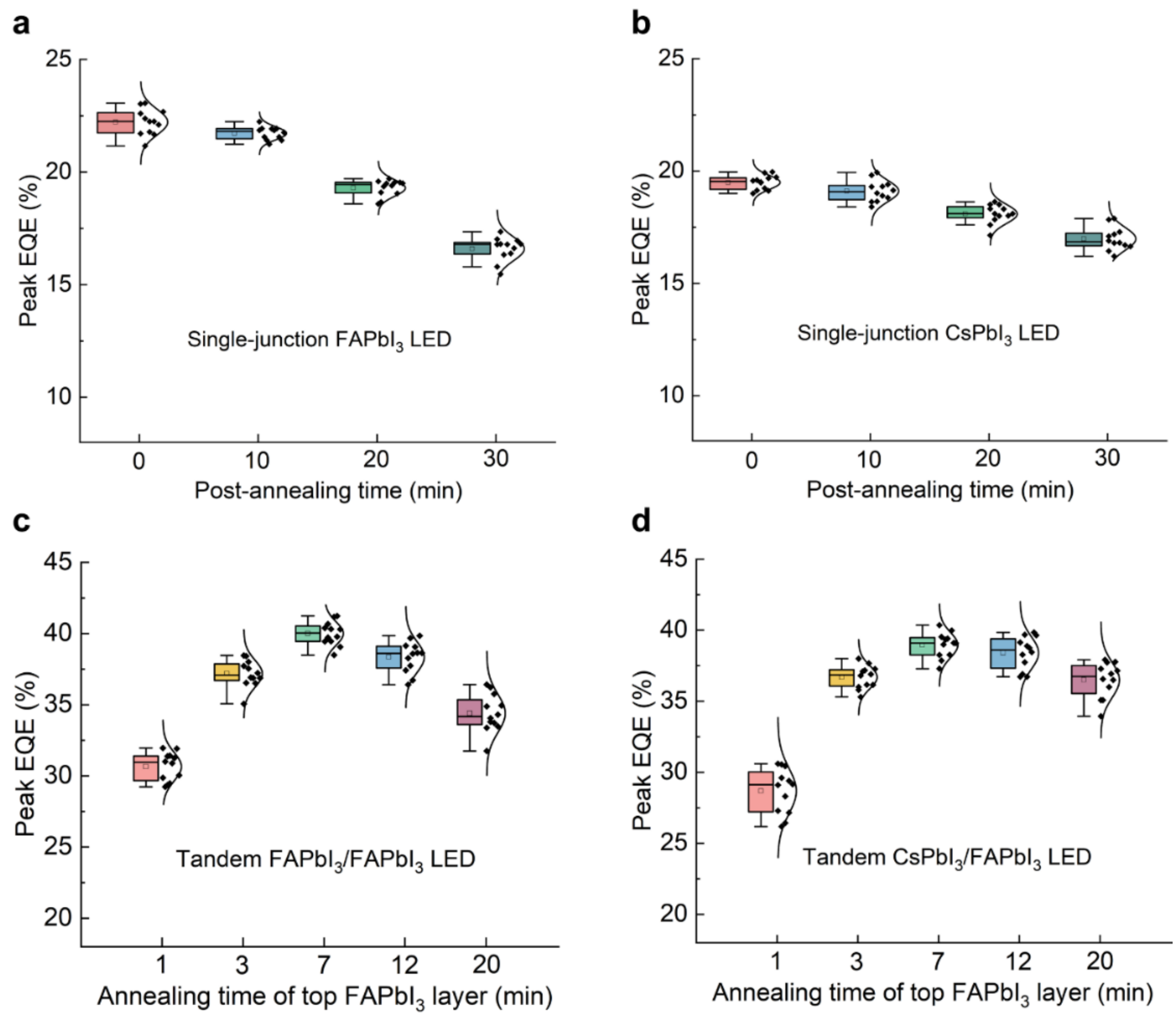

Supplementary Fig. 11 | Thermal effects of single-junction and tandem perovskite LEDs. **a**, **b**, Peak EQEs of single-junction $FAPbI_3$ (**a**) and $CsPbI_3$ LEDs (**b**) at different post-annealing time. All annealing processes were conducted at 100 °C after depositing Poly-TPD. **c**, **d**, Peak EQEs of tandem $FAPbI_3/FAPbI_3$ (**c**) and $CsPbI_3/FAPbI_3$ (**d**) LEDs with different annealing time for top $FAPbI_3$ emissive layer.

While the single-junction $CsPbI_3$ PeLED degrades after thermal treatment at 100 ℃ for more than 20 minutes and continues to deteriorate after 30 minutes, the $FAPbI_3$ PeLED exhibits a much more severe performance drop under the same conditions, indicating that $CsPbI_3$ possesses better thermal stability. Specifically, the $CsPbI_3$ single-junction device retains approximately 86% of its initial EQE after 30 minutes of thermal annealing at 100 ℃, whereas the EQE retention of the $FAPbI_3$ device over the same duration is significantly lower. This result is consistent with our results that single-junction $CsPbI_3$ PeLEDs have longer operational stability (**Supplementary Figure 15**). Since both tandem devices share the same top $FAPbI_3$ subunit, the performance evolution of the $FAPbI_3/FAPbI_3$ and $CsPbI_3/FAPbI_3$ devices during

thermal annealing of the $FAPbI_3$ layer is essentially identical. However, thanks to the superior thermal stability of the bottom $CsPbI_3$ subunit, the $CsPbI_3/FAPbI_3$ tandem device achieves an operational stability of 534 hours, considerably longer than the 160 hours of the $FAPbI_3/FAPbI_3$ device (**Supplementary Figure 15**). This result supports the necessity of improving the thermal stability of each subunit to achieve ideal EQE scaling in higher-junction tandems ($N \geq 3$).

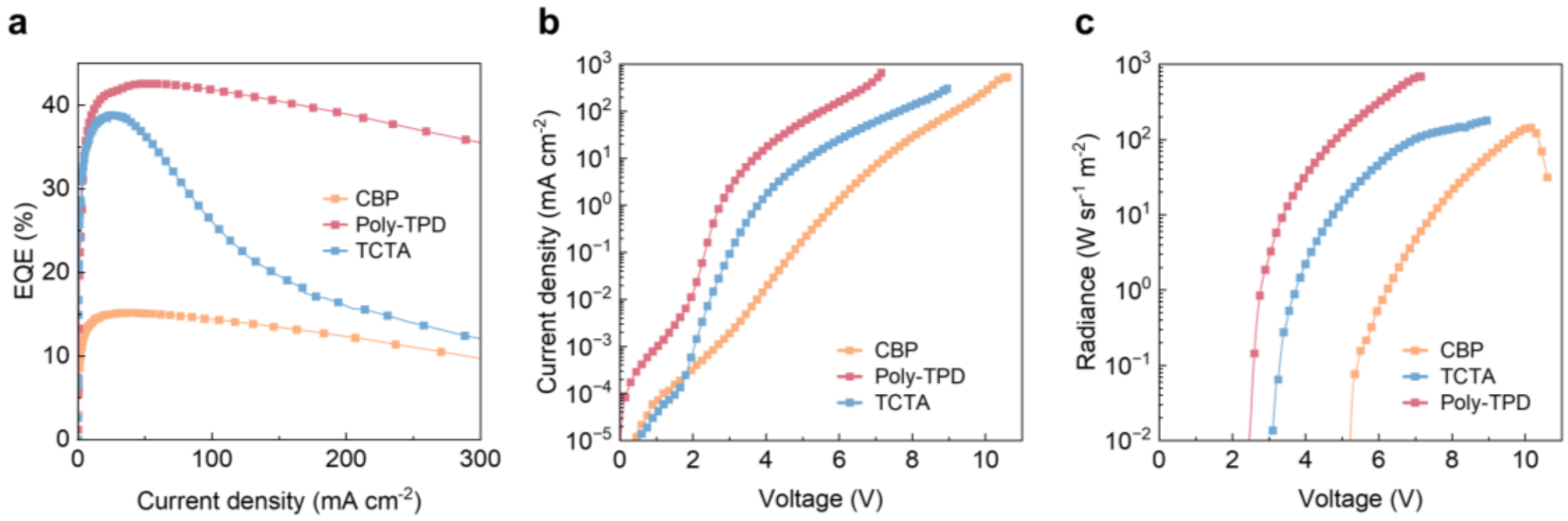

Supplementary Fig. 12 | $FAPbI_3$/$FAPbI_3$ PeLEDs utilizing ICLs with different HTL. a, EQE-*J* curves. **b**, *J*-*V* curves. **c**, *R*-*V* curves.

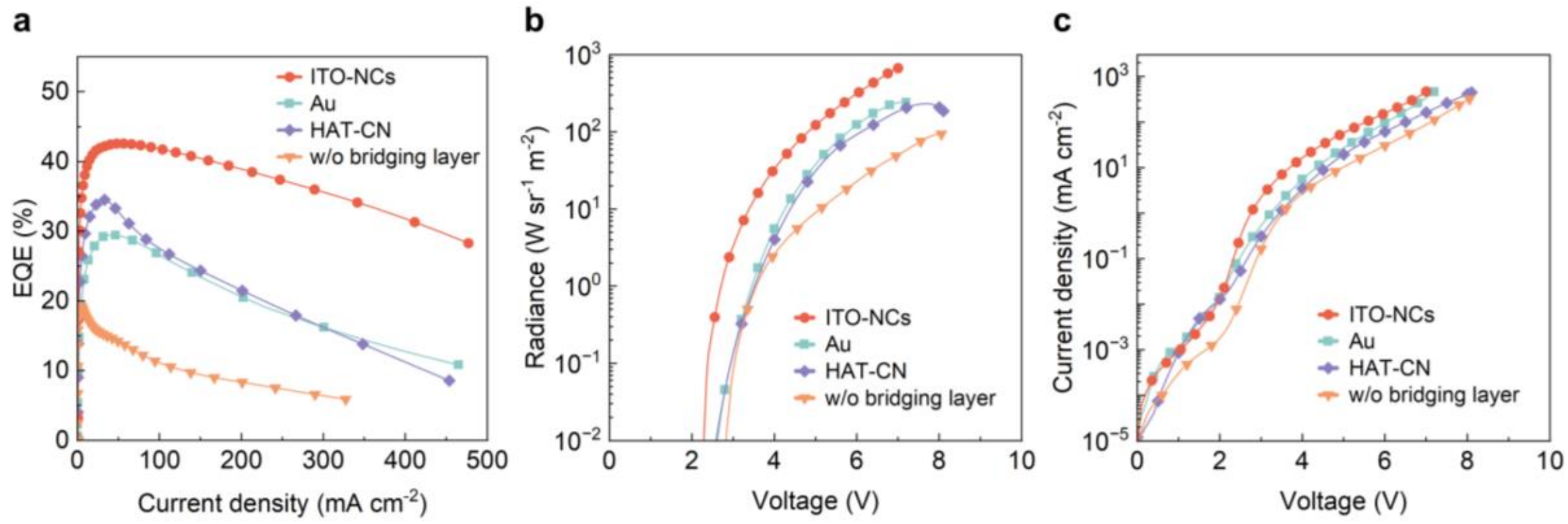

Supplementary Fig. 13 | $FAPbI_3$/$FAPbI_3$ PeLEDs utilizing ICLs with different bridging layers. a, EQE-*J* curves. **b**, *R*-*V* curves. **c**, *J*-*V* curves.

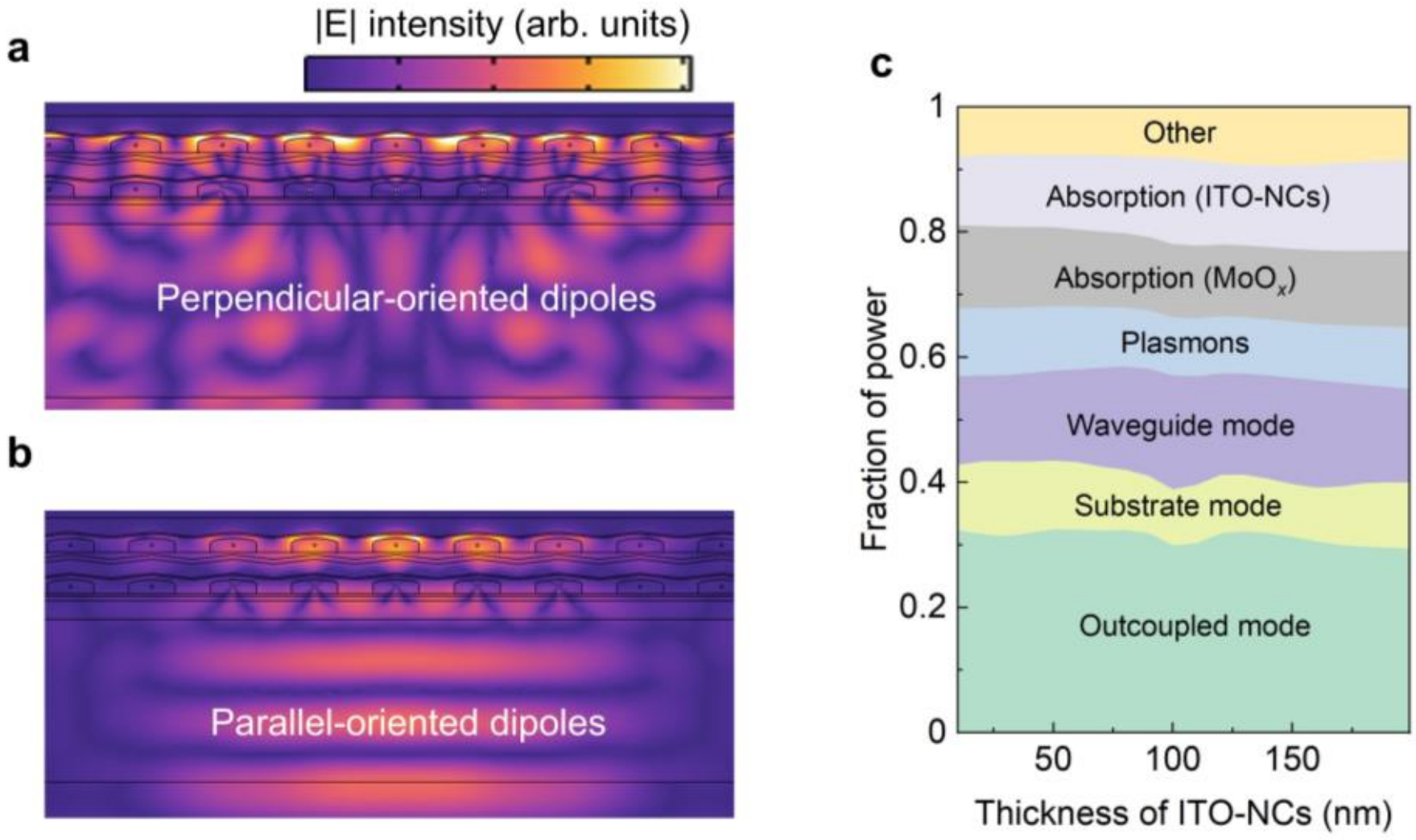

Supplementary Fig. 14 | Optical simulations of tandem PeLEDs. a,b, Normalized electric field profiles of $FAPbI_3/FAPbI_3$ tandem structures. Parallel- (**a**) and perpendicular-oriented (**b**) dipoles, monitored at 800 nm as illustrated. **c**, Simulated optical power distribution in tandem structures as a function of ITO-NCs layer thickness.

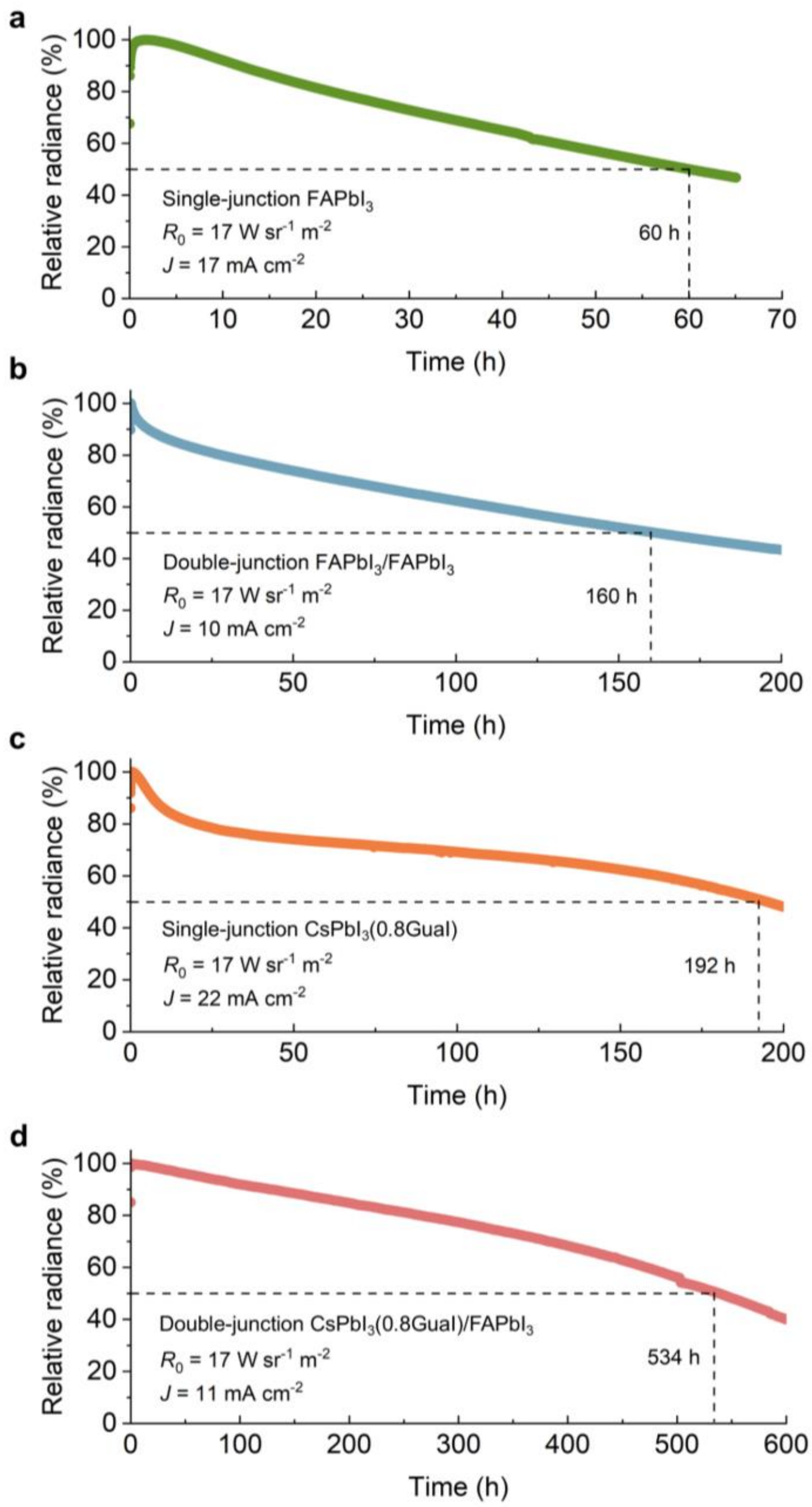

Supplementary Fig. 15 | Operational stability of single-junction and double-junction PeLEDs. a, Single-junction $FAPbI_3$ PeLED. **b**, Double-junction $FAPbI_3$/$FAPbI_3$ PeLED. **c**, Single-junction $CsPbI_3$(0.8GuaI) PeLED **d**, Double-junction $CsPbI_3$(0.8GuaI)/$FAPbI_3$ PeLED. The operational stability was evaluated at an initial radiance (R_0) of approximately 17 W sr^{-1} m^{-2} for the tandem and single-junction devices.

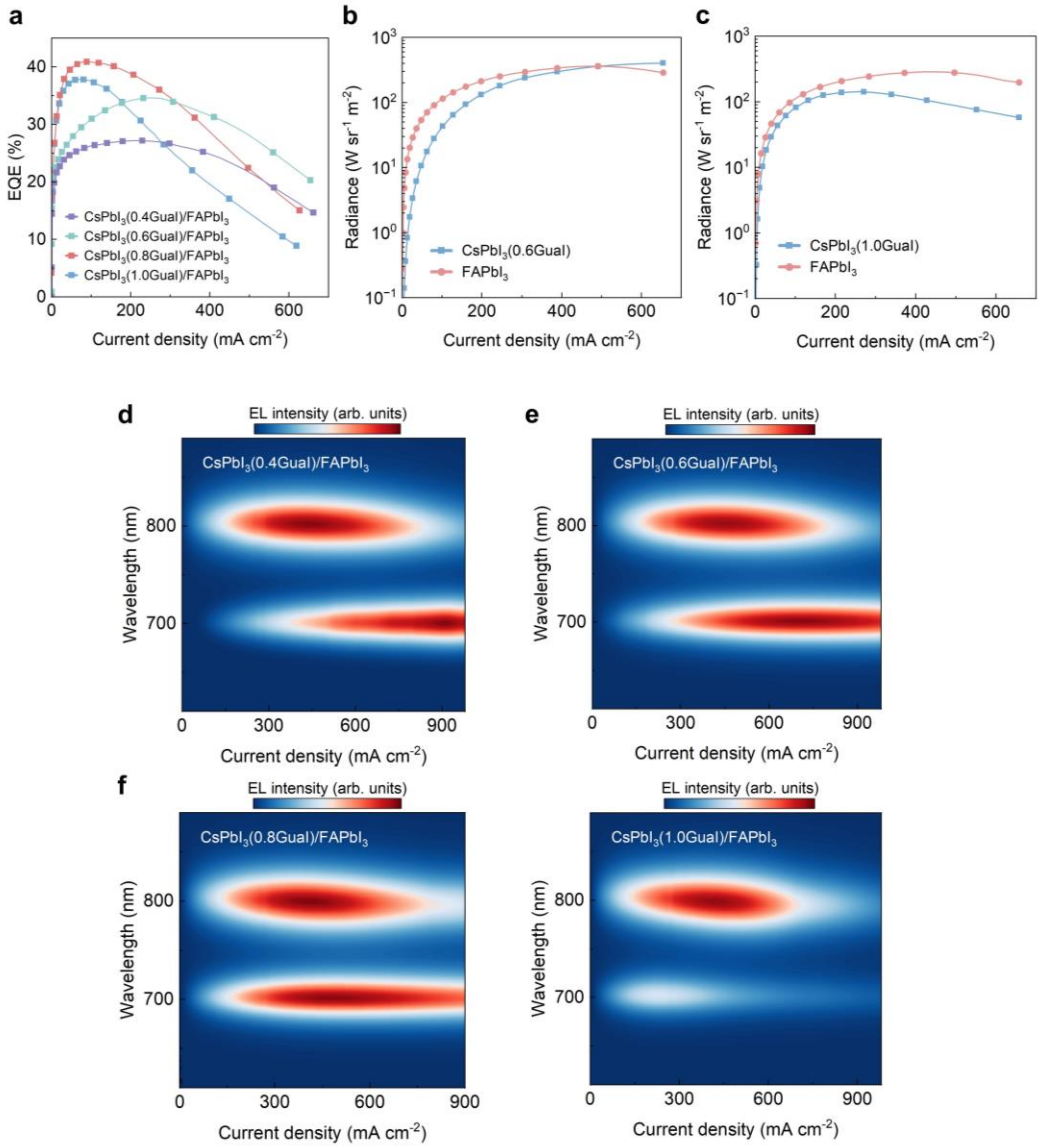

Supplementary Fig. 16 | Dual-emission $CsPbI_3$(*y*GuaI)/$FAPbI_3$ tandem PeLEDs. a, EQE-*J* curves of the dual-emission $CsPbI_3$(*y*GuaI)/$FAPbI_3$ PeLEDs with varied GuaI additive in the $CsPbI_3$ precursor. **b,c,** *R*-*J* curves of the subunits in the $CsPbI_3$(0.6GuaI)/$FAPbI_3$ (**b**) and $CsPbI_3$(1.0GuaI)/$FAPbI_3$ (**c**) tandem PeLEDs. *R*-*J* curves of subunits were extracted from the EL characteristics of corresponding tandem PeLEDs. **d-g**, Current density-dependent EL spectra of the tandem PeLEDs comprising $CsPbI_3$(0.4GuaI)/$FAPbI_3$ (**d**), $CsPbI_3$(0.6GuaI)/$FAPbI_3$ (**e**), $CsPbI_3$(0.8GuaI)/$FAPbI_3$ (**f**), and $CsPbI_3$(1.0GuaI)/$FAPbI_3$ (**g**) units.

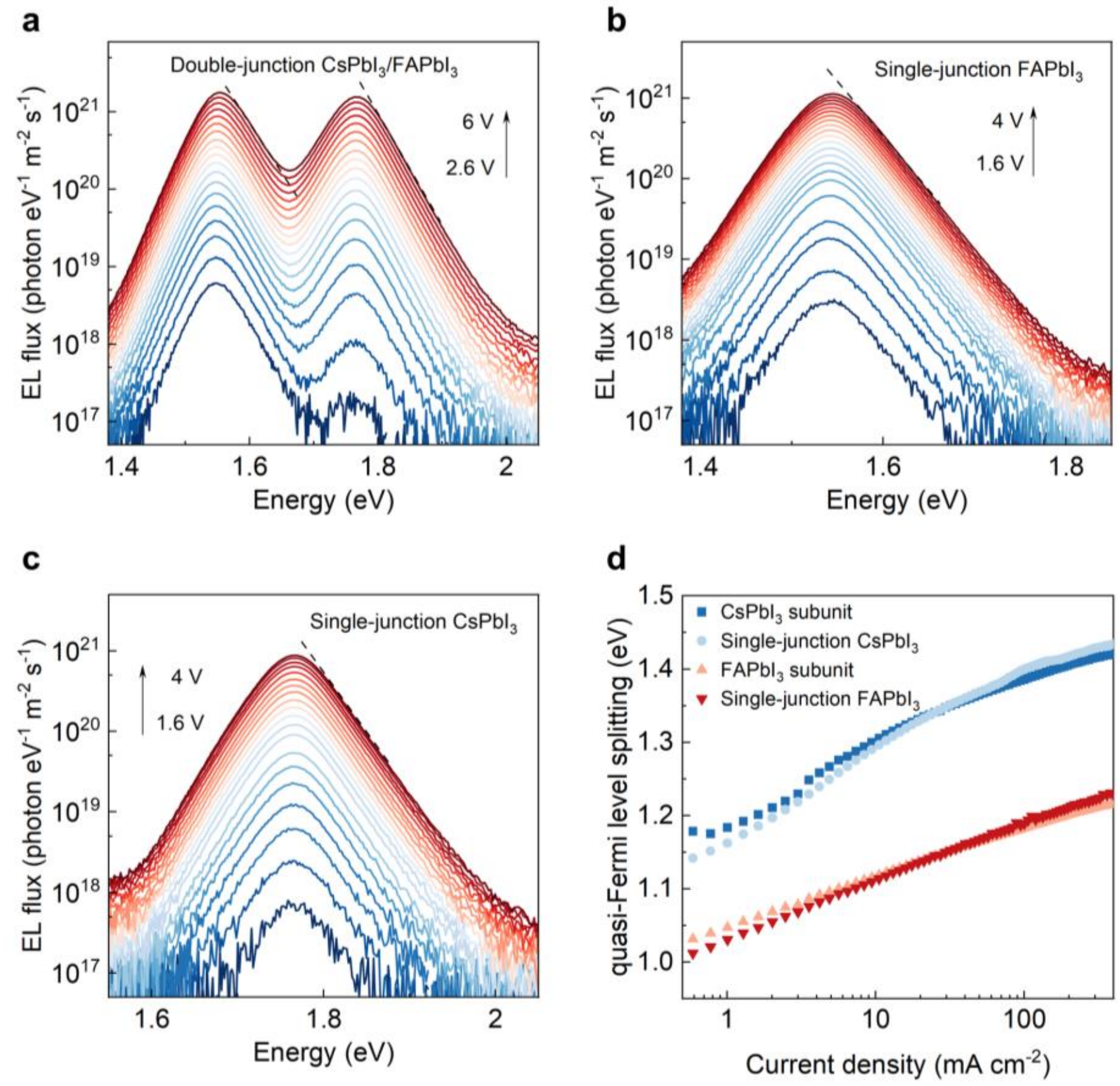

Supplementary Fig. 17 | Optimized dual-emission tandem PeLEDs. a-c, EL spectra of the two-junction $CsPbI_3$(0.8GuaI)/$FAPbI_3$ (**a**), single-junction $FAPbI_3$ (**b**) and $CsPbI_3$(0.8GuaI) (**c**) devices under varied biases. Dashed lines indicate spectral fitting regions used to extract the quasi-Fermi level splitting (QFLS) of each subunit. **d**, Comparison of the extracted QFLS values between the subunits within tandem devices and the corresponding single-junction devices. The value of QFLS divided by the elementary charge (QFLS/q) can be used to reflect the voltage drop of the emissive layer associated with radiative recombination.

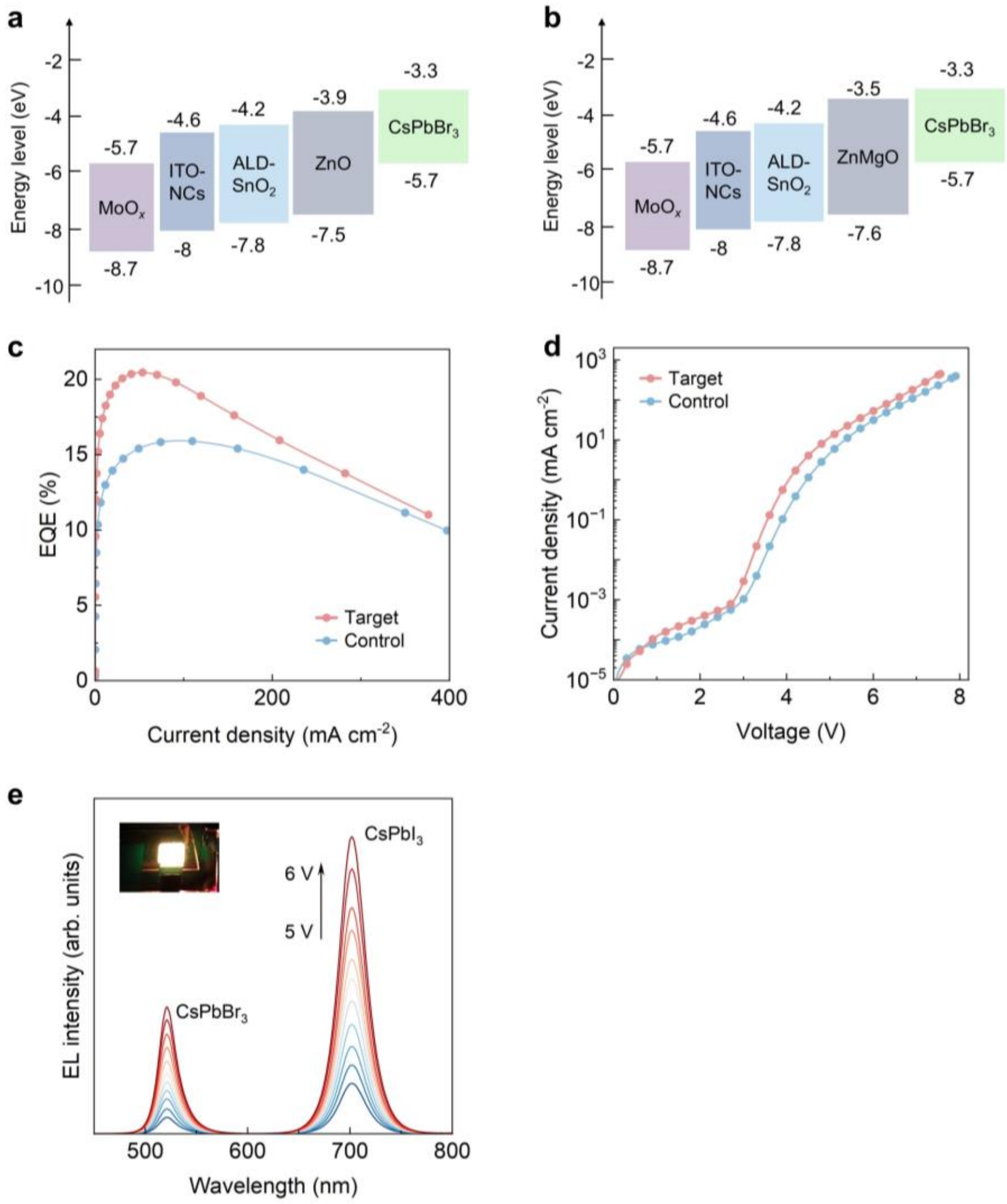

Supplementary Fig. 18 | Dual-emission $CsPbI_3$/$CsPbBr_3$ tandem LEDs. a,b, Energy levels of partial functional layers in control (**a**) and target (**b**) tandem devices. **c,** EQE-*J* curves. **d,** *J*-*V* curves. **e,** EL spectrum of $CsPbI_3$/$CsPbBr_3$ tandem device under varying applied biases. The inset shows device photographs under operation.

To promote a favorable energy-level alignment, magnesium-doped ZnO (ZnMgO) was introduced to modify the electron-injection contact for the bromide-based wide-bandgap subunit. With this contact modification, the device shows reduced series resistance and improved EQE. However, the performance of the resulting $CsPbI_3$/$CsPbBr_3$ (red/green) tandem device still lags behind that of their red/near-infrared and near-infrared/near-infrared counterparts, due to the challenges in fabricating high-performance $CsPbBr_3$ units on metal-oxide-based electron-transporting layers[8].

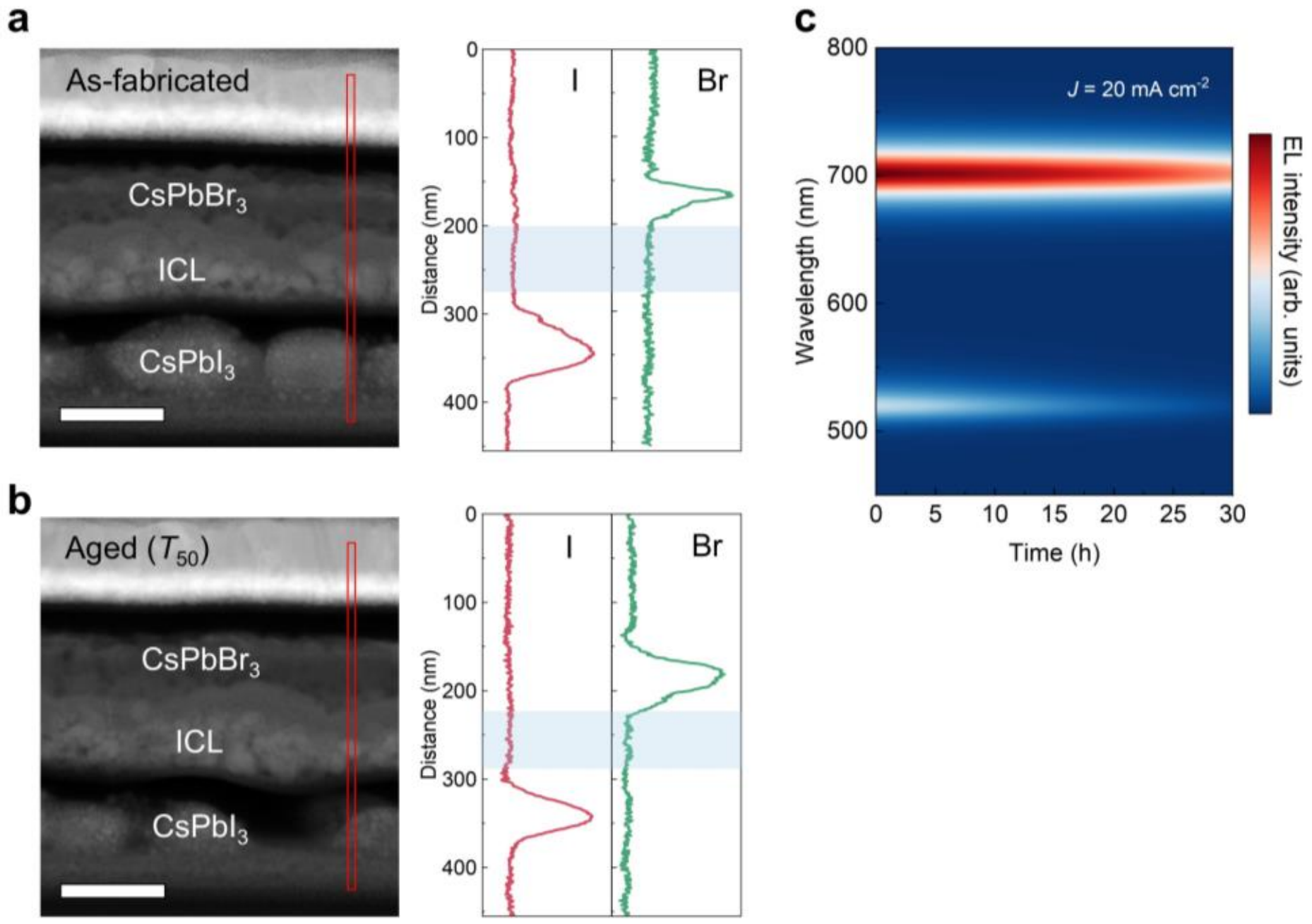

Supplementary Fig. 19 | Characterization of halide interdiffusion in $CsPbI_3/CsPbBr_3$ tandem LEDs. a,b, The cross-sectional STEM and EDS measurements on as-fabricated (**a**) and aged (T_{50}) (**b**) $CsPbI_3/CsPbBr_3$ tandem devices. Scale bar, 100 nm. **c**, Time-dependent EL spectrum of tandem $CsPbI_3/CsPbBr_3$ device driven under 20 mA cm^{-2}.

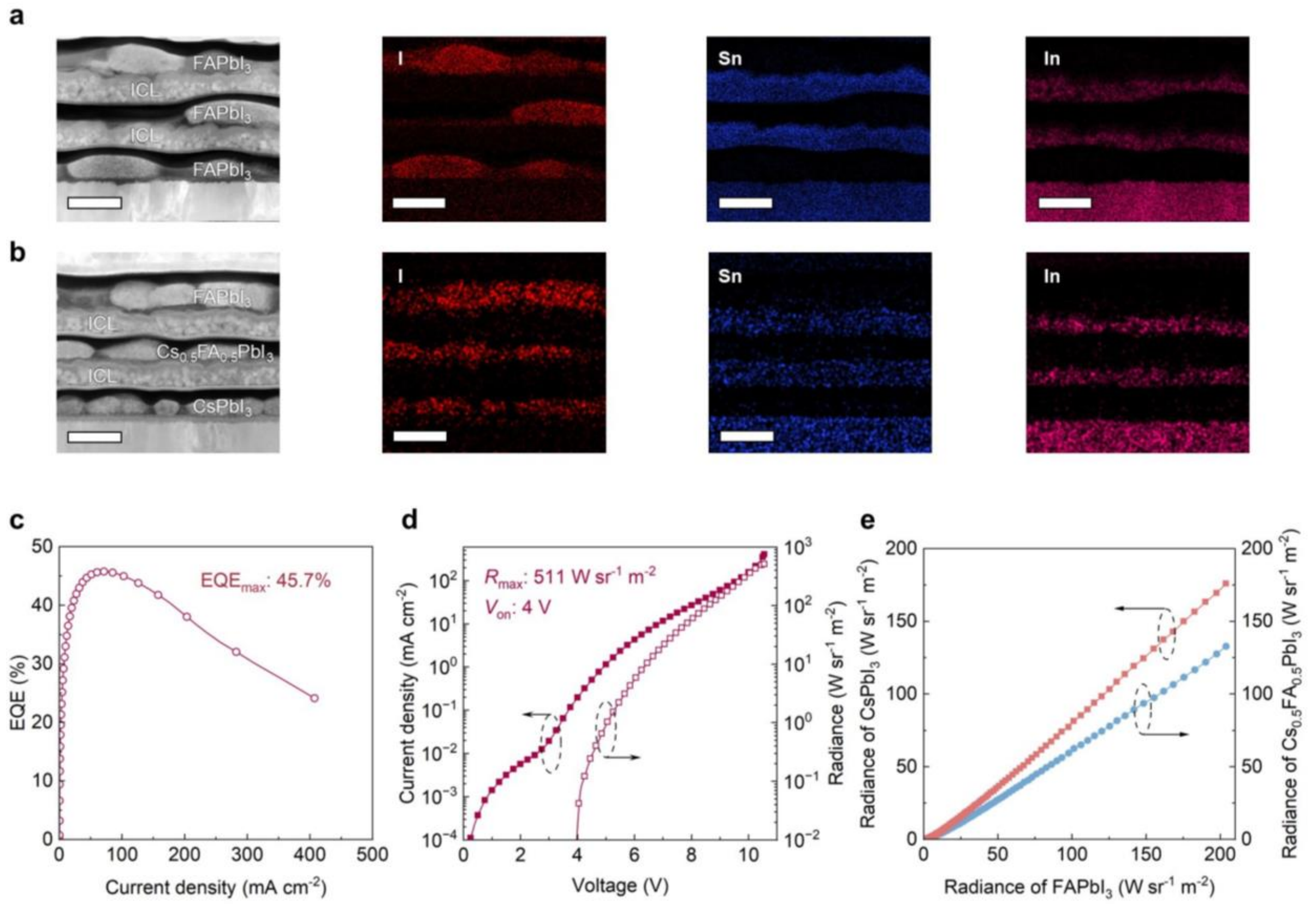

Supplementary Fig. 20 | Triple-junction PeLEDs. a, b, Cross-sectional HAADF-STEM and EDS composite mapping images of triple-junction PeLEDs comprising three $FAPbI_3$ units (**a**) and $CsPbI_3/FA_{0.5}Cs_{0.5}PbI_3/FAPbI_3$ subunit stacks (**b**). Scale bar, 200 nm. **c-e,** EQE-*J* (**c**), *J-V-R* (**d**), and *R-R* curves (**e**) of the triple-junction $CsPbI_3/FA_{0.5}Cs_{0.5}PbI_3/FAPbI_3$ device.

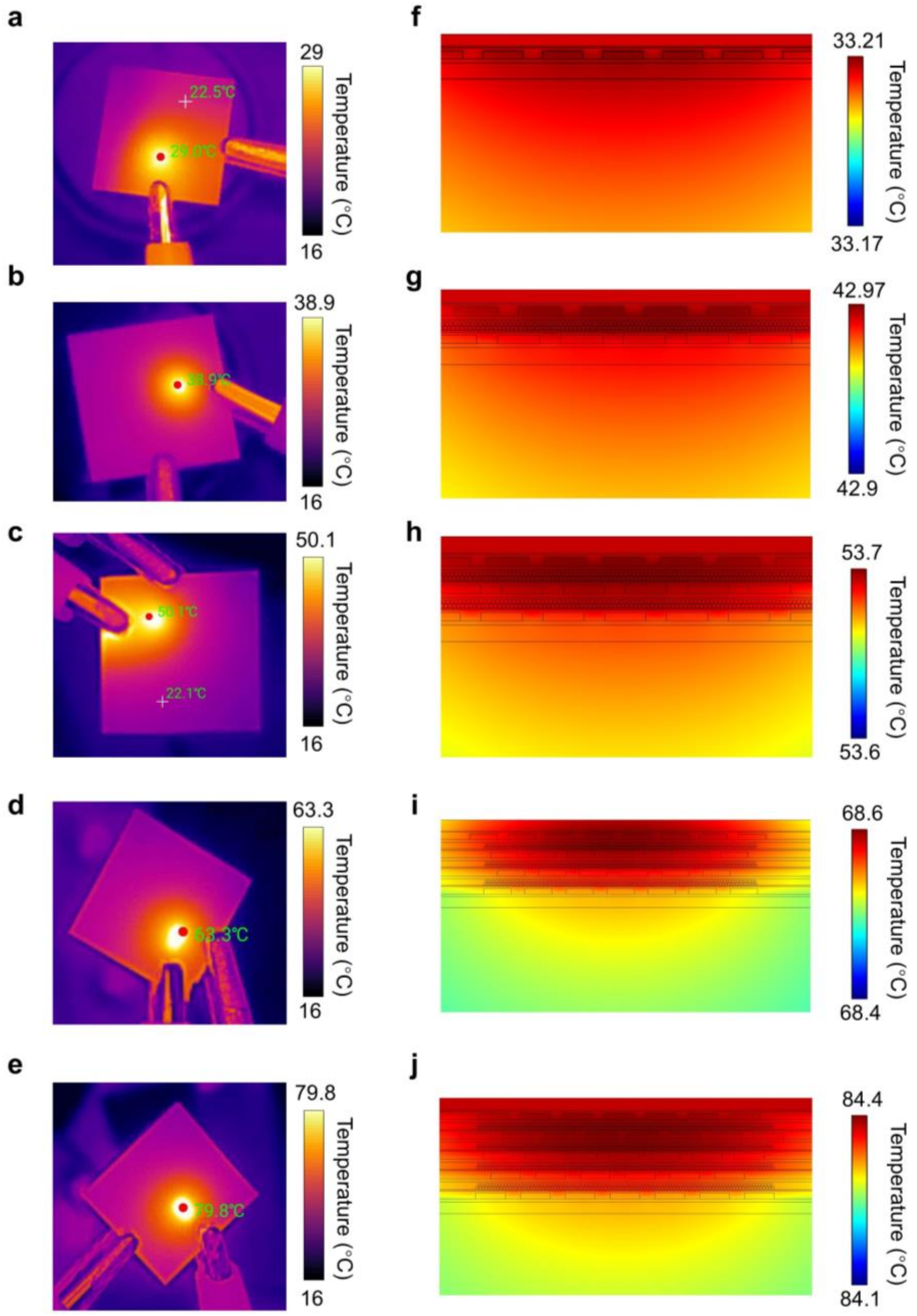

Supplementary Fig. 21 | The temperature characteristics of single- and multi-junction tandem PeLEDs. a-e, Infrared-thermal-imaging pictures of the single- (**a**), double- (**b**), triple-junction (**c**), four- (**d**), five-junction (**e**) PeLEDs after operating at a current density of 400 mA cm^{-2} for 10 s. **f-j**, Simulated cross-sectional temperature profiles of the single- (**f**), double- (**g**), triple-junction (**h**), four- (**i**), and five-junction (**j**) PeLEDs.

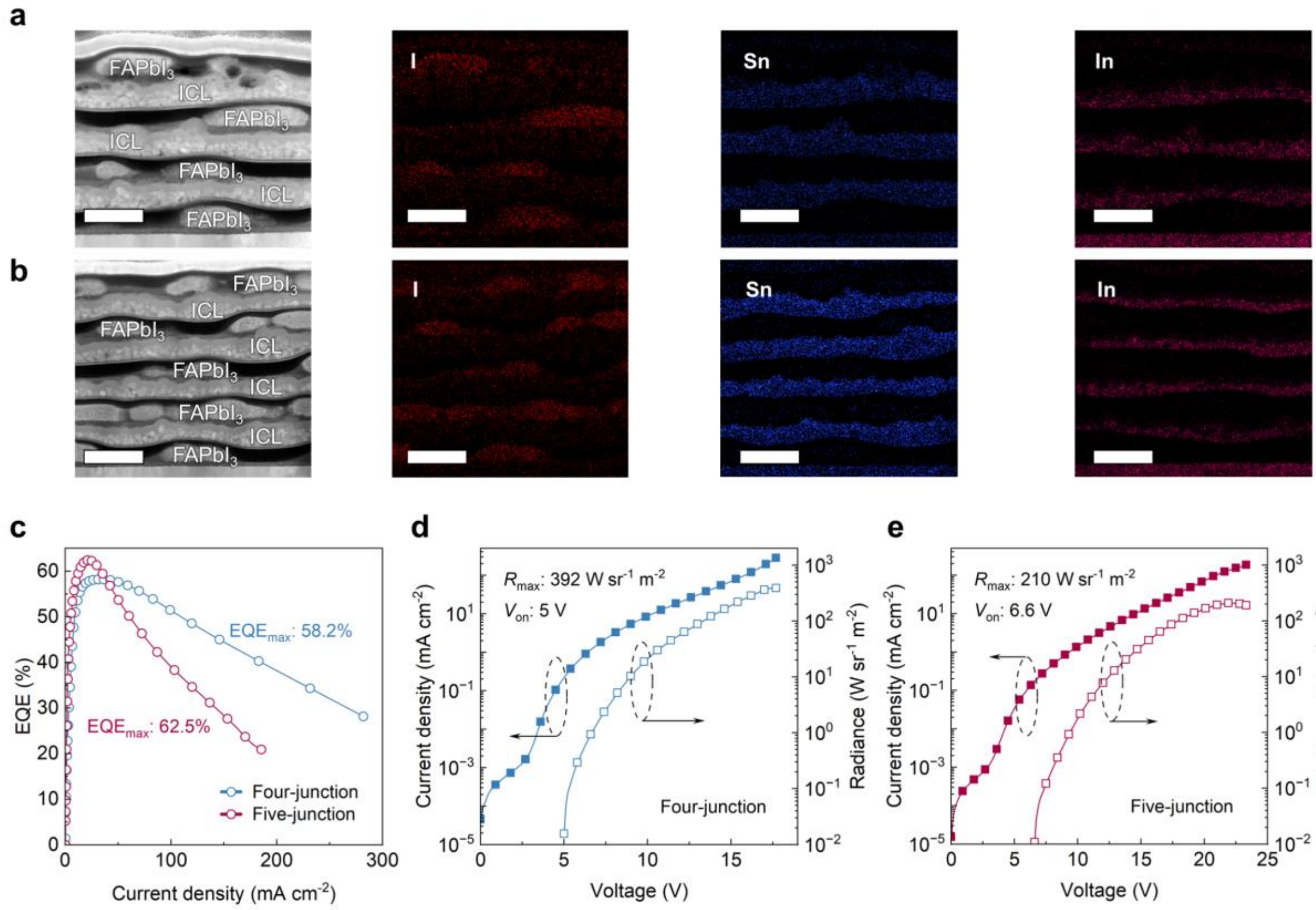

Supplementary Fig. 22 | Four- and five-junction PeLEDs. a,b, Cross-sectional HAADF-STEM and EDS composite mapping images of multijunction PeLEDs comprising four (**a**) and five $FAPbI_3$ units (**b**). Scale bar, 200 nm. **c,** EQE-*J* curves of the multijunction $FAPbI_3$ PeLEDs. **d,e,** *J*-*V*-*R* curves of four- (**d**) and five-junction (**e**) $FAPbI_3$ PeLEDs.

The peak EQE of an *N*-junction tandem PeLED is theoretically expected to be *N* times that of a single-junction device. Unfortunately, the solution-based fabrication of perovskite subunits necessitates repeated thermal annealing, which inevitably exposes the bottom perovskite layers to prolong thermal stress. Specifically, as shown in Supplementary Fig. 11, the performance of a single-junction $FAPbI_3$ PeLED degrades after thermal treatment at 100 °C for over 20 minutes, confirming the moderate thermal stability of the perovskite films used herein. It should be noted that this perovskite film adopts a standard early-stage formulation[9], without any targeted modifications to enhance its thermal stability. Thus, to achieve the ideal EQE scaling behavior for tandem devices with $N \geq 3$, advanced strategies, such as interface engineering or thermal management optimization, are indispensable.

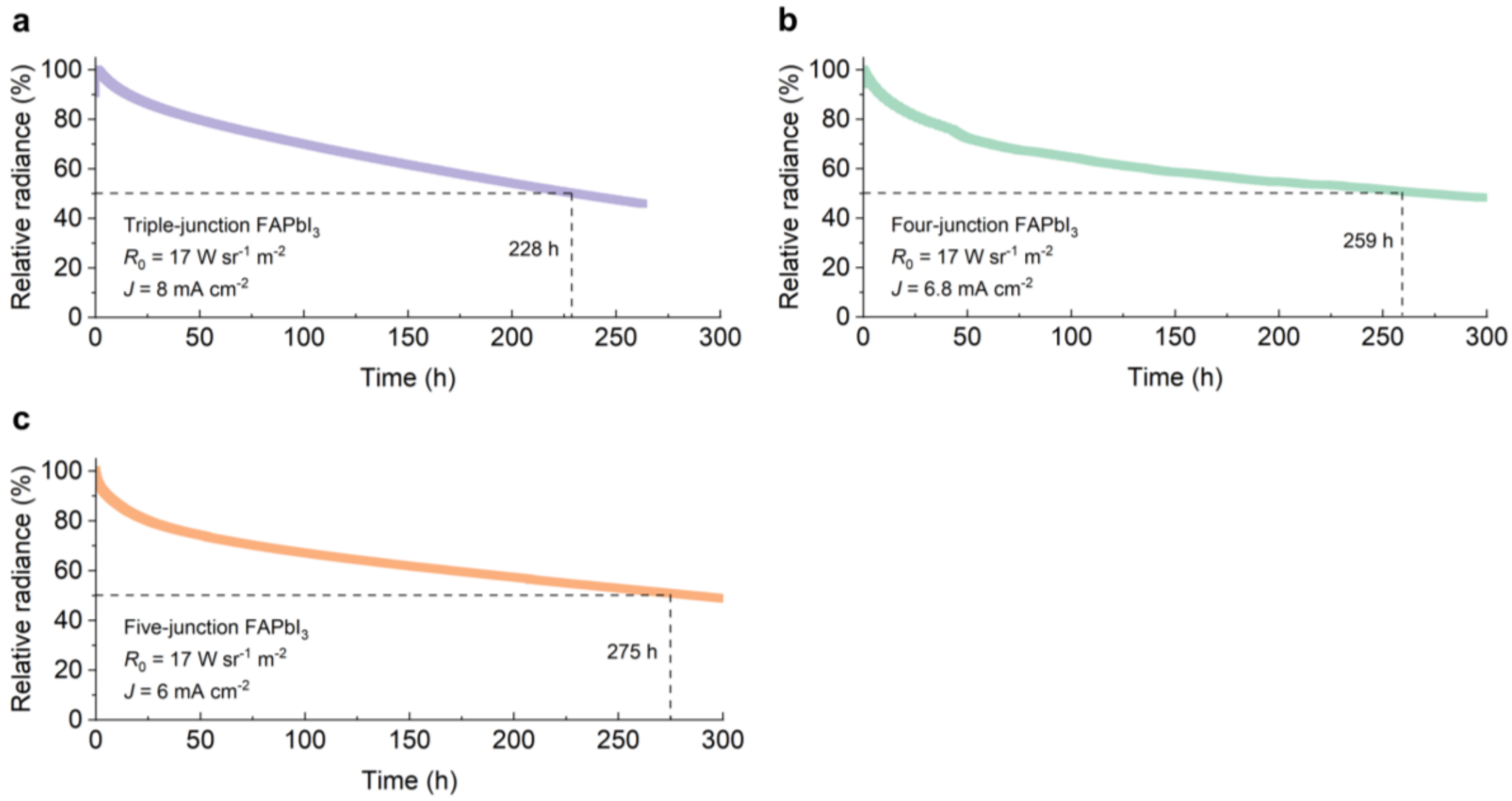

Supplementary Fig. 23 | Operational stability of multijunction-junction PeLEDs. a-c, Triple- (**a**), four- (**b**), and five-junction (**c**) $FAPbI_3$ PeLEDs. The operational stability was evaluated at an initial radiance (R_0) of approximately 17 W sr^{-1} m^{-2}.

The lifetime increases with the number of junctions because the current density required for a given radiance decreases proportionally. We also note that the improvement becomes less pronounced as the junction number increases, stemming from sustained thermal stress on underlying perovskite layers (Supplementary Fig. 15). To further improve the operational lifetime of higher junction PeLEDs, future work will focus on thermally stable perovskite protocol.

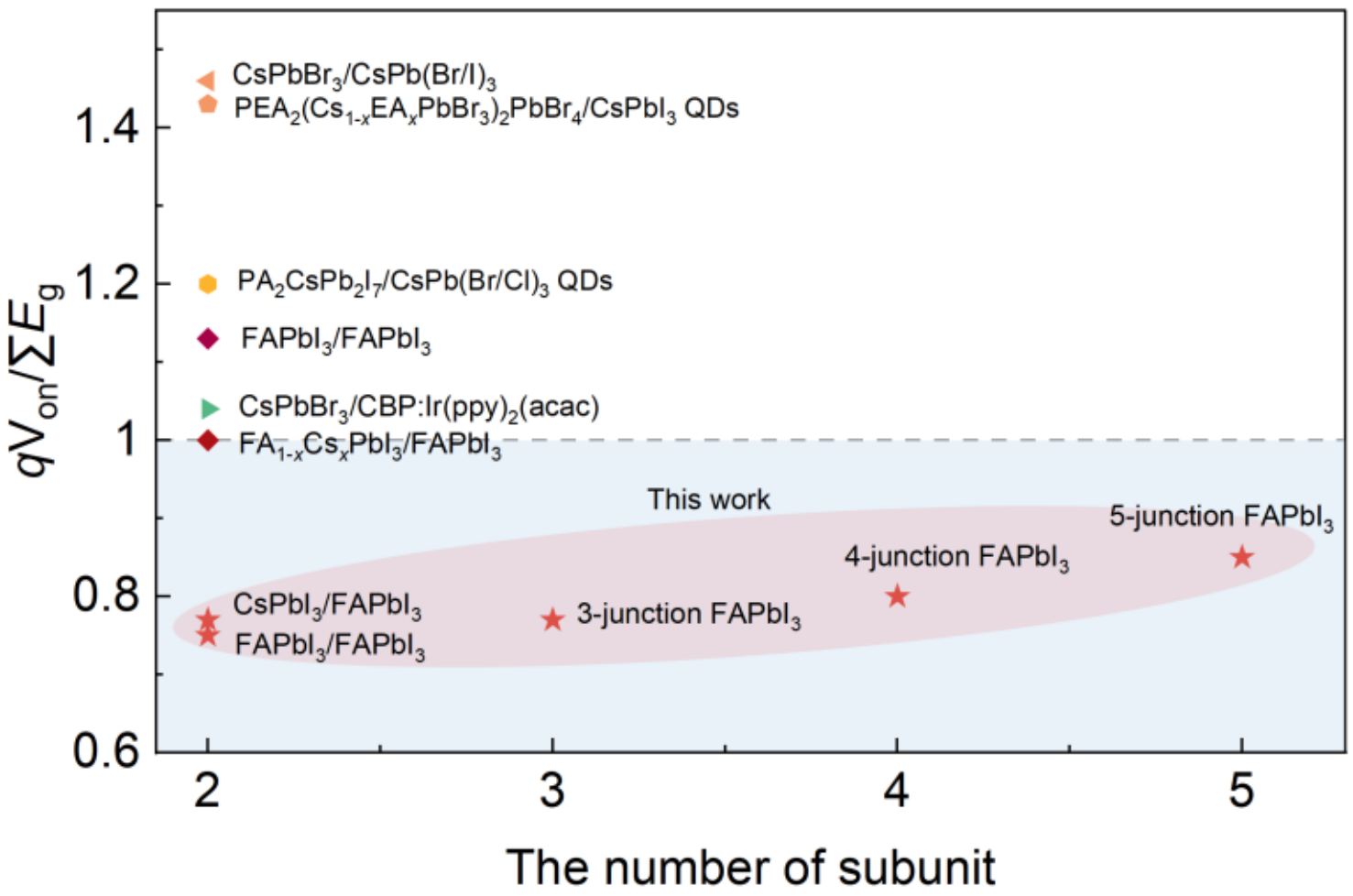

Supplementary Fig. 24 | Summarized $qV_{on}/\sum E_g$ values for our tandem PeLEDs (red stars) compared to values reported perovskite-based tandem LEDs (see Extended Data Table 1 for details).

The sub-bandgap turn-on voltages ($V_{on}<\sum E_g/q$) likely originates from the radiative recombination of non-thermal-equilibrium band-edge carriers, whose population distribution complies with the Fermi-Dirac statistics under low forward bias[10].

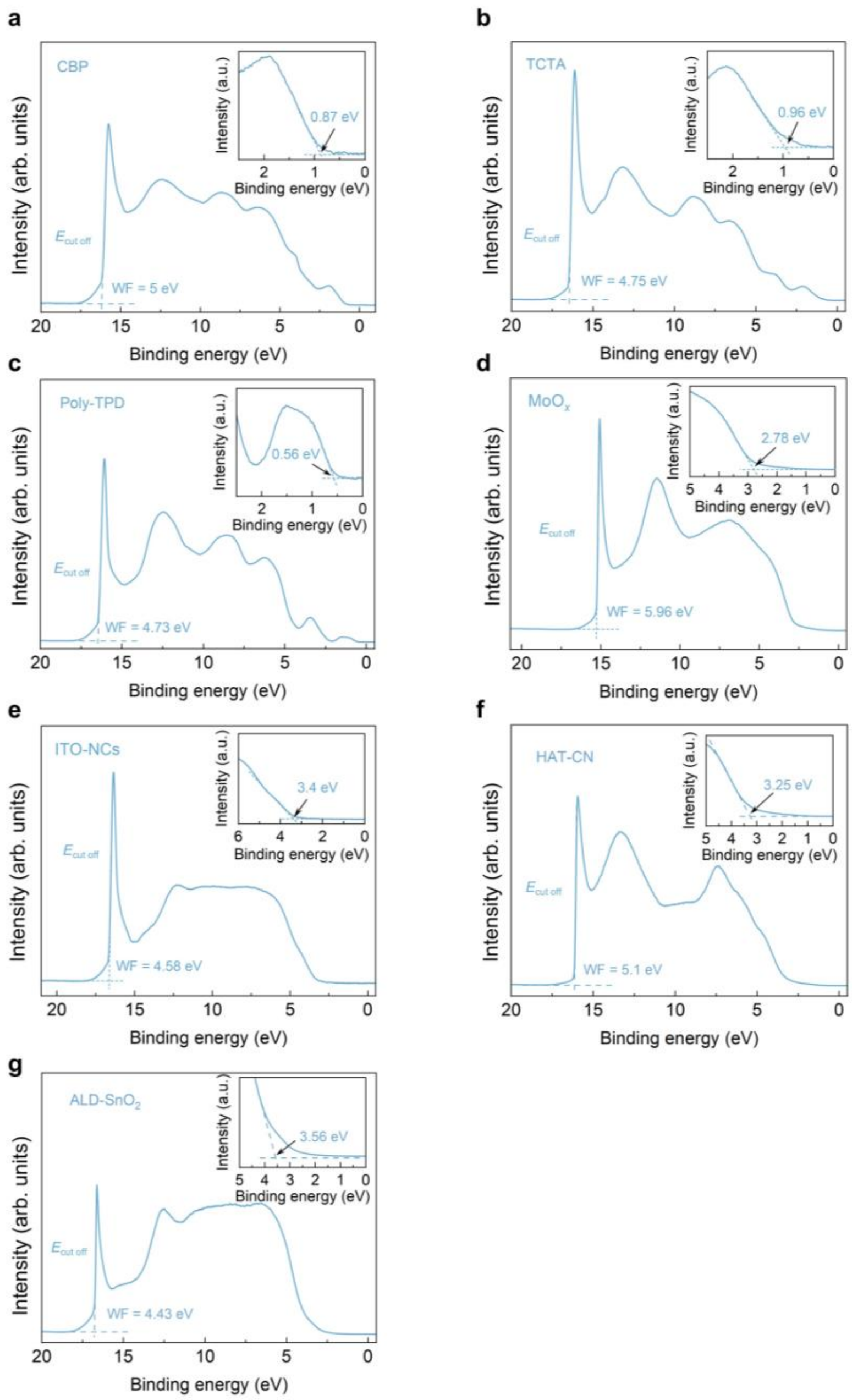

Supplementary Fig. 25 | UPS spectra of the functional layers. a, CBP. **b**, TCTA. **c**, Poly-TPD. **d**, MoO_x. **e**, ITO-NCs. **f**, HAT-CN. **g**, ALD-SnO_2. The inset shows the position of the valence band relative to the Fermi level edge (at 0 eV).

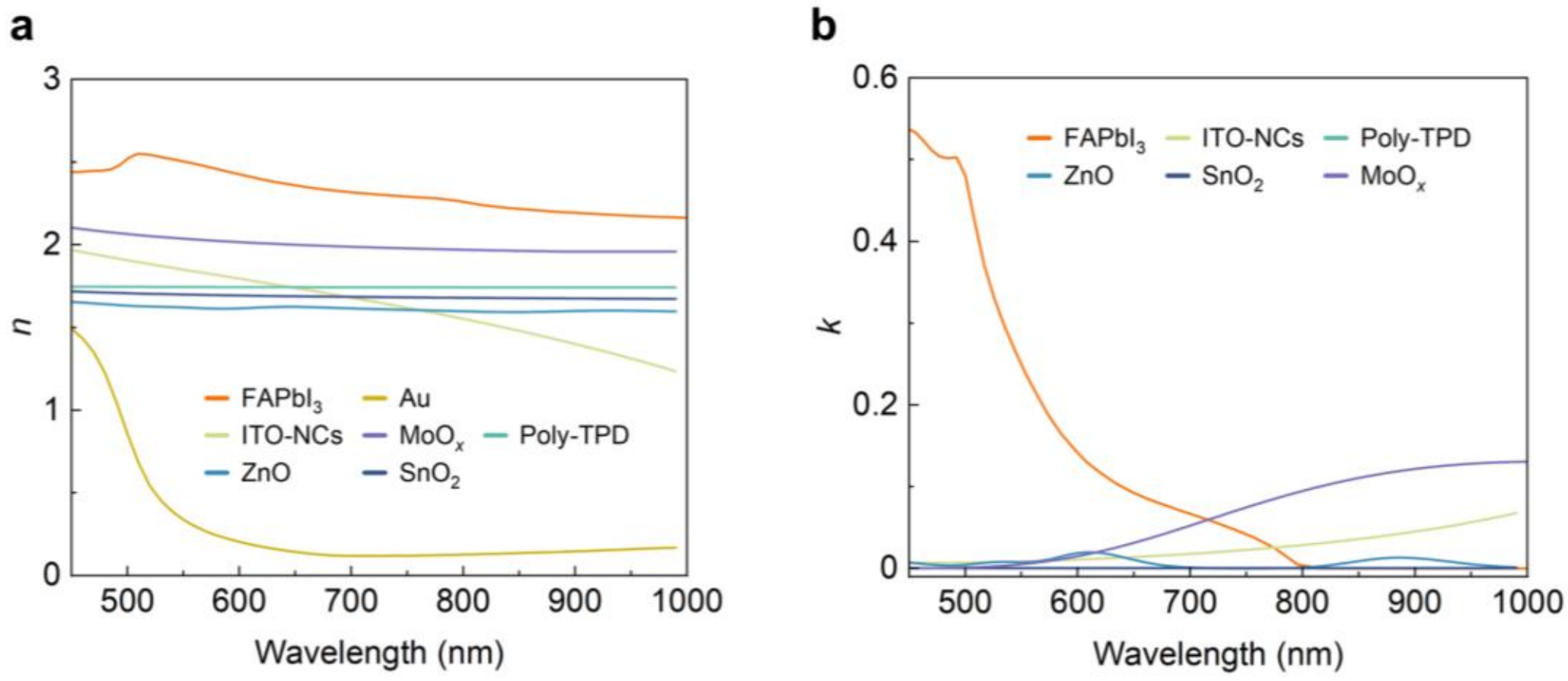

Supplementary Fig. 26 | Optical constants of the functional layers in our tandem devices.

All optical constants (*n*, *k*) of the layers were determined using an ellipsometer.

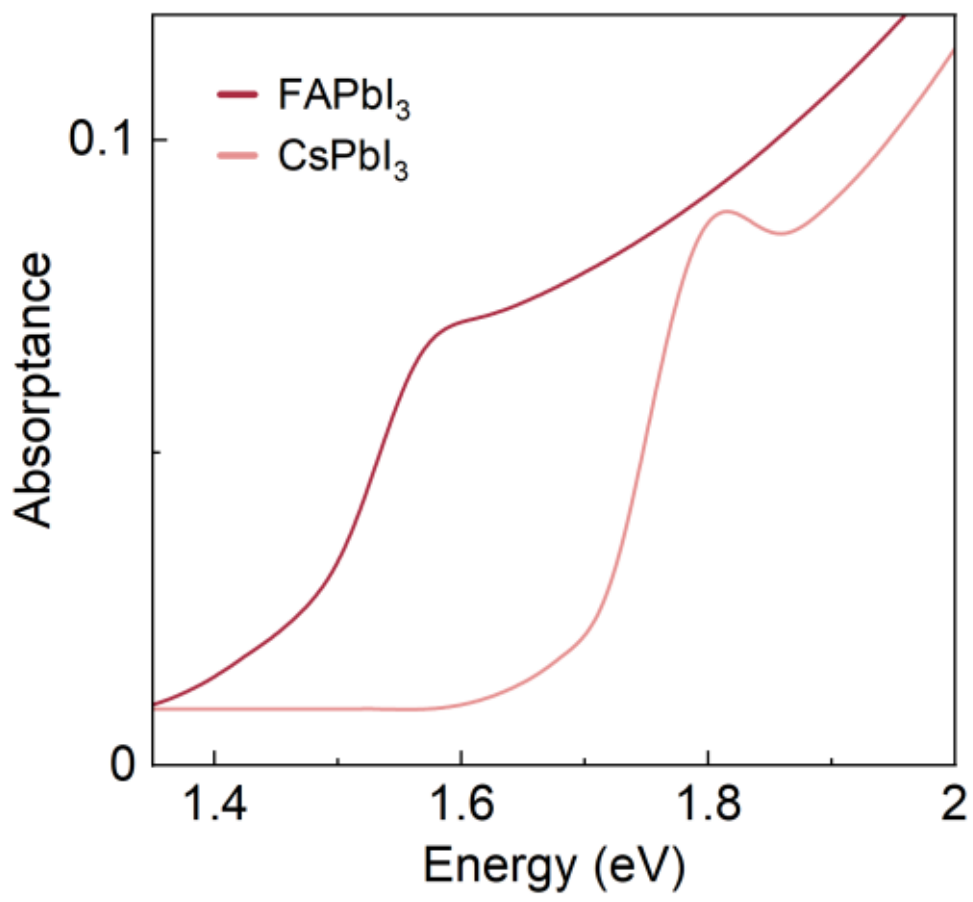

Supplementary Fig. 27 | UV-vis absorption spectra of $CsPbI_3$ and $FAPbI_3$.

Supplementary Note 1:

At elevated temperatures, thermal energy enables carriers to overcome the interfacial energy barrier, leading to a strong temperature dependence of the *J*-*V* curves. The natural logarithm of current density (ln(*J*)) scales linearly with the square root of voltage ($V^{1/2}$), as described by the Richardson-Schottky model[3]:

$$J = A^{*}T^{2}\exp\left[\frac{-(\phi_{B}-\sqrt{q^{3}V/4\pi\varepsilon_{0}\varepsilon_{r}d})}{k_{b}T}\right] \tag{1}$$

where A^{*} is the effective Richardson constant, k_b is the Boltzmann constant, *T* is the temperature, ϕ_b is the barrier height, *q* is the elementary charge, and *V* is the applied voltage, *d* is the barrier width, ε_r is the permittivity of the semiconductor, and ε_0 is the permittivity of the vacuum. Under zero-bias conditions (V = 0), the voltage-dependent term vanishes, simplifying the model to[11]:

$$\ln\left(\frac{J}{T^{2}}\right) = \ln(A^{*}) - \frac{\phi_{b}}{k_{B}T} \tag{2}$$

by plotting $\ln(J/T^2)$ versus 1000/*T*, the slope of the linear regime directly yields ϕ_b, which represents the intrinsic barrier height.

Supplementary Note 2:

To assess the free carrier concentration in CGL stacks, we prepared devices with a layer sequence ITO/HTL (30 nm)/MoO_x (8 nm)/Au. Forward bias refers to a positive bias of the ITO electrode with respect to the Au electrode.

Generally, the free carrier concentration in a capacitor can be determined based on the Mott-Shockley Equation[1,2], given as follows:

$$\left(\frac{1}{C^2}\right) = \frac{2(V_{bi} + V)}{e\varepsilon_s N_D} \tag{3}$$

where C denotes the capacitance per unit area, e is elementary charge, ε_s is dielectric constant, N_D is free carrier concentration, V is the applied voltage, and V_{bi} is the built-in voltage. The free carrier concentration can be obtained by fitting the slope of the $1/C^2$-V curve.

Supplementary Table 1 | Summary of emitter compositions and key performance merits of perovskite-organic (hybrid) and all-perovskite tandem LEDs.

Tandem Devices	Stacked Emitters	EQE (%)	Radiance (W sr^{-1} m^{-2})	V_{on} (V)	$qV_{on}/\sum E_g$	Voltage at 100 mA cm^{-2} (V)	Refs
Double-junction (Hybrid)	$FA_{0.9}GA_{0.1}PbBr_3$ QDs/ Ir(ppy)$_2$acac in CBP/TCTA	37	49†	5.0†	1.06†	16*	12
	$CsPbBr_3$/ CBP:Ir(ppy)$_2$(acac)	43.4	410†	5.0	1.04†	17*	13
	$CsPbBr_3$/CBP:B4PyMPM:4CzIPN	41.1	77†	5.4	1.14†	14*	14
	$CsPb(Br/Cl)_3$/CBP:PO01:Ir(mphmq)$_2$tmd	24.3	70†	5.4	1.13†	15*	15
Double-junction (PeLED)	$CsPbBr_3$ QDs/ $CsPb(Br/I)_3$ QDs	1.8	10†	6.4	1.46†	>15*	16
	$PEA_2(Cs_{1-x}EA_xPbBr_3)_2PbBr_4$ /$CsPbI_3$ QDs	8.5	9†	6.2	1.43†	>15*	17
	$PA_2CsPb_2I_7$/ $CsPb(Br/Cl)_3$ QDs	0.22	0.17	5.2	1.2†	9.6*	18
	$CsPbBr_3$/$CsPbBr_3$	35.4	160†	5	1.04†	10*	19
	$FAPbI_3$/$FAPbI_3$	45.5	62.6	3.5	1.13†	15*	7
	$FA_{1-x}Cs_xPbI_3$/$FAPbI_3$	38.1	140	3.2	1.0†	12.8*	
	**$CsPbI_3$/$FAPbI_3$**	**40.9**	**607**	**2.55**	**0.77**	**5.1**	**This work**
	**$FAPbI_3$/$FAPbI_3$**	**42.6**	**690**	**2.35**	**0.76**	**5.5**	
	**$FAPbI_3$/$FAPbI_3$**	**45.6**	**303**	**2.76**	**0.89**	**6.1**	
Multiple-junction (PeLED)	**$FAPbI_3$ (×3)**	**53.9**	**755**	**3.6**	**0.77**	**8.8**	**This work**
	**$CsPbI_3$/ $FA_{1x}Cs_xPbI_3$/$FAPbI_3$**	**45.7**	**511**	**4.0**	**0.8**	**9.6**	

**$FAPbI_3$ (×4)**	**58.2**	**392**	**5.0**	**0.8**	**15.8**
**$FAPbI_3$ (×5)**	**62.5**	**210**	**6.6**	**0.85**	**20.9**

†The values are estimated from the given data or figures in the reference papers.

*The values are estimated from the presented results, and a majority of the measured current densities for these tandem devices are below 100 mA cm^{-2}.

V_{on} denotes the voltage required to reach a radiance of 0.01 W sr^{-1} m^{-2} or a luminance of 1 cd m^{-2}, q is the elementary charge.

Supplementary References

1. Kleemann, H., Lüssem, B. & Leo, K. Controlled formation of charge depletion zones by molecular doping in organic pin-diodes and its description by the Mott-Schottky relation. *J. Appl. Phys.* **111**, (2012).
2. Yuan, J. *et al.* Investigation on the mechanism of charge generation in organic heterojunctions: analysis of I–V and C–V characteristics. *Org. Electron.* **88**, 105979 (2021).
3. Sarker, B. K. & Khondaker, S. I. Thermionic emission and tunneling at carbon nanotube–organic semiconductor interface. *ACS Nano* **6**, 4993–4999 (2012).
4. Burke, S. A., Topple, J. M. & Grütter, P. Molecular dewetting on insulators. *J. Phys. Condens. Matter* **21**, 423101 (2009).
5. Turak, A. Dewetting Stability of ITO Surfaces in Organic Optoelectronic Devices. in *Optoelectronics - Advanced Materials and Devices* (InTech, 2013).
6. Fenter, P., Schreiber, F., Bulović, V. & Forrest, S. R. Thermally induced failure mechanisms of organic light emitting device structures probed by X-ray specular reflectivity. *Chem. Phys. Lett.* **277**, 521–526 (1997).
7. Ke, Y. *et al.* High-performance tandem perovskite LEDs through interlayer photon recycling. *Nature* **649**, 53–58 (2026).
8. Zeng, J. *et al.* ZnO-based electron-transporting layers for perovskite light-emitting diodes: controlling the interfacial reactions. *J. Phys. Chem. Lett.* **13**, 694–703 (2022).
9. Xu, W. *et al.* Rational molecular passivation for high-performance perovskite light-emitting diodes. *Nat. Photonics* **13**, 418–424 (2019).
10. Lian, Y. *et al.* Ultralow-voltage operation of light-emitting diodes. *Nat. Commun.* **13**, (2022).
11. Aboelfotoh, M. O., Fröjdh, C. & Petersson, C. S. Schottky-barrier behavior of metals on n - and p -type 6 H − SiC. *Phys. Rev. B* **67**, 075312 (2003).
12. Lee, H.-D. *et al.* Valley-centre tandem perovskite light-emitting diodes. *Nat. Nanotechnol.* **19**, 624–631 (2024).
13. Kong, L. *et al.* Efficient and stable hybrid perovskite-organic light-emitting diodes with external quantum efficiency exceeding 40 per cent. *Light Sci. Appl.* **13**, 138 (2024).
14. Zhu, M. *et al.* Perovskite/organic hybrid tandem light-emitting diodes with an external quantum efficiency of over 40%. *J. Mater. Chem. C* **12**, 2623–2628 (2024).
15. Sun, S. *et al.* Highly efficient hybrid perovskite/organic tandem white light emitting-diodes with external quantum efficiency exceeding 20%. *Adv. Funct. Mater.* **33**, 1–8 (2023).
16. Wang, R. *et al.* Minimizing energy barrier in intermediate connection layer for monolithic tandem WPeLEDs with wide color gamut. *Adv. Funct. Mater.* **33**, 2215189 (2023).
17. Yan, Y. *et al.* High-efficiency tandem white perovskite light-emitting diodes by using an organic/inorganic intermediate connector. *Crystals* **12**, 1286 (2022).
18. Mao, J. *et al.* All-perovskite emission architecture for white light-emitting diodes. *ACS Nano* **12**, 10486–10492 (2018).
19. Geng, C. *et al.* Efficient white light-emitting diodes based on all-perovskite triple-junction tandems. *Nat. Photonics* **20**, 554–562 (2026).
