# Supplementary material for: High-performance multijunction perovskite LEDs with reduced interconnection loss
Source: Nat Commun. 2026 Jul 17;17:8781. doi: 10.1038/s41467-026-75756-5 (PMC13493991; doi:10.1038/s41467-026-75756-5)
Supplement: Supplementary file 2 [file 41467_2026_75756_MOESM2_ESM.pdf]

nature portfolio

Peer Review File

High-performance multijunction perovskite LEDs with reduced interconnection loss

Corresponding Author: Professor Feng Gao

This file contains all reviewer reports in order by version, followed by all author rebuttals in order by version.

Version 0:

Reviewer comments:

Reviewer #1

(Remarks to the Author)
This manuscript developed an important and practically relevant design for solution-processed high-efficiency and bright tandem perovskite LEDs (PeLEDs) with minimal performance loss of integrated subunits. The device results are impressive and represent a significant milestone in the research field, even compared with the two recent works in Nature and Nature Photonics. More importantly, the authors revealed the key to the ICL design for tandem PeLEDs, which generate and transport charge efficiently without degrading the underlying subunits. I do not fully agree with the previous reviewers' view that the present manuscript is merely incremental or lacking conceptual novelty. Instead, comparisons of the EQE alone do not constitute a fully fair evaluation of tandem LED performance. Actually, for practical LEDs, EQE, radiance, operating voltage, and scalability should be considered together, especially for multijunction architectures operating under realistic current densities. This work addresses a distinct and practically important bottleneck in tandem PeLEDs: how to minimize interconnection-related electrical loss while preserving compatibility and multijunction scalability of tandem devices using solution-processed perovskite emitters. I believe this aspect has not been sufficiently appreciated in some of the previous comments. Overall, this work offers a practical and well-characterized interconnection strategy for solution-processed tandem PeLEDs, which would definitely advance further developments of the new technology. I recommend acceptance of the manuscript for publication in Nature Communications. The manuscript is already well organized and clearly presented; most of the previous comments have also been properly addressed during the revision; there are only a few minor problems that may need further consideration.

1. A conclusion section summarizing key results and implications of the work may be necessary to further enhance the quality of the manuscript.

2. In Extended Data Figure 3, the authors demonstrate that adjusting the Poly-TPD thickness can yield a higher EQE exceeding 45% with reduced radiance as noted. Would the author provide some discussion on the performance trade-off? Also, I would suggest including these specific data in the Extended Data Table for a more comprehensive comparison.

3. The authors have provided a thorough investigation into the effects of material selection for each layer in the ICL and specific processing parameters on device performance. I am curious whether the ITO-NCs concentration also significantly affects the device performance.

4. The authors may need to provide more detailed ALD parameters, including the Sn precursor, number of deposition cycles, purge times, etc., for reference.

Reviewer #2

(Remarks to the Author)
The manuscript by Zhao et al. presents a clear and timely advance in multijunction perovskite light-emitting diodes with a well-designed structure, comprehensive characterizations of films and devices. The pursuit of tandem devices is a well-known strategy for performance improvements, especially in terms of brightness and lifetime, of LEDs. It is also well-known that the interconnection layer (ICL) is key yet challenging for solution-processed all-perovskite tandem LEDs. As demonstrated by previous studies and also in the present study, conventional designs from OLEDs or QD-LEDs are often problematic for tandem perovskite LEDs. The developed hierarchical composite ICL shows excellent optoelectronic

properties and is robust under harsh processing conditions of the tandem LEDs. The demonstrated double-junction devices with a peak EQE of 42.6% and a radiance of 690 W sr^{-1} m^{-2}, systematically combine the advantages of high efficiency, brightness, and prolonged lifetime of tandem devices. Moreover, the authors also go further by applying the ICL design to triple-, four-, and five-junction devices, achieving multijunction PeLEDs that maintain sub-bandgap turn-on voltages and impressively high EQEs exceeding 50-60%. The reviewer considers that this work establishes a systematic electrical-interconnection design strategy for all-perovskite tandem LEDs. Therefore, I recommend publication of the manuscript in Nature Communications after addressing the following issues.

1. The authors may need to consider updating the Extended Data Table 1 by adding some of the tandem PeLED metrics published recently (e.g., tandem devices from Nature Photonics) for a more complete comparison.

2. In Supplementary Fig. 8, the authors demonstrate the effect of prolonged thermal annealing on the EQE of single-junction and double-junction FAPbI3-based PeLEDs, which helps to understand the thermal degradation behavior of the perovskite subunits during multijunction fabrication. The reviewer considers that a similar analysis may also be useful for the CsPbI3-based devices shown in Fig. 3, including both the single-junction CsPbI3 reference and the double-junction CsPbI3/FAPbI3 tandem devices.

3. A more detailed discussion between the present study and recent works on tandem perovskite LEDs may be helpful to clearly differentiate the technical focuses of the work. For example, the authors may consider providing some performance comparisons between their devices and the recently published double-junction FAPbI3/FAPbI3 tandem LEDs in Nature.

Reviewer #3

(Remarks to the Author)
In this manuscript, the authors report a hierarchically structured interconnection layer (ICL) for tandem and multijunction perovskite light-emitting diodes (PeLEDs). The proposed ICL integrates a Poly-TPD/MoOx charge-generation junction, an ITO nanocrystal (ITO-NC) bridging layer, and an ALD-SnO_2 protection/electron-transport layer. This design addresses several critical challenges in solution-processed tandem PeLEDs, including voltage loss, interfacial charge transport, solvent orthogonality, and compatibility with diverse perovskite emitters. The device performance is very impressive, particularly for the double-junction $FAPbI_3$/$FAPbI_3$ PeLEDs exhibiting a peak EQE of 42.6%, high radiance, and a low operating voltage. Furthermore, the successful demonstration of triple-, four-, and five-junction devices underscores the scalability of the proposed interconnection strategy. Comprehensive characterization and simulations robustly validate the underlying mechanisms.
Overall, the manuscript is well-organized, and the main conclusions are supported by a comprehensive set of experimental results. I recommend publication subject to minor revisions. The following points should be addressed to enhance the clarity, rigor, and reproducibility of the work:

Q1. The authors highlight sub-bandgap turn-on voltages ($V_{on} < \sum E_g/q$) for their tandem PeLEDs. However, given that Von is defined at a very low radiance threshold (0.01 W sr^{-1} m^{-2}), this observation likely reflects emission from band-tail states or defect levels rather than band-to-band recombination. I suggest the authors briefly clarify the physical origin of this phenomenon in the manuscript.
Q2. In Supplementary Fig. 4, SEM images of HAT-CN before and after annealing reveal thermally induced morphological changes in this common organic bridging layer. To better substantiate the claimed superior stability of the ITO-NC-based bridging layer, the authors should provide corresponding SEM or AFM comparisons of the ITO-NCs (or ITO-NCs/ALD-SnO_2) before and after the same thermal treatment. This would offer a more direct comparison with HAT-CN and strengthen the rationale for selecting ITO-NCs as the bridging layer.
Q3. The authors attribute the constrained maximum radiance in multijunction devices to Joule heating, as evidenced by the thermal imaging and simulations in Supplementary Fig. 14. Nevertheless, the detailed explanation in the main text is limited. The manuscript would be strengthened by including temperature characteristics for the four- and five-junction PeLEDs, along with a brief discussion elucidating the origin of the radiance decline.
Q4. ALD-SnO_2 is a key component of the ICL, providing both solvent protection and electron transport. While the Methods section only states that the ALD-SnO_2 was deposited at 80 °C, additional deposition details and parameters are needed to ensure reproducibility.
Q5. Several minor language errors and typos should be corrected. For instance:
•On Page 3, “and can substantially prolonged operational lifetime” should be “and can substantially prolong operational lifetime.”
•On Page 6, “As an key component in tandem LEDs” should be “As a key component in tandem LEDs.” The authors should proofread the manuscript to correct those similar issues.

Version 1:

Reviewer comments:

Reviewer #1

(Remarks to the Author)
The authors have addressed all my points. I recommend its publication without further revisions.

Reviewer #2

(Remarks to the Author)
All of my concerns have been adequately addressed, and I am pleased to recommend this manuscript for publication.

Reviewer #3

(Remarks to the Author)
The authors have addressed all of my previous concerns. I would suggest the publication as it is now.

Open Access This Peer Review File is licensed under a Creative Commons Attribution 4.0 International License, which permits use, sharing, adaptation, distribution and reproduction in any medium or format, as long as you give appropriate credit to the original author(s) and the source, provide a link to the Creative Commons license, and indicate if changes were made.
In cases where reviewers are anonymous, credit should be given to 'Anonymous Referee' and the source.
The images or other third party material in this Peer Review File are included in the article's Creative Commons license, unless indicated otherwise in a credit line to the material. If material is not included in the article's Creative Commons license and your intended use is not permitted by statutory regulation or exceeds the permitted use, you will need to obtain permission directly from the copyright holder.
To view a copy of this license, visit https://creativecommons.org/licenses/by/4.0/

Referees' comments:

Reviewer #1 (Remarks to the Author):

This manuscript developed an important and practically relevant design for solution-processed high-efficiency and bright tandem perovskite LEDs (PeLEDs) with minimal performance loss of integrated subunits. The device results are impressive and represent a significant milestone in the research field, even compared with the two recent works in Nature and Nature Photonics. More importantly, the authors revealed the key to the ICL design for tandem PeLEDs, which generate and transport charge efficiently without degrading the underlying subunits. I do not fully agree with the previous reviewers' view that the present manuscript is merely incremental or lacking conceptual novelty. Instead, comparisons of the EQE alone do not constitute a fully fair evaluation of tandem LED performance. Actually, for practical LEDs, EQE, radiance, operating voltage, and scalability should be considered together, especially for multijunction architectures operating under realistic current densities. This work addresses a distinct and practically important bottleneck in tandem PeLEDs: how to minimize interconnection-related electrical loss while preserving compatibility and multijunction scalability of tandem devices using solution-processed perovskite emitters. I believe this aspect has not been sufficiently appreciated in some of the previous comments. Overall, this work offers a practical and well-characterized interconnection strategy for solution-processed tandem PeLEDs, which would definitely advance further developments of the new technology. I recommend acceptance of the manuscript for publication in Nature Communications. The manuscript is already well organized and clearly presented; most of the previous comments have also been properly addressed during the revision; there are only a few minor problems that may need further consideration.

R1Q1. A conclusion section summarizing key results and implications of the work may be necessary to further enhance the quality of the manuscript.

Response: We thank the reviewer for the helpful suggestion. Following the reviewer's suggestion, we have now added a conclusion at the end of the main text (before the methods section). The conclusion succinctly summarizes the core contributions of this

work, including the low-loss ICL design, the well-balanced high EQE and high radiance achieved in double-junction devices, and the excellent scalability extending to five-junction PeLEDs.

Revision: We have now added the related description in the main text of the revised version. "In summary, we have developed a hierarchically structured composite ICL that ensures efficient charge generation at low bias, eliminates interfacial transport barriers, and provides exceptional solvent and thermal robustness while enabling damage-free sequential solution processing of multiple perovskite emitter layers. Using this ICL, we demonstrate double-junction PeLEDs that combine a peak EQE of over 40% (maximum 45.6%), a maximum radiance of 690 W sr^{-1} m^{-2}, and a low turn-on voltage of 2.35 V. We further validate the generality and scalability of our approach by extending it to triple-, four-, and five-junction PeLEDs consisting of perovskite emitters with same and distinct emissions, achieving peak EQEs of 53.9%, 58.2%, and 62.5% with consistently low turn-on voltages. Our results suggest that for multijunction PeLEDs, advanced strategies such as interface engineering or thermal management optimization would be imperative to mitigate performance degradation caused by repetitive thermal annealing and severe Joule heating. We anticipate that the rational low-loss ICL design and strategies for subunit modulations would further advance the development of emerging tandem PeLEDs for various advanced applications."

R1Q2. In Extended Data Figure 3, the authors demonstrate that adjusting the Poly-TPD thickness can yield a higher EQE exceeding 45% with reduced radiance as noted. Would the author provide some discussion on the performance trade-off? Also, I would suggest including these specific data in the Extended Data Table for a more comprehensive comparison.

Response: We thank the reviewer for this important comment.

We fully agree that the performance trade-off between EQE and radiance induced by varying Poly-TPD thickness merits in-depth discussion. In the original manuscript, we briefly stated that increasing Poly-TPD thickness enables a peak EQE over 45%, yet it causes severe efficiency roll-off and notably decreases the maximum radiance to around

300 W sr^{-1} m^{-2}. We consider the elevated peak EQE may be relevant to enhanced light outcoupling with the thicker hole-transport layers. Nevertheless, such thick films inevitably aggravate Joule heating under high current densities and accelerate efficiency roll-off behavior. Meanwhile, excessive thermal accumulation further constrains the output brightness, thus lowering the maximum radiance.

Following the reviewer's suggestion, we have supplemented a detailed discussion on this inherent trade-off in the revised manuscript with explicit reference to **Extended Data Fig. 3**. Specifically, we clarify that thicker Poly-TPD facilitates photon recycling at low current levels and accordingly boosts peak EQE, but it also raises voltage drop and thermal dissipation under high-current operation, which significantly limits the maximum radiance. Consequently, we adopt the optimized Poly-TPD thickness corresponding to a solution concentration of 14 mg mL^{-1}, realizing an optimal balance between peak EQE and maximum radiance.

Revision: We have added the key performance metrics for the devices with different Poly-TPD thicknesses to Extended Data Table 1 and included the corresponding description in the caption of Extended Data Fig. 3.

R1Q3. The authors have provided a thorough investigation into the effects of material selection for each layer in the ICL and specific processing parameters on device performance. I am curious whether the ITO-NCs concentration also significantly affects the device performance.

Response: We thank the reviewer for the important comment. During our systematic experimental optimization, we have thoroughly investigated the influence of ITO-NCs concentration on device performance. As shown in **Supplementary Fig. 7**, we systematically optimized the effect of ITO-NCs film thickness (30 nm, 60 nm, and 100 nm) on device performance and found that the best device performance was achieved at a thickness of 60 nm, which is the value adopted in this work, corresponding to an ITO-NCs concentration of 5 wt%. For clearer comparison, we have also supplemented the concentrations corresponding to the other two thicknesses (3 wt% and 8 wt%) in **Supplementary Fig. 7**. We sincerely appreciate the reviewer's valuable suggestion to

enrich this experimental detail.

Revision: We have added the concentration of ITO-NCs corresponding to different film thicknesses in Supplementary Fig. 7 in the revised version of the Supplementary Information.

R1Q4. The authors may need to provide more detailed ALD parameters, including the Sn precursor, number of deposition cycles, purge times, etc., for reference.

Response: We apologize for missing detailed ALD parameters in the original manuscript. We have now provided a complete description of the ALD process for SnO_2 deposition in the methods section as follows:

Chamber pressure: 0.1 Torr

Substrate temperature: 80 °C

Precursors: tetrakis(dimethylamido)tin(IV) (TDMASn, maintained at 70 °C during the ALD process), deionized water

One deposition cycle: TDMASn pulse (0.18 s) - N_2 purge (5 s) - H_2O pulse (0.03 s) -N_2 purge (5 s)

Total cycles for 20 nm: 170 cycles.

Revision: We have now added the ALD-SnO_2 fabrication details in the methods section of the revised version.

Reviewer #2 (Remarks to the Author):

The manuscript by Zhao et al. presents a clear and timely advance in multijunction perovskite light-emitting diodes with a well-designed structure, comprehensive characterizations of films and devices. The pursuit of tandem devices is a well-known strategy for performance improvements, especially in terms of brightness and lifetime, of LEDs. It is also well-known that the interconnection layer (ICL) is key yet challenging for solution-processed all-perovskite tandem LEDs. As demonstrated by previous studies and also in the present study, conventional designs from OLEDs or QD-LEDs are often problematic for tandem perovskite LEDs. The developed hierarchical composite ICL shows excellent optoelectronic properties and is robust

under harsh processing conditions of the tandem LEDs. The demonstrated double-junction devices with a peak EQE of 42.6% and a radiance of 690 W sr^{-1} m^{-2}, systematically combine the advantages of high efficiency, brightness, and prolonged lifetime of tandem devices. Moreover, the authors also go further by applying the ICL design to triple-, four-, and five-junction devices, achieving multijunction PeLEDs that maintain sub-bandgap turn-on voltages and impressively high EQEs exceeding 50-60%. The reviewer considers that this work establishes a systematic electrical-interconnection design strategy for all-perovskite tandem LEDs. Therefore, I recommend publication of the manuscript in Nature Communications after addressing the following issues.

R2Q1. The authors may need to consider updating the Extended Data Table 1 by adding some of the tandem PeLED metrics published recently (e.g., tandem devices from Nature Photonics) for a more complete comparison.

Response: We thank the reviewer for the helpful suggestion. Following reviewer's suggestion, **Extended Data Table 1** has been updated with recent tandem PeLED metrics, including the tandem devices from Nature Photonics, for a more comprehensive comparison.

Revision: We have now updated Extended Data Table 1.

R2Q2. In Supplementary Fig. 8, the authors demonstrate the effect of prolonged thermal annealing on the EQE of single-junction and double-junction $FAPbI_3$-based PeLEDs, which helps to understand the thermal degradation behavior of the perovskite subunits during multijunction fabrication. The reviewer considers that a similar analysis may also be useful for the $CsPbI_3$-based devices shown in Fig. 3, including both the single-junction $CsPbI_3$ reference and the double-junction $CsPbI_3$/$FAPbI_3$ tandem devices.

Response: We thank the reviewer for the comment. We fully agree that more comprehensive data on the thermal degradation analysis would provide useful guidance for the fabrication of multi-junction PeLEDs. Following this suggestion, we carried out

additional thermal annealing experiments on single-junction $CsPbI_3$ PeLEDs (identical to the reference device in **Fig. 3**) and on double-junction $CsPbI_3/FAPbI_3$ tandem devices. As shown in **Fig. R1**, the single-junction $CsPbI_3$ PeLED degrades after thermal treatment at 100 ℃ for more than 20 minutes but does exhibit better thermal stability compared with that of $FAPbI_3$ subunits. Specifically, the finished $CsPbI_3$ single-junction device retains approximately 86% of its initial EQE after 30 minutes of thermal annealing at 100 ℃ (with the Poly-TPD coating), whereas the EQE retention of the $FAPbI_3$ device over the same duration is significantly lower. Since both tandem devices share the same top $FAPbI_3$ subunit, the performance evolution of the $FAPbI_3/FAPbI_3$ and $CsPbI_3/FAPbI_3$ devices is identical and highly relevant with the prolonged annealing of the $FAPbI_3$ layer. However, we do notice that the tandem $CsPbI_3/FAPbI_3$ devices show less obvious performance decay with an increased annealing time after the optimal condition (7 min), which is consistent with the improved thermal stability of the bottom $CsPbI_3$ subunit.

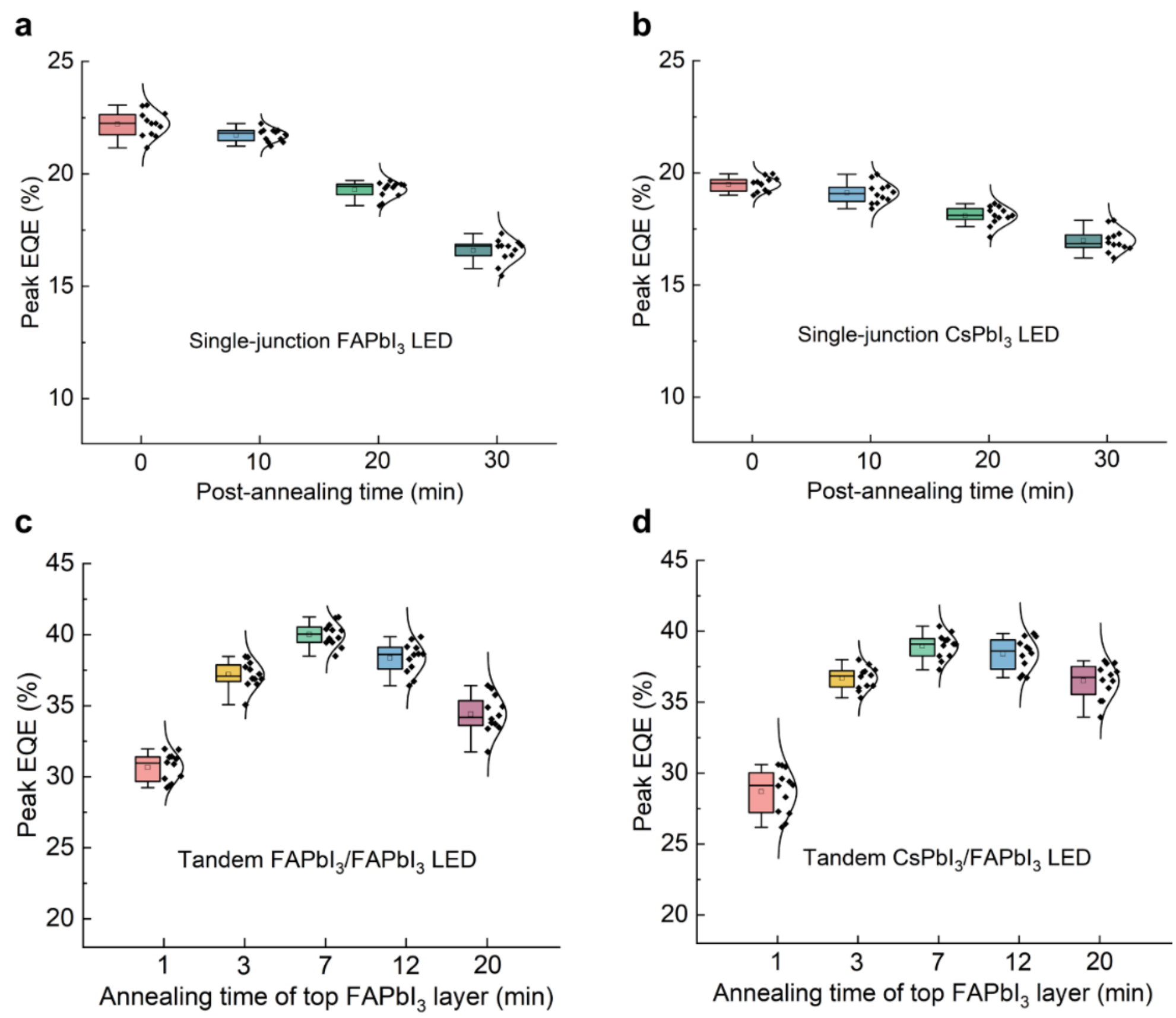

Fig. R1. Thermal effects of single-junction and tandem perovskite LEDs. a, b, Peak EQEs of single-junction $FAPbI_3$ (a) and $CsPbI_3$ LEDs (b) at different post-annealing

time. All annealing processes were conducted at 100 °C after depositing Poly-TPD. c, d, Peak EQEs of tandem $FAPbI_3$/$FAPbI_3$ (c) and $CsPbI_3$/$FAPbI_3$ (d) LEDs with different annealing time for top $FAPbI_3$ emissive layer.

Revision: We have now updated Supplementary Fig. 8 and added the related description in Supplementary Information of the revised version.

R2Q3. A more detailed discussion between the present study and recent works on tandem perovskite LEDs may be helpful to clearly differentiate the technical focuses of the work. For example, the authors may consider providing some performance comparisons between their devices and the recently published double-junction $FAPbI_3$/$FAPbI_3$ tandem LEDs in Nature.

Response: We thank the reviewer for this valuable suggestion. We fully agree that a clear differentiation of our work from concurrent high-performance tandem PeLED studies (Nature paper by Ke et al., 2026)[1] will strengthen the technical focus and advancement of our work. We provide a comprehensive comparison as below:

1. Distinct technical bottleneck.

Early tandem PeLEDs were severely limited by interconnection design and the extreme sensitivity of perovskite films to trap densities, often resulting in EQEs below 9% due to interfacial damage during sequential solution processing. Recent breakthroughs have taken different paths to circumvent these issues. The Nature work successfully mitigated interfacial damage by employing interlayer photon recycling combined with an ALD-SnO_2 buffer layer, achieving high EQEs.

In contrast, our manuscript is centered on a problem that becomes decisive for scalable, solution-processed multijunction PeLEDs at practical current densities, electrical interconnection loss across the interconnecting layer. We develop a function-integrated composite ICL, enabling low turn-on voltage, low driving voltage at high current density, and scalable stacking to higher junction numbers.

2. Performance comparison.

The reviewer notes that *Nature* paper reports a slightly higher peak EQE of 45.5% for a $FAPbI_3$/$FAPbI_3$ tandem structure. We point out that engineering the thicknesses of the

individual functional layers within the ICL could induce some benefits in improving the light outcoupling. However, such optimization comes at the expense of compromising other performance metrics, including driving voltage and radiance, a trade-off that we have consistently observed throughout our study. Therefore, the device reported herein represents an optimal balance we have achieved across all key performance parameters**.**

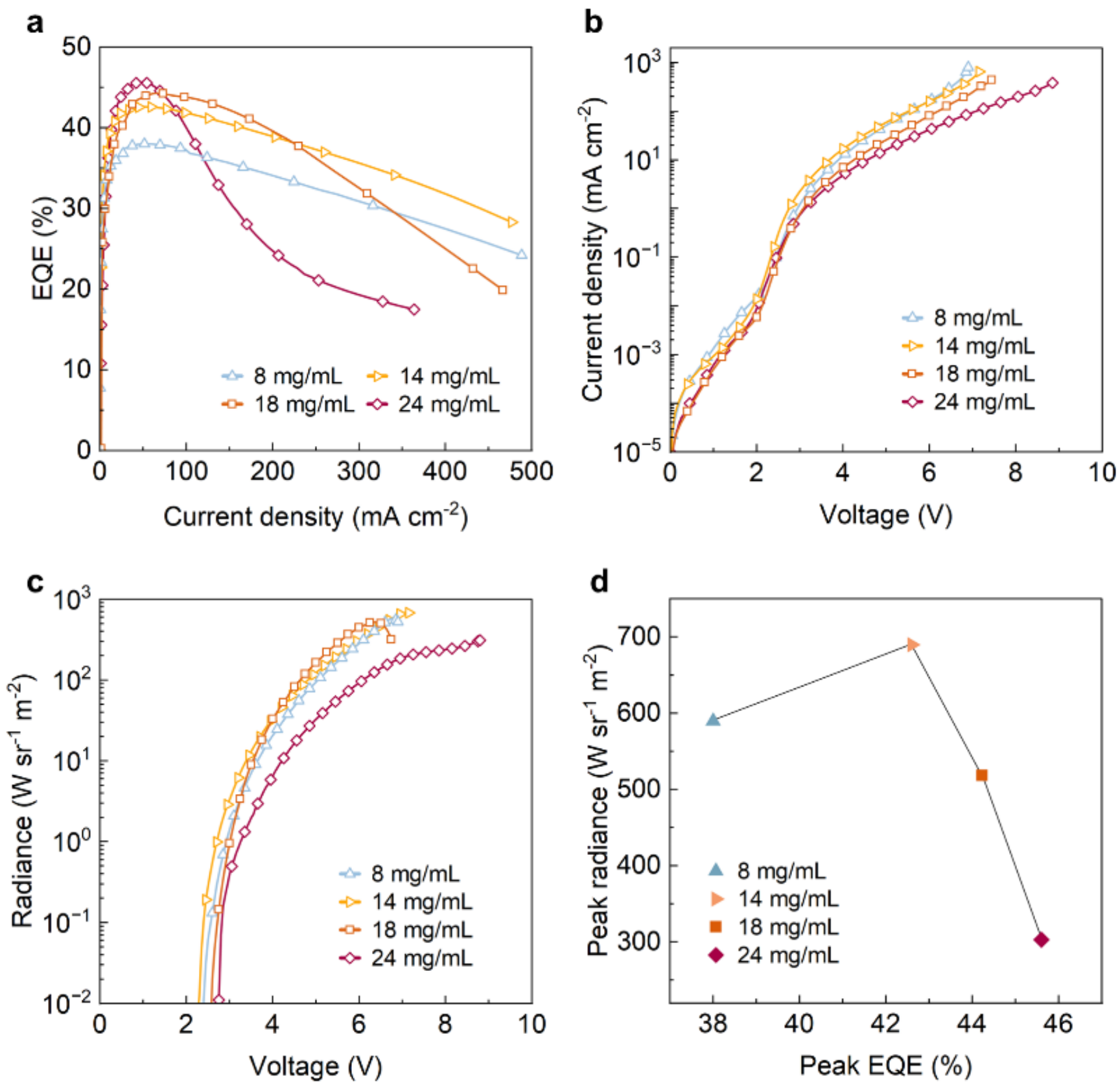

Fig. R2 | FAPbI$_3$/FAPbI$_3$ PeLEDs with varied thicknesses of Poly-TPD. **a-c**, EQE-*J* (**a**), *R*-*V* (**b**), and *J*-*V* (**c**) curves. **d,** The influence of the thickness of Poly-TPD on peak radiance and EQE. The thickness of Poly-TPD was simply controlled by tuning the solution concentration.

More importantly, our tandem devices operate in a substantially higher-brightness regime, delivering peak radiance values nearly an order of magnitude higher than those reported in Nature paper under comparable conditions. As shown in **Fig. R3** (using source data from Nature paper), our device delivers current density and radiance that are more than two orders of magnitude higher under identical driving voltage. It

demonstrates a different operating regime and a notable advance enabled by our low-loss electrical interconnection design.

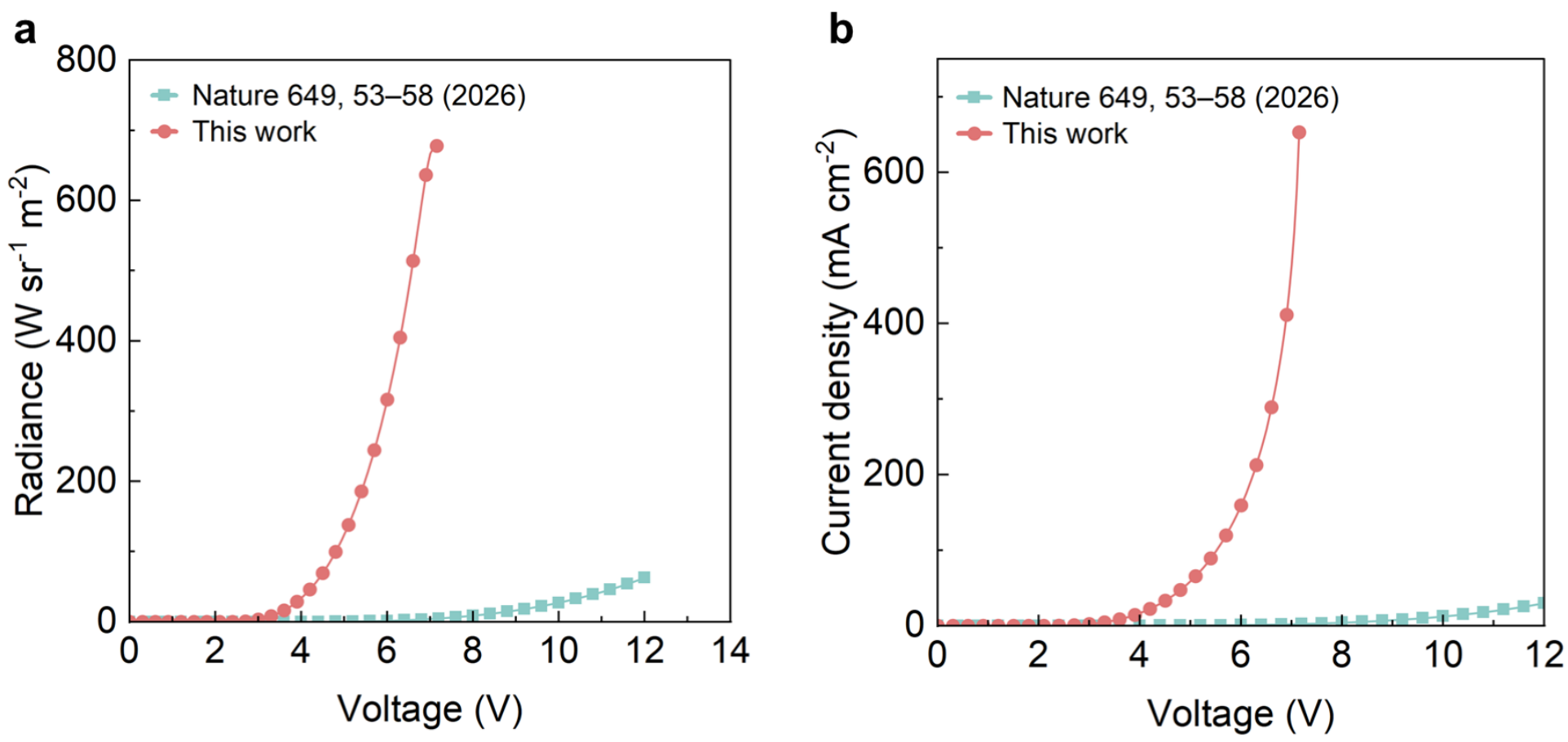

Fig. R3 | *R-V* and *J-V* curves of double-junction $FAPbI_3/FAPbI_3$ PeLEDs. The comparison curve is plotted using the source data provided in the reference.

3. Scalability beyond double-junction devices.

We have further extended our ICL design to multijunction devices, achieving EQEs of 58.2% and 62.5% for four- and five-junction PeLEDs, respectively. Moreover, the multijunction devices demonstrate turn-on voltages remaining below the summed bandgap limit and voltage penalties per ICL is as low as ~0.2-0.6 V. This scalability from double- to five-junctions with minimal performance loss demonstrates that our solution-process-compatible ICL can deliver minimal interconnection loss and preserve high performance as the number of junctions increases.

4. ICL robustness enabled by the ITO-NCs/ALD-SnO_2 nano-heterointerface.

Rather than simply adopting mature ICL designs from tandem OLEDs with an added ALD-SnO_2 protective layer, we designed our ICL with exceptional mechanical and chemical robustness by implementing a bulk ITO-NCs/ALD-SnO_2 heterointerface architecture. This robust ICL serves as a key factor enabling our devices to sustain significantly higher current densities and accommodate an increased number of junctions without significantly compromising electrical performance.

Revision: We have now added the related description in the main text of the revised version.

Reviewer #3 (Remarks to the Author):

In this manuscript, the authors report a hierarchically structured interconnection layer (ICL) for tandem and multijunction perovskite light-emitting diodes (PeLEDs). The proposed ICL integrates a Poly-TPD/MoO_x charge-generation junction, an ITO nanocrystal (ITO-NC) bridging layer, and an ALD-SnO_2 protection/electron-transport layer. This design addresses several critical challenges in solution-processed tandem PeLEDs, including voltage loss, interfacial charge transport, solvent orthogonality, and compatibility with diverse perovskite emitters. The device performance is very impressive, particularly for the double-junction $FAPbI_3$/$FAPbI_3$ PeLEDs exhibiting a peak EQE of 42.6%, high radiance, and a low operating voltage. Furthermore, the successful demonstration of triple-, four-, and five-junction devices underscores the scalability of the proposed interconnection strategy. Comprehensive characterization and simulations robustly validate the underlying mechanisms.

Overall, the manuscript is well-organized, and the main conclusions are supported by a comprehensive set of experimental results. I recommend publication subject to minor revisions. The following points should be addressed to enhance the clarity, rigor, and reproducibility of the work:

R3Q1. The authors highlight sub-bandgap turn-on voltages ($V_{on}<\sum E_g/q$) for their tandem PeLEDs. However, given that V_{on} is defined at a very low radiance threshold (0.01 W sr^{-1} m^{-2}), this observation likely reflects emission from band-tail states or defect levels rather than band-to-band recombination. I suggest the authors briefly clarify the physical origin of this phenomenon in the manuscript.

Response: We greatly appreciate the reviewer for pointing out this crucial issue. The reviewer proposes that the sub-bandgap turn-on voltages of our tandem PeLEDs, determined at the low radiance threshold of 0.01 W sr^{-1} m^{-2}, may stem from band-tail states or defect levels rather than intrinsic band-to-band recombination. We fully recognize the necessity to clarify the underlying physical mechanism of this phenomenon.

In fact, cumulative research findings, particularly the representative work reported by

Lian et al. in Nature Communications (2022)[2], have verified that sub-bandgap EL is a universal characteristic of various light-emitting diodes, covering perovskite, organic, quantum dot and III-V based devices. In their study, detectable EL signals were achieved at driving voltages as low as 36-60% of the corresponding semiconductor bandgap, delivering an apparent photon energy gain of 0.6-1.4 eV. Combining high-sensitivity photon counting technology and bandgap-edge cutoff bandpass filters, they further confirmed that such low-voltage luminescence does not derive from sub-bandgap defect or band-tail states. Instead, the ultralow-voltage EL emission originates from the radiative recombination of non-thermal-equilibrium band-edge carriers, whose population distribution complies with the Fermi-Dirac statistics under low forward bias. This mechanism is also solidly validated by their temperature-dependent characterization and device simulation results.

In line with this well-established universal mechanism, our tandem PeLEDs likewise present typical sub-bandgap turn-on behavior. However, we clarify that the turn-on threshold of 0.01 W sr^{-1} m^{-2} used in our work is not an arbitrarily low value. For a green emitter (530-560 nm), this radiance corresponds to a luminance of approximately 5-7 cd m^{-2}, which is a clearly perceptible brightness and well above the noise floor of our calibrated measurement system. At this emission level, the device is already operating in a regime where band-to-band recombination dominates, and contributions from band-tail or defect states are expected to be negligible.

We also note that the choice of turn-on threshold varies across the literature. For example, the recent tandem PeLED study[1] published in *Nature* defines turn-on voltage at an even lower radiance threshold of 4×10^{-3} W sr^{-1} m^{-2}. Our threshold is therefore comparatively conservative, further supporting that the observed sub-bandgap turn-on voltages reflect genuine low-loss electrical interconnection rather than sub-bandgap defect emission. Accordingly, the observed sub-bandgap turn-on feature in our devices is predominantly attributed to band-to-band recombination of intrinsic band-edge carriers, rather than luminescence mediated by defect states.

Revision: We have now added the related references in the revised version and provided the related description in the main text of the revised version.

R3Q2. In Supplementary Fig. 4, SEM images of HAT-CN before and after annealing reveal thermally induced morphological changes in this common organic bridging layer. To better substantiate the claimed superior stability of the ITO-NC-based bridging layer, the authors should provide corresponding SEM or AFM comparisons of the ITO-NCs (or ITO-NCs/ALD-SnO_2) before and after the same thermal treatment. This would offer a more direct comparison with HAT-CN and strengthen the rationale for selecting ITO-NCs as the bridging layer.

Response: We thank the reviewer for this important suggestion. Following the reviewer's suggestion, we have further conducted identical thermal treatment on the prepared ITO-NC based interlayer and characterized its surface morphology via SEM. As shown in **Fig. R4**, no obvious morphological variation can be observed for ITO-NCs/ALD-SnO_2 before and after annealing. Compared with HAT-CN that undergoes distinct structural deterioration under thermal stimulation, ITO-NCs/ALD-SnO_2 materials exhibit superior thermal morphological stability. This stable structural feature further verifies the great superiority of ITO-NCs as high-performance bridging layers for multijunction PeLEDs.

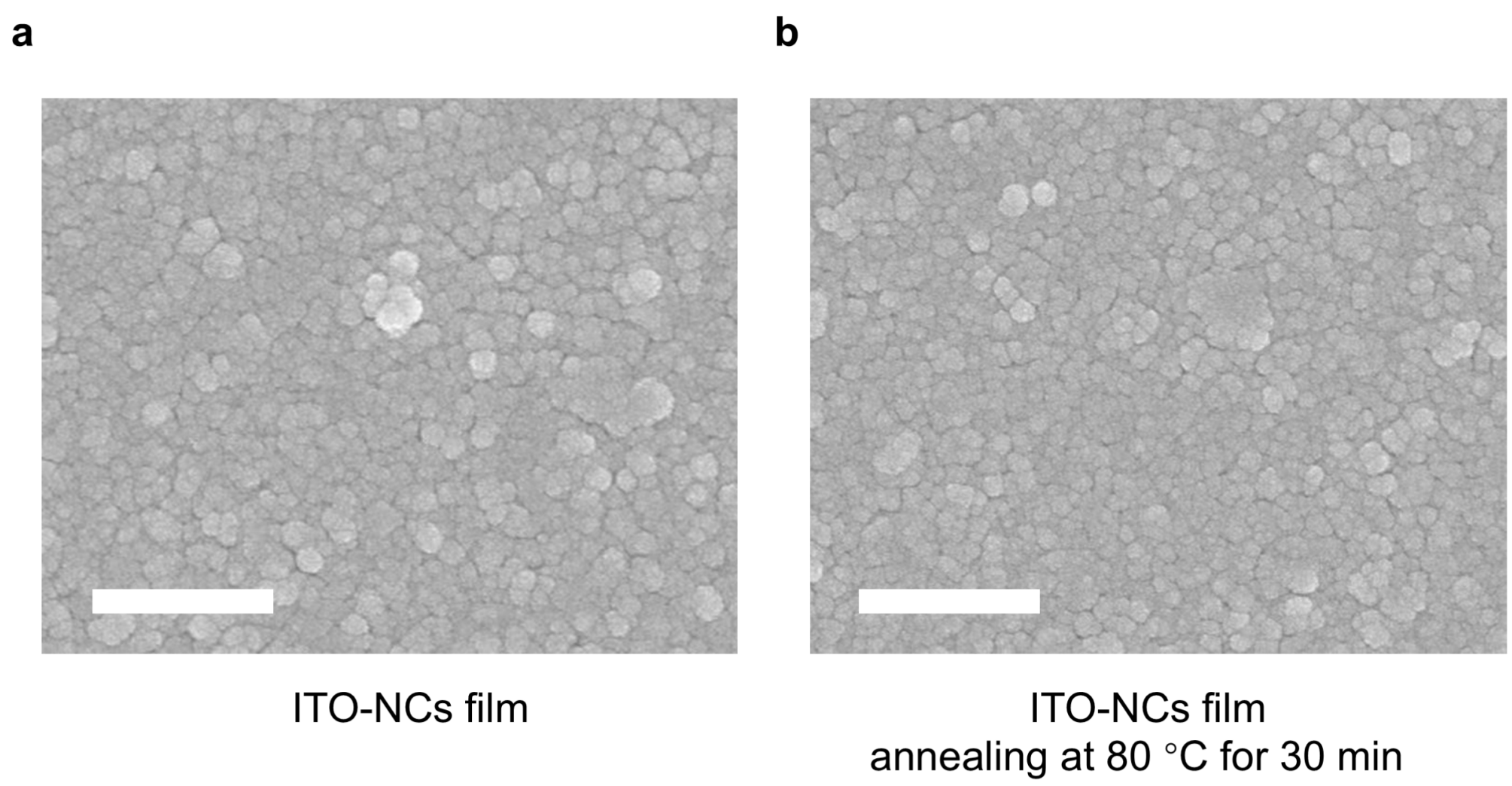

Fig. R4 | Thermal-induced morphology changes of HAT-CN and ITO-NCs/ALD-SnO_2. a,**b**, Scanning electron microscopy images of HAT-CN film before (**a**) and after (**b**) annealing at 80 °C for 30 min. Scale bar, 500 nm. **c**,**d**, Scanning electron microscopy

images of ITO-NCs/ALD-SnO_2 film before (**c**) and after (**d**) annealing at 80 °C for 30 min. Scale bar, 300 nm.

Revision: We have now updated Supplementary Fig. 4 in the Supplementary Information of the revised version.

R3Q3. The authors attribute the constrained maximum radiance in multijunction devices to Joule heating, as evidenced by the thermal imaging and simulations in Supplementary Fig. 14. Nevertheless, the detailed explanation in the main text is limited. The manuscript would be strengthened by including temperature characteristics for the four- and five-junction PeLEDs, along with a brief discussion elucidating the origin of the radiance decline.

Response: We thank the reviewer for this important comment. In the original manuscript, the results in Supplementary Fig. 14 showed infrared thermal images and simulated temperature profiles for single-, double-, and triple-junction devices. Following the reviewer's suggestion, we have now performed the same measurements and simulations for four- and five-junction $FAPbI_3$ PeLEDs. As shown in Fig. R5, the operating temperature of four- and five-junction devices at 400 mA cm^{-2} is 30-50 °C higher than that of single-junction devices, where intense Joule heating promotes non-radiative recombination and accelerates efficiency roll-off. Additionally, as we show in **Fig. R1**, the solution-processed fabrication of stacked perovskite subunits requires repeated thermal annealing treatments, which inevitably degrade the performance of underlying device layers. Overall, the performance deterioration caused by repetitive thermal annealing during device fabrication, together with Joule heat accumulation during working operation, jointly lead to the obvious reduction in radiance of multijunction PeLEDs.

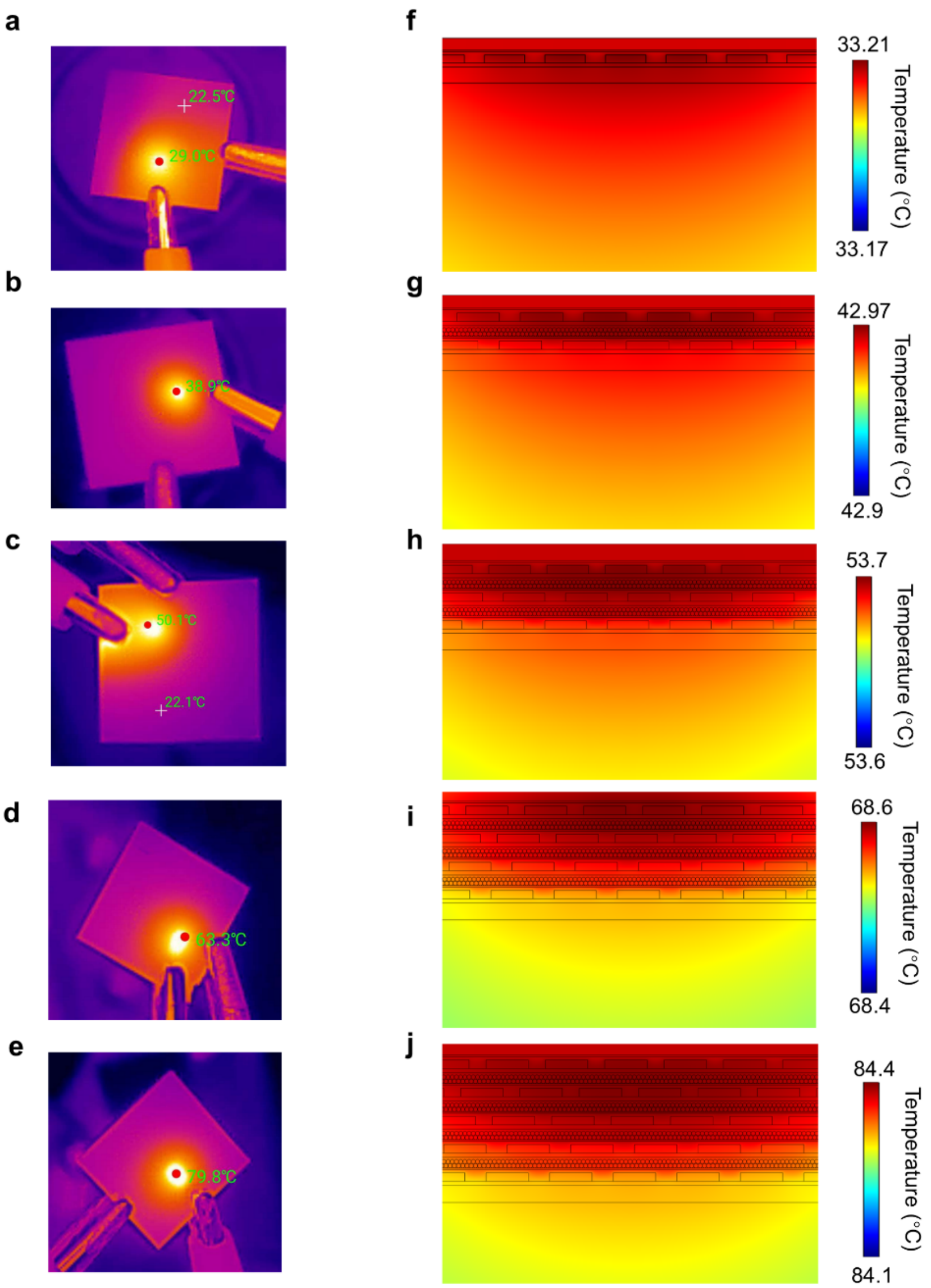

Fig. R5 | The temperature characteristics of single- and multi-junction tandem PeLEDs. a-e, Infrared-thermal-imaging pictures of the single- (**a**), double- (**b**), triple-junction (**c**), four- (**d**), five-junction (**e**) PeLEDs after operating at a current density of 400 mA cm^{-2} for 10 s. **f-j**, Simulated cross-sectional temperature profiles of the single- (**f**), double- (**g**), triple-junction (**h**), four- (**i**), and five-junction (**j**) PeLEDs.

Revision: We have added a brief discussion elucidating the origin of the radiance decline in the main text and updated the thermal images and simulated temperature profiles for four- and five-junction PeLEDs in Supplementary Fig. 14.

R3Q4. ALD-SnO_2 is a key component of the ICL, providing both solvent protection and electron transport. While the Methods section only states that the ALD-SnO_2 was

deposited at 80 °C, additional deposition details and parameters are needed to ensure reproducibility.

Response: We apologize for missing detailed ALD parameters in the original manuscript. We have now provided a complete description of the ALD process for SnO_2 deposition in the methods section as follows:

Chamber pressure: 0.1 Torr

Substrate temperature: 80 °C

Precursors: tetrakis(dimethylamido)tin(IV) (TDMASn, maintained at 70 °C during the ALD process), deionized water

One deposition cycle: TDMASn pulse (0.18 s) - N_2 purge (5 s) - H_2O pulse (0.03 s) -N_2 purge (5 s)

Total cycles for 20 nm: 170 cycles

Revision: We have now added the ALD-SnO_2 fabrication details in the methods section of the revised version.

R3Q5. Several minor language errors and typos should be corrected. For instance:

•On Page 3, “and can substantially prolonged operational lifetime” should be “and can substantially prolong operational lifetime.”

•On Page 6, “As an key component in tandem LEDs” should be “As a key component in tandem LEDs.” The authors should proofread the manuscript to correct those similar issues.

Response: We apologize for the typos and thank the reviewer for pointing out them. We have comprehensively checked the manuscript and corrected other similar issues. We have now corrected ‘prolonged’ to ‘prolong’ and changed ‘As an key’ to ‘As a key’ in the revised version.

Reference

1. Ke, Y. *et al.* High-performance tandem perovskite LEDs through interlayer photon recycling. *Nature* **649**, 53–58 (2026).
2. Lian, Y. *et al.* Ultralow-voltage operation of light-emitting diodes. *Nat. Commun.* **13**, 3845 (2022).
